# Changes in health in the countries of the UK and 150 English Local Authority areas 1990–2016: a systematic analysis for the Global Burden of Disease Study 2016

**DOI:** 10.1016/S0140-6736(18)32207-4

**Published:** 2018-11-03

**Authors:** Nicholas Steel, John A Ford, John N Newton, Adrian C J Davis, Theo Vos, Mohsen Naghavi, Scott Glenn, Andrew Hughes, Alice M Dalton, Diane Stockton, Ciaran Humphreys, Mary Dallat, Jürgen Schmidt, Julian Flowers, Sebastian Fox, Ibrahim Abubakar, Robert W Aldridge, Allan Baker, Carol Brayne, Traolach Brugha, Simon Capewell, Josip Car, Cyrus Cooper, Majid Ezzati, Justine Fitzpatrick, Felix Greaves, Roderick Hay, Simon Hay, Frank Kee, Heidi J Larson, Ronan A Lyons, Azeem Majeed, Martin McKee, Salman Rawaf, Harry Rutter, Sonia Saxena, Aziz Sheikh, Liam Smeeth, Russell M Viner, Stein Emil Vollset, Hywel C Williams, Charles Wolfe, Anthony Woolf, Christopher J L Murray

**Affiliations:** a University of East Anglia, Norwich, UK; b Public Health England, London, UK; c AD CAVE Solutions Ltd, London, UK; d Imperial College London, London, UK; e Institute for Health Metrics and Evaluation, Seattle, WA, USA; f Public Health England, Oxford, UK; g NHS Health Scotland, Edinburgh, UK; h Public Health Wales, Carmarthen, UK; i Public Health Agency, Belfast, UK; j University College London, London, UK; k Cambridge Institute of Public Health, University of Cambridge, Cambridge, UK; l Department of Health Sciences, College of Life Sciences, University of Leicester, Leicester, UK; m Department of Public Health & Policy, Institute of Psychology, Health & Society, University of Liverpool, Liverpool, UK; n Centre for Population Health Sciences, Lee Kong Chian School of Medicine, Nanyang Technological University, Singapore, Singapore; o MRC Lifecourse Epidemiology Unit, University of Southampton, Southampton, UK; p NIHR Southampton Biomedical Research Centre, University of Southampton and University Hospital Southampton NHS Foundation Trust, Southampton, UK; q NIHR Oxford Biomedical Research Centre, University of Oxford, Oxford, UK; r King's College London, London, UK; s UKCRC Centre of Excellence for Public Health Research (NI), Queens University of Belfast, Belfast, UK; t London School of Hygiene & Tropical Medicine, London, UK; u Health Data Research UK, Swansea University, Swansea, UK; v University of Bath, Bath, UK; w Usher Institute of Population Health Sciences and Informatics, University of Edinburgh, Edinburgh, UK; x Centre of Evidence-Based Dermatology, Queen's Medical Centre, Nottingham University Hospitals NHS Trust, Nottingham, UK; y Bone and Joint Research Group, Royal Cornwall Hospital, Truro, UK

## Abstract

**Background:**

Previous studies have reported national and regional Global Burden of Disease (GBD) estimates for the UK. Because of substantial variation in health within the UK, action to improve it requires comparable estimates of disease burden and risks at country and local levels. The slowdown in the rate of improvement in life expectancy requires further investigation. We use GBD 2016 data on mortality, causes of death, and disability to analyse the burden of disease in the countries of the UK and within local authorities in England by deprivation quintile.

**Methods:**

We extracted data from the GBD 2016 to estimate years of life lost (YLLs), years lived with disability (YLDs), disability-adjusted life-years (DALYs), and attributable risks from 1990 to 2016 for England, Scotland, Wales, Northern Ireland, the UK, and 150 English Upper-Tier Local Authorities. We estimated the burden of disease by cause of death, condition, year, and sex. We analysed the association between burden of disease and socioeconomic deprivation using the Index of Multiple Deprivation. We present results for all 264 GBD causes of death combined and the leading 20 specific causes, and all 84 GBD risks or risk clusters combined and 17 specific risks or risk clusters.

**Findings:**

The leading causes of age-adjusted YLLs in all UK countries in 2016 were ischaemic heart disease, lung cancers, cerebrovascular disease, and chronic obstructive pulmonary disease. Age-standardised rates of YLLs for all causes varied by two times between local areas in England according to levels of socioeconomic deprivation (from 14 274 per 100 000 population [95% uncertainty interval 12 791–15 875] in Blackpool to 6888 [6145–7739] in Wokingham). Some Upper-Tier Local Authorities, particularly those in London, did better than expected for their level of deprivation. Allowing for differences in age structure, more deprived Upper-Tier Local Authorities had higher attributable YLLs for most major risk factors in the GBD. The population attributable fractions for all-cause YLLs for individual major risk factors varied across Upper-Tier Local Authorities. Life expectancy and YLLs have improved more slowly since 2010 in all UK countries compared with 1990–2010. In nine of 150 Upper-Tier Local Authorities, YLLs increased after 2010. For attributable YLLs, the rate of improvement slowed most substantially for cardiovascular disease and breast, colorectal, and lung cancers, and showed little change for Alzheimer's disease and other dementias. Morbidity makes an increasing contribution to overall burden in the UK compared with mortality. The age-standardised UK DALY rate for low back and neck pain (1795 [1258–2356]) was higher than for ischaemic heart disease (1200 [1155–1246]) or lung cancer (660 [642–679]). The leading causes of ill health (measured through YLDs) in the UK in 2016 were low back and neck pain, skin and subcutaneous diseases, migraine, depressive disorders, and sense organ disease. Age-standardised YLD rates varied much less than equivalent YLL rates across the UK, which reflects the relative scarcity of local data on causes of ill health.

**Interpretation:**

These estimates at local, regional, and national level will allow policy makers to match resources and priorities to levels of burden and risk factors. Improvement in YLLs and life expectancy slowed notably after 2010, particularly in cardiovascular disease and cancer, and targeted actions are needed if the rate of improvement is to recover. A targeted policy response is also required to address the increasing proportion of burden due to morbidity, such as musculoskeletal problems and depression. Improving the quality and completeness of available data on these causes is an essential component of this response.

**Funding:**

Bill & Melinda Gates Foundation and Public Health England.

**Research in context**

**Evidence before this study**

The Global Burden of Disease (GBD) study has described the contribution of fatal and non-fatal conditions to the burden of disease internationally and shown the importance of understanding geographical variations in disease, risk factors, and socioeconomic deprivation. GBD has been used to generate subnational estimates in several countries to inform local priorities and practice. In the UK, policy and action to improve health require comparable estimates of disease burden and risks at national and local authority levels. To date, only regional estimates have been available from previous rounds of GBD. Improvements in mortality have slowed in the UK and other countries over a timescale that could imply a link with political, economic, and service factors in the UK. However, similar changes have been seen in some other countries and the causes of the change in the UK remain unknown. As a result, the required policy response remains uncertain. It has been shown that sustained public health interventions at the population level can be effective and that benefits accrue both from prevention and improved treatment from health services.

**Added value of this study**

We compare the contributions of individual conditions to years of life lost in the UK for current policy-relevant geographies, the largest contributions being for ischaemic heart disease, lung cancers, cerebrovascular disease, and chronic obstructive pulmonary disease. The extent to which the burden due to these conditions is attributed to specific potentially preventable risks is also quantified (eg, for tobacco use, poor diet, alcohol, obesity, and air pollution). Variation in burden between local areas is described and shown to relate strongly to deprivation. Opportunity to reduce burden due to premature mortality by addressing specific risk factors is also shown to correlate strongly with deprivation. Non-fatal conditions are identified as increasingly important contributors to overall burden across the UK, particularly low back and neck pain, skin diseases, migraine, sense organ diseases, and depressive and anxiety disorders. Updated GBD estimates show that the slowed rate of improvement in overall mortality rates in the UK since 2010 appears to be condition specific and largely driven by decreases in the rate of improvement in mortality from cardiovascular disease and certain cancers.

**Implications of all the available evidence**

We describe and quantify the extent to which the UK could reduce the overall burden of fatal and non-fatal conditions through effective prevention. The results identify and rank potential local, regional, and national priorities for action that would reduce burden and provide relevant support for local and national advocacy on such priorities. These estimates should directly inform long-term planning for health in the UK—for example, the 10-year plan for the National Health Service in England from 2019. Social and economic determinants of ill health are an overriding concern. There is a need for economic development and regeneration of poorer parts of the country, and for high-quality health improvement programmes and care services in these areas. As mortality continues to reduce, albeit more slowly than before, ill health due to low back pain, skin diseases, sense organ diseases, and depressive disorders makes an increasing contribution to overall burden of disease. Local estimates of ill health that are used to guide policy and practice could be improved and made more comparable by better use of existing data. Health records and linkage to survey data should be used more extensively to refine disease prevalence estimates, improve consistency between GBD and other sources, and provide more reliable data to guide policy and programmes to address these causes of ill health and their sequelae.

## Introduction

The Global Burden of Disease (GBD) project aims to produce the best possible comparable estimates of ill health and injury around the world.^1^ It is an annual global assessment of the health of populations, broken down by age, sex, country, and selected subnational geographical areas.^2^, ^3^, ^4^, ^5^, ^6^, ^7^ After 20 years of refinement, GBD data makes a unique contribution to health policy and practice worldwide. 8, 9

Previous studies have reported GBD 2010 estimates for the UK,^10^ and GBD 2013 estimates for nine English regions split by deprivation quintile.^11^ GBD estimates of burden of disease have been used extensively at the national level; for example by Public Health England—which is an executive agency of the Department of Health and Social Care—in its strategic planning and its national health profile report,^12^ and by Public Health Wales in its report of Health and its Determinants in Wales,^13^ which informed Public Health Wales' strategic plan and health service planning in Wales. The National Institute for Health Research has used GBD data to assess the balance of their funded research portfolio.^14^ GBD has also been used at a more granular level by bodies with an interest in addressing high burden conditions, such as mental health and musculoskeletal diseases, and by local Directors of Public Health.^15^ Scotland has recently done its own independent analysis of its burden of disease.^16^

Estimates of the burden of disease for smaller geographical areas than whole countries (subnational estimates) to aid local policy and practice is a priority for the GBD project.^17^ In 2015, the UK^11^ and Japan^18^ were the first countries to publish subnational GBD estimates; India followed in 2016,^19^ and subnational estimates have been presented for Brazil, China, Indonesia, Mexico, Russia, and the USA since then. Health policy is devolved in the UK and provision of services differs between the countries of the UK.^20^ In England, Upper-Tier Local Authorities serving populations from 38 169 people (Rutland) to 1 532 102 people (Kent) are responsible for maintaining and improving the health of their populations.^21^ Levels of deprivation vary markedly between Upper-Tier Local Authorities. Local autonomy and scarcity of resources for public health action generate a requirement for national and local estimates of morbidity and mortality to inform priority setting for public health and health services.

Several limitations of previous studies have been addressed in GBD 2016 through technical improvements and updates. These include an expanded GBD cause hierarchy with 18 newly-specified causes of death and many new data sources. Updated GBD mortality information is of interest because improvements in life expectancy have slowed in the UK, USA, Canada, Australia, and most, but not all, other European countries since 2010 for reasons that are unclear. It has been suggested that this effect could be due to reductions in welfare provision or a systemic failure of social and health care in certain areas.^22^, ^23^ It is important, therefore, to understand the nature of the change in more detail.

This paper presents updated GBD 2016 estimates for the UK from 1990 to 2016 and, for the first time, includes results for England, Wales, Scotland, and Northern Ireland, and 150 Upper-Tier Local Authorities in England. These latest GBD results might help to explain the causes of the slowdown in the improvements in life expectancy since 2010, and will be a guide to rational priority setting for health and social policy, prevention policy, health service planning, and research at national and local levels.

## Methods

### Overview

The GBD study is a standardised analytical approach for estimating life expectancy, years of life lost due to premature mortality (YLLs), years lived with disability (YLDs), disability-adjusted life-years (DALYs), and risk factors. We aim to use all accessible information in the UK, England, Scotland, Wales, Northern Ireland, and 150 English Upper-Tier Local Authorities from 1990 to 2016 for each location, age group, sex, and year. There are 152 Upper-Tier Local Authorities in England, including county councils, London boroughs, unitary authorities, and metropolitan districts. We excluded the City of London and Isles of Scilly from this analysis because of their small populations; therefore, data were available for 150 English Upper-Tier Local Authorities.

### Years of life lost and causes of death

YLLs were computed by multiplying the number of estimated deaths by the standard life expectancy at age of death, which was derived from the lowest observed mortality in any population in the world greater than 5 million (86·6 years at birth for GBD 2016).^2^, ^4^ Causes of death were mapped to the 264 GBD 2016 causes of death and age and sex groups. Causes of death were organised in a four-level hierarchy that covered all deaths at all ages. The three cause groups at level 1 were communicable, maternal, neonatal, and nutritional diseases; non-communicable diseases; and injuries. These were broken down into level 2 causes with further disaggregation into level 3 and 4 causes. Ischaemic stroke, for example, was classified as non-communicable diseases (level 1), cardiovascular diseases (level 2), cerebrovascular disease (level 3), and ischaemic stroke (level 4).^3^, ^4^

We compared causes of death at level 3 in the hierarchy to provide a meaningful level for policy makers and health professionals. The exception is cirrhosis, a GBD level 2 cause, which we show as a level 3 cause because further disaggregation into cirrhosis caused by hepatitis, alcohol, or other gives more granular detail than is required for comparison with other level 3 causes. Mortality was based on year of registration. In England and Wales, some deaths might be registered in subsequent years because of delays caused by coroners' inquests; some of this lag was taken into account in the modelling process, which smoothes over time. All imprecise causes of death, for example ill-defined cancer site or senility, were redistributed to the most likely alternative GBD cause of death.^3^ Estimates for each location, year, age, and sex were generated using Cause of Death Ensemble modelling (CODEm), which chose an ensemble of models that best reflected all the input data.^3^ The resulting estimates were rescaled so that the sum of all cause-specific deaths equalled the total number of deaths from all causes in each age, sex, location, and year category.^3^

### Years lived with a disability

YLDs were estimated by multiplying the prevalence of each cause and its consequences by a disability weight, corrected for comorbidity.^4^ The prevalence of each condition was estimated from published papers, unpublished documents, survey microdata, administrative records of health encounters, registries, and disease surveillance systems. Data availability and use by GBD varied between countries. Data sources are available in the appendix (pp 2–20) for the different constituent nations in the UK for diabetes, chronic obstructive pulmonary disease, low back and neck pain, skin conditions and depression, and, in full, from the GBD Data Input Sources Tool.^24^

DisMod-MR 2.1, a Bayesian meta-regression tool, was used to estimate YLDs whilst ensuring consistency between incidence, prevalence, remission, and cause of death rates for each condition.^4^, ^25^ A first model was run on global GBD data, which produced an initial global fit and estimated coefficients for predictor variables. The global fit was adjusted for predictors and passed down through the GBD geographical levels to country level and then to Upper-Tier Local Authorities in England in 2016. Disease-specific YLDs were adjusted for comorbidity. We predicted estimates for locations with few or no data by borrowing information (for example on prevalence of a condition) from other locations, and using covariates to generate comparable estimates across all geographical locations.

We created maps of the UK for the changes in all-cause, age-standardised YLLs and YLDs over three time periods (1990–99, 2000–09, and 2010–16), using UK census boundary data^26^ and geographical information software (ArcGIS 10·3, ESRI, Redlands, CA, USA).^27^ We measured deprivation in Upper-Tier Local Authorities using the Index of Multiple Deprivation (IMD-2015), which is a composite measure estimated at small geographical areas that includes seven domains: income, employment, education, health, crime, barriers to housing and services, and living environment.^28^ We calculated correlations between IMD score and YLLs using Pearson's correlation coefficients on a scale from 1 (perfect positive correlation) to −1 (perfect negative correlation), where scatter plots showed a linear correlation.

### Disability-adjusted life-years

DALYs are the sum of YLLs and YLDs for each location, age group, sex, and year.^5^ Risk factor exposure and attributable risk were estimated for 84 behavioural, environmental and occupational, and metabolic risks or clusters of risks.^6^ 481 risk–outcome pairs met the GBD study criteria for convincing or probable evidence of a specific risk causing a specific outcome. We estimated the attributable burden for each risk by multiplying the YLLs and YLDs for each outcome of interest by the population attributable fraction (PAF) for the risk–outcome pair.^6^ The PAF represents the proportion of DALYs that would have been avoided in a given year if the exposure to a risk factor in the past had been at the theoretical minimum risk exposure level.

### Role of the funding source

The GBD 2016 database development, methods improvement, and global analysis is primarily funded by the Bill & Melinda Gates Foundation, which had no role in study design, data collection, data analysis, data interpretation, or writing of the report. The corresponding author had full access to all data in the study and had final responsibility to submit the paper.

## Results

**Figure 1:**
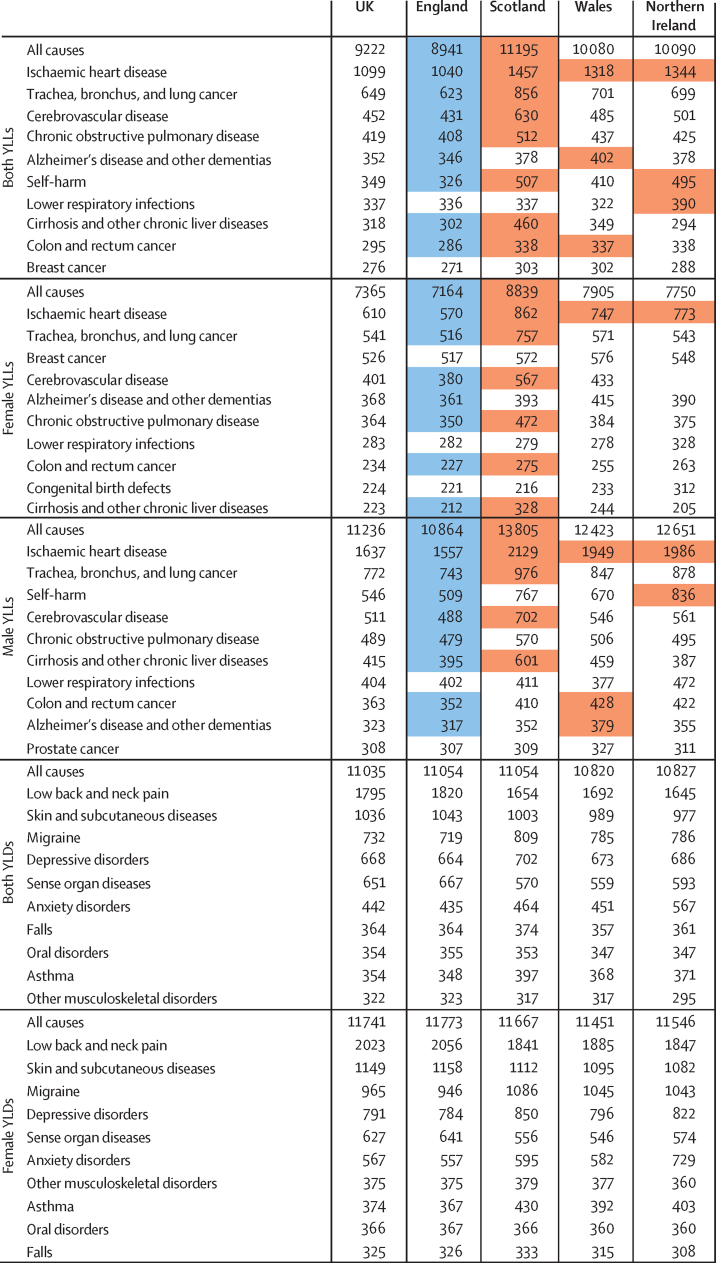

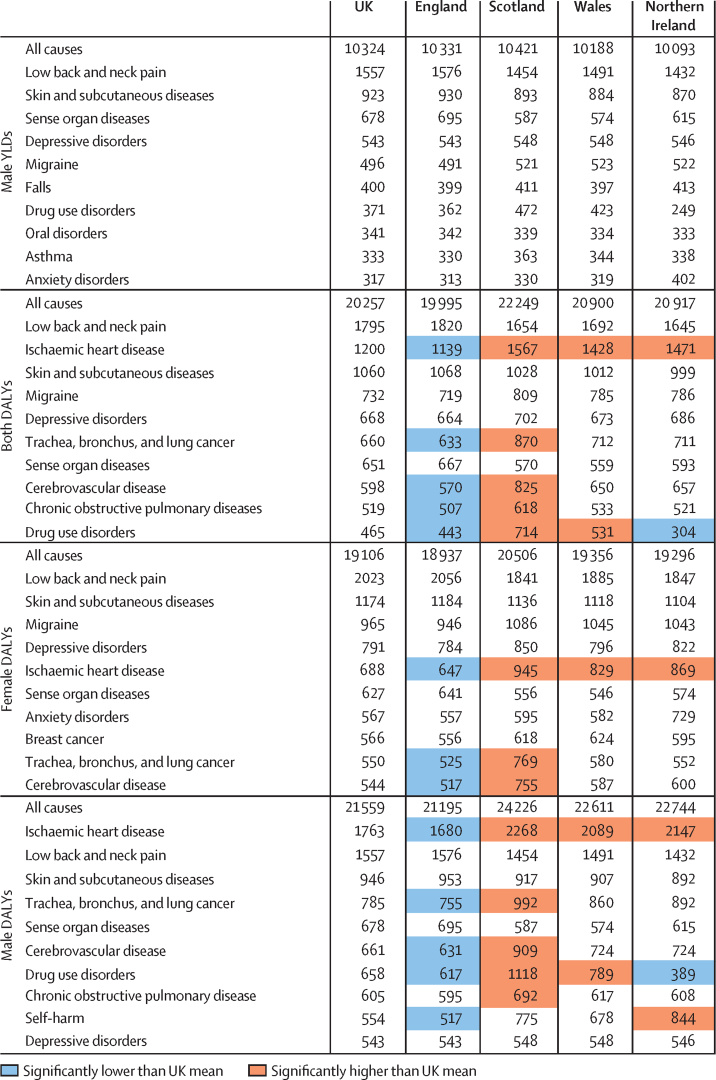
Age-standardised YLL, YLD, and DALY rates per 100 000 population for all causes combined and leading ten causes in UK countries, women, men, and both sexes, 2016 YLLs=years of life lost. YLDs=years lived with disability. DALY=disability-adjusted life-year.

The leading causes of YLLs in the UK in 2016 for both sexes combined were ischaemic heart disease, trachea, bronchus, and lung cancer (subsequently referred to as lung cancer), cerebrovascular disease, chronic obstructive pulmonary disease, and Alzheimer's disease and other dementias (subsequently referred to as dementia; figure 1). The most common causes of disability (measured by YLDs) were low back and neck pain, skin and subcutaneous diseases, migraine, depressive disorders, and sense organ diseases (figure 1). The highest burden of age-standardised DALYs in both sexes combined was for low back and neck pain (1795 [95% uncertainty interval 1258–2356] per 100 000 population per year), followed by ischaemic heart disease (1200 [1155–1246]; figure 1). By contrast, the DALY rates were 660 (642–679) for lung cancer, 598 (550–640) for cerebrovascular disease, and 519 (487–561) for chronic obstructive pulmonary disease.

The all-cause age-standardised YLL rate in 2016 was highest in Scotland (11 195 [10 177–12 389] per 100 000 population) and lowest in England (8941 [8847–9028]), with ischaemic heart disease, lung cancer, cerebrovascular disease, and chronic obstructive pulmonary disease highest in Scotland (figure 1). Age-standardised YLDs were highest in England (11 054 [8211–14 261]) and Scotland (11 054 [8188–14 304]) and lowest in Wales (10 820 [8030–14 039]); however, the range of YLDs across the UK countries only varied by 234 per 100 000 population per year compared with a range of 2254 per 100 000 population per year for YLLs. England had the highest YLD rates for low back and neck pain, skin conditions and sense organ disease, and anxiety was highest in Northern Ireland.

Many conditions were important contributors to burden for both sexes, but there were differences. For YLLs, men had higher rates for all ten leading conditions than did women, except for dementia and breast cancer. Ischaemic heart disease was the leading cause of YLLs in both sexes, yet the rate was about 2·5 times higher in men than it was in women. Self-harm was the third highest YLL for men (546 [422–596]), but was fourteenth highest for women (153 [146–162]). Prostate cancer and breast cancer were important causes of premature mortality for both sexes, but breast cancer YLLs ranked higher for women than prostate cancer did for men. For YLDs, women had higher rates of disability for all the ten leading conditions than did men, except for sense organ diseases, falls, and drug use disorders.

**Figure 2:**
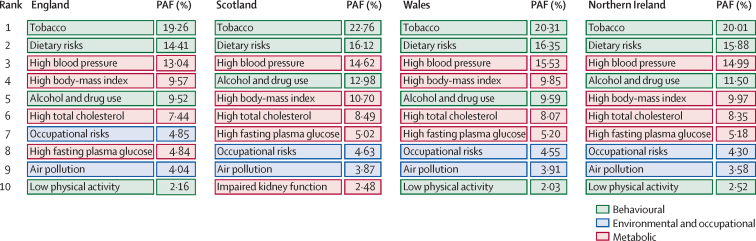
PAF for risk factors for all-cause YLLs rate per 100 000 population for England, Scotland, Wales, and Northern Ireland, both sexes, 2016 PAF=population attributable fraction. YLLs=years of life lost.

The ten leading risk factors contributing to YLLs were similar in rank across the four countries of the UK (figure 2). Although the ranks were similar, the PAF of each risk factor varied in size in different countries, such as a higher PAF from tobacco in Scotland, and from alcohol and drug use in Scotland and Northern Ireland, compared with the other UK nations.

**Figure 3:**
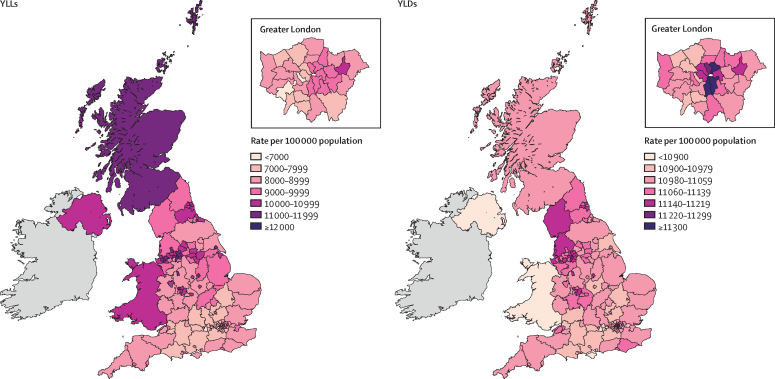
All-cause age-standardised YLL and YLD rates per 100 000 population by UK country and English Upper Tier Local Authorities, 2016 YLLs=years of life lost. YLDs=years lived with disability.

**Figure 4:**
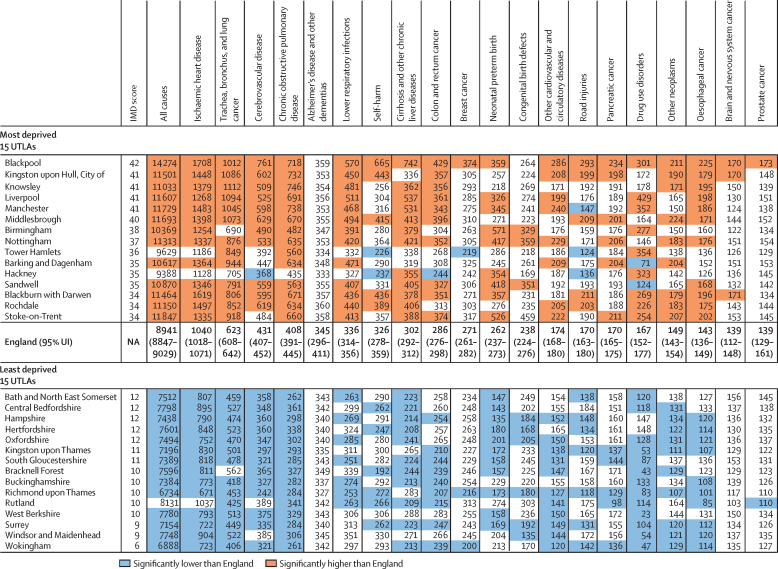
Age-standardised YLL rate per 100 000 people for the 20 causes with the highest national YLL burden (order of decreasing burden), in the 15 (10%) most deprived, and 15 (10%) least deprived UTLAs in England, both sexes, 2016 YLLs=years of life lost. UTLAs=Upper-Tier Local Authorities. IMD=Index of Multiple Deprivation. UI=uncertainty interval. NA=not applicable.

In England in 2016, age-standardised rates of YLLs for all causes varied by more than two times between the highest and lowest IMD-ranked Upper-Tier Local Authorities (14 274 [12 791–15 875] per 100 000 people in Blackpool *vs* 6888 [6145–7739] in Wokingham; Figure 3, Figure 4). Age-standardised YLL rates for the 15 (10%) most deprived and 15 least deprived Upper-Tier Local Authorities in England (figure 4) were consistently increased in the deprived areas for most conditions. This association with deprivation was clearest for all causes, lung cancer, and chronic obstructive pulmonary disease. No association was seen with dementia, breast cancer, self-harm, congenital birth defects, and neonatal preterm birth complications (appendix p 21). Some Upper-Tier Local Authorities did better on YLLs than was expected from their level of deprivation, including Birmingham and the London boroughs of Tower Hamlets, Hackney, and Barking and Dagenham (figure 4). Notably, Upper-Tier Local Authorities in London had generally lower rates of DALYs and YLLs than was expected for their level of deprivation (appendix pp 22–29).

**Figure 5:**
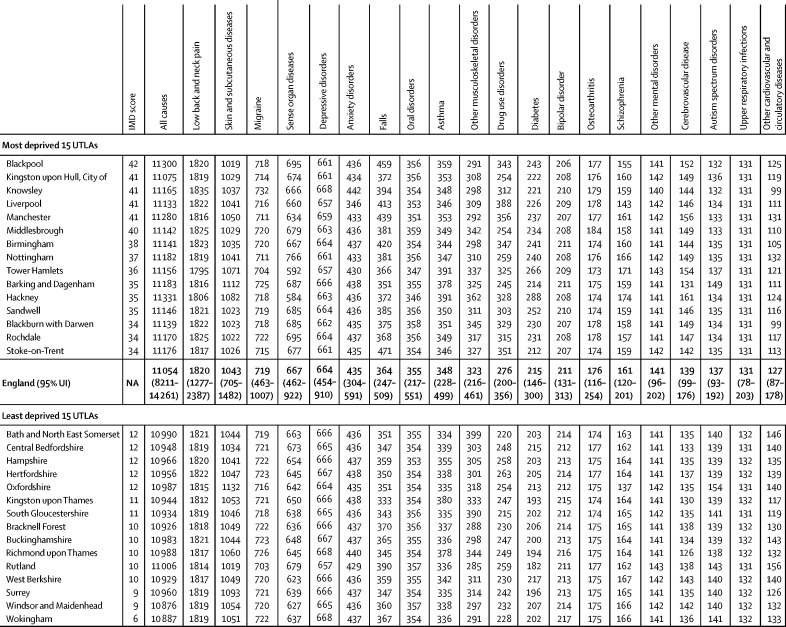
Age-standardised YLD rate per 100 000 population for the 20 causes with the highest national YLD burden (order of decreasing burden), in the 15 (10%) most deprived, and 15 (10%) least deprived UTLAs in England, both sexes, 2016 No estimates were significantly different from the mean for England. YLDs=years lived with disability. UTLAs=Upper-Tier Local Authorities. IMD=Index of Multiple Deprivation. UI=uncertainty interval. NA=not applicable.

Age-standardised YLD rates varied much less than YLLs did, with no significant variations (Figure 3, Figure 5; appendix pp 30–33).

**Figure 6:**
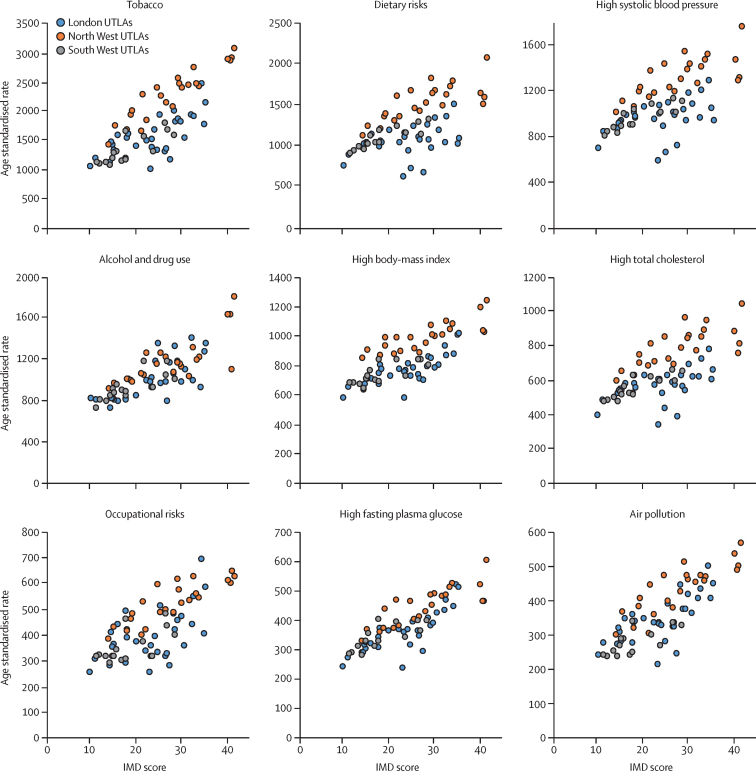
Attributable risk for age-standardised all-cause YLL rate per 100 000 population for nine major risk factors, and UTLA level IMD score, for UTLAs in three regions of England, 2016 YLL=years of life lost. UTLAs=Upper-Tier Local Authorities. IMD=Index of Multiple Deprivation.

More deprived Upper-Tier Local Authorities had higher age-standardised attributable burden of age-standardised all-cause YLLs than did less deprived Upper-Tier Local Authorities for most risk factors, although there was variation within and between regions (figure 6; appendix pp 34–39). The PAF for risk factors also varied by Upper-Tier Local Authority for a given level of deprivation (appendix pp 40–44). For example, London Upper-Tier Local Authorities had lower attributable YLL burdens than were expected, particularly for tobacco, dietary risks, and high body-mass index; whereas the association between deprivation and alcohol and drug use and occupational risks, varied less between regions (figure 6).

**Figure 7:**
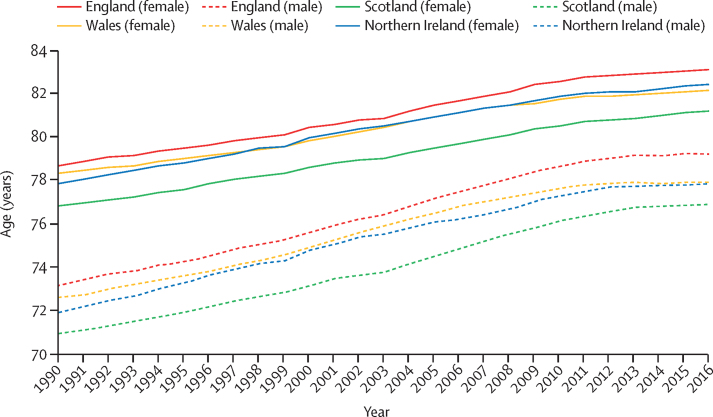
Life expectancy at birth for England, Scotland, Wales, and Northern Ireland 1990–2016, by sex

**Figure 8:**
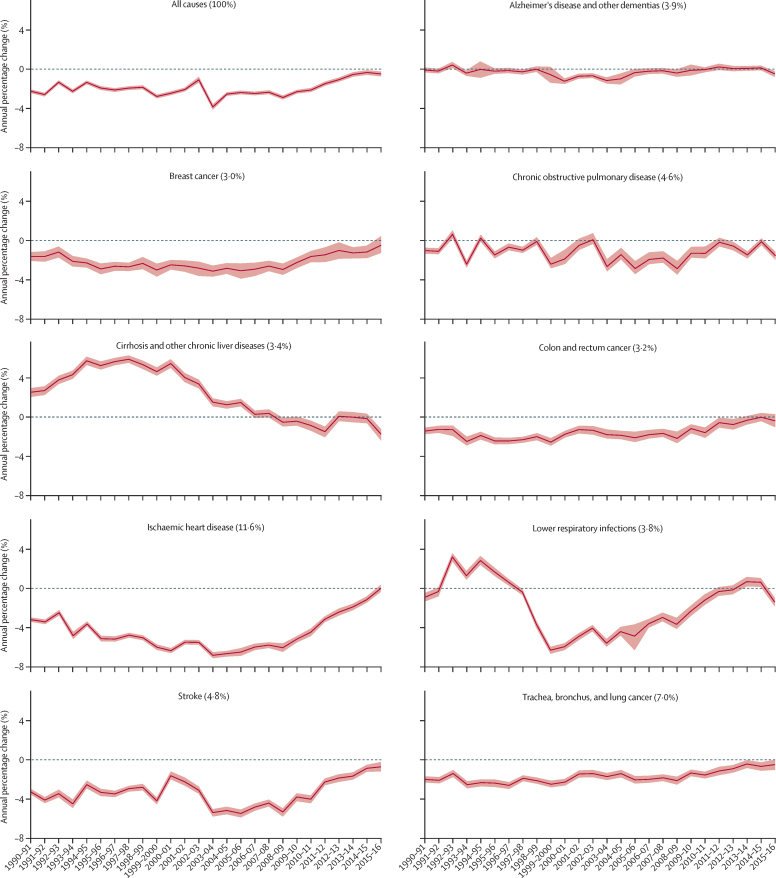
Annual percentage change in YLL rate per 100 000 people for the nine causes with the highest national burden, 1990–2016 in England Ribbons are 95% uncertainty intervals. The percentage contribution of each condition to all-cause YLLs is given in brackets. YLLs=years of life lost.

Between 1990 and 2016, life expectancy at birth for both sexes improved in all four UK countries, but the rate of improvement has slowed since 2010 (figure 7). Although random variation is expected, nine out of 150 English Upper-Tier Local Authorities had increased YLL rates since 2010, compared to none in 2000–09 (appendix p 45). The trends in annual change in age-standardised YLLs differed when disaggregated by cause. The reduction in the annual rate of improvement for all-cause YLLs since 2010 was driven by the gradual disappearance of sustained annual improvements in YLL rates from ischaemic heart disease, cerebrovascular disease (stroke) and, to a lesser extent, colorectal cancer, lung cancer, and breast cancer (figure 8). Dementia and chronic obstructive pulmonary disease showed no consistent trend in the rate of change over the period. The flattening of the improvement curve for cardiovascular disease deaths was seen in most age groups but was most apparent in those aged over 85 years, where previous improvements had been greatest (appendix pp 46–47).

These cause-specific changes in YLL rates are reflected in the risk factor specific estimates of attributable burden. Annual reduction in all-cause age-standardised YLLs attributable to most major risk factors has also slowed since 2010, except for alcohol and drug use which has remained roughly unchanged since 2000 (appendix p 48). The relation between rate of improvement in YLL and deprivation in Upper-Tier Local Authorities has shifted somewhat over time (appendix pp 49–50). In the period up to 1999, improvement in YLLs were greatest in more affluent Upper-Tier Local Authorities (Pearson's correlation coefficient *r*=0·53); from 2000 to 2009, there was no overall relation between rate of improvement and deprivation; from 2010 to 2016, annual improvement has been greater in more deprived Upper-Tier Local Authorities (*r*=–0·50).

## Discussion

### Burden of disease across the UK

The common causes of premature death in 2016 are similar among the four UK countries. However, premature mortality remains substantially higher in Scotland than it is in England, with higher rates for YLLs from the leading ten causes of death, particularly cardiovascular disease, cancer, and cirrhosis. Wales and Northern Ireland have YLL rates in between those of England and Scotland, with some variation by cause. Comparisons between UK countries at this level, however, masks substantial variation within those countries—for example, between subnational geographical areas in Scotland^29^ and England.^11^

Differences between UK countries are generally less for YLDs than for YLLs, with one notable exception being the high rates of YLDs for anxiety disorders in Northern Ireland. This abundance of mental health conditions in Northern Ireland has been attributed to the social and economic legacy of civil conflict.^30^, ^31^ Across the countries of the UK, burden due to YLDs surpassed that of YLLs in England in 2003, in Wales in 2008, and in Northern Ireland in 2009; whereas, in Scotland, YLLs were similar to that of YLDs in 2016. As death rates decrease, people continue to live with long-term, often multiple, conditions, and YLDs increase. This pattern is a considerable and familiar challenge to statutory health and care services. The attenuation or mitigation of the effect of this rising burden due to non-fatal conditions, and the consequent demand for services, is key to the provision of sustainable services in the future.

Patterns shown in Figure 2, Figure 3 suggest that these long-standing differences between the countries of the UK are likely to be substantially due to variations in risk factors and socioeconomic deprivation, rather than differences in health service organisation and spending. Public health policy and commissioning practices in England differ from those in Scotland, Wales, and Northern Ireland; spending on health services has historically been lower in England than in other UK countries, because of differing funding formulas in the different nations in the UK.^32^, ^33^, ^34^, ^35^ However, wider determinants of health, such as employment opportunities, housing quality and availability, social cohesion and access to good quality education, will probably have a greater effect on health in localities than UK National Health Service (NHS) spending will.

Our results sometimes differ from those of a study^16^ of burden of disease in Scotland, which used different data sources and methods reflecting its country-specific purpose. For example, GBD calculated YLLs using the lowest observed mortality rates in the world and the World Standard Population, whereas the Scottish burden of disease study^16^ used Scottish mortality rates and the European Standard population. The GBD method estimated a YLL for Scotland of 16 891 compared with 13 506 in the Scottish study.^16^, ^36^ The Scottish study used local data to estimate YLDs, resulting in different relative ranks for conditions and larger deprivation gradients in YLDs than was seen in our Upper-Tier Local Authority analyses.^37^

### English Upper-Tier Local Authority estimates

These new local GBD estimates are a highly valuable resource, providing comparable detail down to the local level that can support various local, regional, and national actions. They could be highly informative for regional transformation programmes currently embodied as Sustainability and Transformation Partnerships and for developing Integrated Care Systems across England as the NHS in England delivers its 10-year long-term plan from 2019. They also show the strong and persisting association between deprivation and premature mortality that varies by condition. Action, which might be local or national, is essential to tackle the social and structural drivers of ill health if overall health is to improve. Such action is feasible and can result in rapid improvements in health, with reductions in mortality potentially achievable within 1–2 years.^38^ Notably, population attributable fractions for major risks vary considerably between areas, even within the same region. These findings will be of interest to local public health leaders and should help to set local priorities for action—recognising that such priorities can never be completely data driven and should reflect local opportunities, assets, and political will. Even for nationally important topics, locally specific data such as these GBD estimates make local advocacy more relevant and persuasive for local policy makers.

As described in previous GBD studies,^11^ London has relatively low mortality for its level of deprivation.^39^ One explanation could be that London Upper-Tier Local Authorities have relatively low levels of risk factor exposures, particularly for tobacco and dietary risks. Other possible factors are the high educational performance of poor children in London,^40^ and selective movement of sicker people out of London and healthy young people into London for work. Access to health services in London might also be a factor, with some evidence that health services in the north east and north west of England are relatively underfunded compared with London.^41^ The low mortality could be, at least partly, related to inaccurate population estimates for London as a consequence of high population turnover from high internal and international migration. Finally, IMD scores might function differently in London; for example, the housing deprivation domain includes a measure of housing affordability, for which London does particularly badly.

Areas of socioeconomic deprivation are present throughout the country but are concentrated in the large conurbations of the north of England.^42^ GBD estimates by Upper-Tier Local Authority show that London and Birmingham both have relatively low attributable risk and YLLs compared with authorities with similar deprivation in the northern cities of Liverpool and Manchester. This finding strengthens the need for specific action to respond to the distinct problems that exist in these northern cities.^42^, ^43^ There are also important within-country ethnic differences in outcomes that are not considered in this analysis, such as relatively high avoidable hospital admissions in south Asian groups, and high preventable mortality in white Scottish people compared with ethnic minority populations.^44^

Local GBD data on YLDs are more difficult to evaluate because YLDs are similar for many important conditions across local areas. The most likely explanation for the large uncertainty around YLDs is the relative scarcity of local data on prevalence of the major causes of disability, resulting in estimates that are modelled from data on neighbouring areas. The wide uncertainty around disability weights also increases the uncertainty around YLDs. By contrast, YLLs are based on annual cause of death data from vital registration that show much less heterogeneity between locations and over time compared with non-fatal data sources. To guide an appropriate response, better local data are needed on causes of disability. These could come from health-care datasets, surveys, or other sources, including covariates. However, utilisation data can be biased because of supply factors (such as unavailability for some populations) and surveys could be expensive.

### Trends in mortality

Mortality statistics show that the long-standing trend for annual improvement in life expectancy in England and Wales has slowed since 2011.^45^ Infant mortality rates have increased slightly since 2014, although they remain historically low.^46^ The latest GBD results confirm this effect for YLLs in all the UK countries. As this change in trend has become established, it has generated considerable speculation about its cause or causes, but little firm evidence. Watkins and colleagues^23^ found an apparent correlation with total public spending on health and on social care. Hiam and colleagues^22^ suggested that, in the absence of other plausible causes, cuts to the UK health and social care system were the most probable explanation. Substantial fluctuations in numbers of deaths from year to year, but not the overall trend, can often be attributed to prevalence of circulating flu.^47^ Raleigh^48^ highlights that many different factors are probably involved, including a cohort effect with the gains from reducing smoking already substantially realised, and the rise of comorbidity. A similar change was seen in various other countries at a similar time,^47^, ^49^ which argues against economic or health service factors that are unique to the UK, and suggests something more fundamental is going on related to trends in demography, epidemiology, or socioeconomic factors. The new GBD data reported here show that the change in overall trend is mainly driven by distinct condition-specific trends, predominantly in cardiovascular diseases and some cancers. The worsening trend in YLLs to some cancers is a concern, especially given evidence that survival from some common cancers is already worse in the UK than in some comparable countries.^50^ Population-level factors—such as the global economic crisis since 2008, effect of fiscal austerity in the UK, or the quality and capacity of local services—could affect specific conditions differently through risk factor exposure, health-care provision, or certain social determinants of health.^51^

More affluent areas had greater annual improvements in mortality before 2000, but this changed after 2010 when the national slowdown in mortality improvement was most substantial in affluent areas (appendix pp 45, 49). This is a new finding and differs from evidence from previous recessions that mortality rates improve during economic downturns, perhaps due to declines in risky behaviours.^43^ The reasons for this slowdown in mortality improvement in less deprived areas are unclear. Further research is needed into the association between deprivation and mortality trends since 2010, and the many factors that could contribute, before conclusions can be drawn. Changes in deprivation for older people, unemployment, and binge drinking can explain differences in life expectancy.^52^ These findings suggest that the overall change in trend in YLLs is the result of an evolving epidemiological transition with multiple condition-specific and possibly cohort-based components, including changing exposure to certain risk factors.

### Strengths and limitations

When data were not available for a particular location, GBD modelled estimates using data from other locations and predictive covariates. The availability of accurate local data on mortality was better than for morbidity, which might explain why YLDs varied much less across the UK than did YLLs. Data sources used to produce these 2016 estimates of YLDs for the four UK countries for the example conditions of diabetes, chronic obstructive pulmonary disease, low back and neck pain, skin and subcutaneous diseases, and depressive disorders show that different sources were used for different locations, and therefore some of the variation could be due to different data sources rather than true underlying variation (appendix pp 2–20). A full list of data input sources for GBD 2016 is available online.^24^

When new data or changes in modelling lead to changes in estimates of disease burden, a strength of the GBD approach is that all previous estimates are recalculated with the newest model. For example, the apparent increase in skin disease in GBD 2016 compared with 2013 was due to a change in the method of estimating severity for acne, to award higher disability to a subset of cases with more severe disease (in the past all cases were deemed to have mild disability),^53^ and inclusion of new, more accurate data for dermatitis.

The decision to use a global, European, or UK-specific condition severity distribution affects YLD estimates. The data sources on variations in severity distribution by age or location are sparse, which is a limitation as one would expect substantial variation in severity for conditions with effective treatments. The way deprivation is measured varies across the four countries of the UK, but previous work by Public Health England suggests that this variation does not substantially affect the association between deprivation and prevalence, at least for cancer.^54^ The association between risk factors and outcomes might differ across areas, which could lead to underestimation or overestimation of attributable risk in some areas.^55^

Diabetes, asthma, skin disease, and chronic obstructive pulmonary disease are examples of conditions where GBD and alternative UK estimates differ in 2016. These differences arise mainly from the use of different data sources and disease definitions, and partly from the methods used to model the data in GBD. For example, electronic health record data from primary care in England (The Quality and Outcomes Framework and The Health Improvement Network) and reported data from the Health Survey for England show that prevalence of diabetes is increasing (consistent with other high-income countries),^56^, ^57^, ^58^ whereas GBD used data from research papers for diabetes prevalence, which show a flat or falling prevalence rate.^24^ It has been difficult to reconcile these differences because data governance concerns prevent even anonymised records from UK primary care being made available to the GBD project.

The choice of which GBD level to use when presenting results changes the rank order of conditions. We have presented results by level 3 conditions, which shows low back and neck pain and skin and subcutaneous diseases as the leading causes of YLDs in the UK. At level 2, musculoskeletal and mental disorders are the leading causes (appendix p 51).^3^, ^4^ The distinction between behavioural and metabolic risk factors (figure 2) is not absolute, because behavioural factors (such as physical activity and diet) clearly affect metabolic factors, such as high blood pressure and body-mass index.

### Implications for research and policy

Overall, the results suggest that all countries of the UK could further reduce the burden of disease through effective prevention. For example, the continued dominance of cardiovascular disease in GBD argues for renewed efforts to deliver systematic programmes to reduce risk factors, such as high body-mass index, high fasting glucose, high blood pressure, and high cholesterol. Other conditions that feature highly in GBD estimates for the UK (such as cancers and respiratory disease) can be addressed by tackling specific behaviours, such as smoking and eating unhealthy foods. Good progress has been made in some areas, notably in reducing the prevalence of smoking to historic lows in all countries of the UK, but there is scope to do so much more in almost all areas of primary prevention.

Two-thirds of the improvements to date in premature mortality can be attributed to population-wide decreases in smoking, cholesterol, and blood pressure, and about a third are due to improved therapies.^59^ Health services need to recognise that prevention is a core activity rather than an optional extra to be undertaken if resources allow. In many cases, the causes of ill health and the behaviours that cause it lie outside the control of health services. For example, obesity, sedentary behaviour, and excess alcohol use all feature strongly in GBD as risk factors for diseases such as musculoskeletal disease, liver disease, and poor mental health. The GBD results, therefore, also argue for policies and programmes that deter the food industry from a business model based on cheap calories, that promote and sustain healthy built and natural environments, and that encourage a healthy drinking culture.

The same level of attention that has previously been given to prevention of cardiovascular disease and cancer now needs to be directed at the other major causes of YLLs, such as liver disease and dementia, and associated risk factors, including unhealthy diets, alcohol, air pollution, and drug misuse. Adequate research on effective population-level prevention interventions in these areas is scarce, but not absent.

Public health policy needs also to respond actively and rapidly to the shift in relative burden from mortality towards morbidity. More evidence is needed to support population-level interventions to address the causes and effects of conditions such as musculoskeletal disease, poor mental health, and sensory impairments, and research and action is urgently needed to prevent further increases in burden due to disability from these conditions, and to understand the economic impact. Timely access to health services is important for treatable conditions such as vision loss caused by cataract, glaucoma, and diabetic retinopathy. The promotion of musculoskeletal and mental health are key components of the recent WHO Europe Action Plan for Noncommunicable Disease to avoid premature death and substantially reduce disease burden.^60^

There are still concerns with the accuracy of local estimates of ill health, but the hierarchical ranking of YLD by Upper-Tier Local Authority can inform better local targeting of health services. For future iterations of GBD, the use of primary care electronic health records, including prescribing, should be used to refine disease prevalence estimates and improve consistency between GBD and other reliable estimates, while recognising that utilisation rates have known weaknesses as measures of need.^61^ Data for health-care utilisation remain underutilised for descriptive epidemiology. Their value can be enhanced if linked to population survey data and death records because the strengths of each data type (good diagnostic information in health records, data on risk factors and severity of disease from surveys) enhance their value as a measure of population health. There are excellent examples of data linkage for audit (eg, the Sentinel Stroke National Audit programme), research (eg, the Caliber project at University College London), and policy (eg, NHSDigital linked hospital and mortality data), but still no linked health data that can inform comparable estimates of burden of disease at the local level. Further research on disease burden at the Upper-Tier Local Authority level should explore the burden of different diseases according to specific diagnoses and explore the effect of age disaggregation (eg, in children and in different age groups for older people).

Overall, this study provides timely estimates that can inform the new long-term plan for the NHS in England and similar planning processes in the countries of the UK, and at local level in England. The new local estimates will increase the relevance of GBD for many users, highlighting where local levels of burden and risk factors might require tailored local solutions—for example, for diet and occupational risks (appendix pp 35–44). National results reveal the need for effective primary prevention to reduce the substantial attributable risks due to smoking, unhealthy diets, obesity, and excess alcohol use, which lead to massive burdens from heart disease, cancer, and various comorbidities that reduce independence in older people. Resource allocation in health services needs to continually adapt to the increasing burden from non-fatal conditions, such as musculoskeletal conditions, depressive disorders, sensory loss, and skin diseases. Substantial improvements in the quality and completeness of available morbidity data are needed to support the implementation of such a change in national health policy. We hope that this study will inform similar analyses across Europe supported by the newly formed WHO European Burden of Disease Network.^62^

**This online publication has been corrected. The corrected version first appeared at thelancet.com on October 26, 2018**

## Data sharing
