# Department of Error

**DOI:** 10.1016/S0140-6736(18)32774-0

**Published:** 2018-11-03

*Steel N, Ford JA, Newton JN, et al. Changes in health in the countries of the UK and 150 English Local Authority areas 1990–2016: a systematic analysis for the Global Burden of Disease Study 2016.* Lancet *2018; published online Oct 24.http://dx.doi.org/10.1016/S0140-6736(18)32207-4*—In this Article, an incorrect version of figure 1 was published. This has been corrected online as of Oct 26, 2018, and is correct in the print Article.

