# Supplementary material for: Changes in health in the countries of the UK and 150 English Local Authority areas 1990–2016: a systematic analysis for the Global Burden of Disease Study 2016
Source: Lancet. 2018 Nov 3;392(10158):1647–61. doi: 10.1016/S0140-6736(18)32207-4 (PMC6215773; doi:10.1016/S0140-6736(18)32207-4)
Supplement: Supplementary appendix [file mmc1.pdf]

THE LANCET

Supplementary appendix

This appendix formed part of the original submission and has been peer reviewed. We post it as supplied by the authors.

Supplement to: Steel N, Ford JA, Newton JN, et al. Changes in health in the countries of the UK and 150 English Local Authority areas 1990–2016: a systematic analysis for the Global Burden of Disease Study 2016. *Lancet* 2018; published online Oct 24. http://dx.doi.org/10.1016/S0140-6736(18)32207-4.

Appendix

Title: Changes in health in the countries of the UK and 150 English Local Authority areas 1990–2016: a systematic analysis for the Global Burden of Disease Study 2016

Contents	Page(s)
**Appendix Table 1:** List of data sources used for diabetes across UK countries	2–6
**Appendix Table 2:** List of data sources used for chronic obstructive pulmonary disease (COPD) across UK countries	7
**Appendix Table 3:** List of data sources used for low back and neck pain across UK countries	8–10
**Appendix Table 4:** List of data sources used for skin conditions across UK countries	11–17
**Appendix Table 5:** List of data sources used for depressive disorders across UK countries	18–20
**Appendix Table 6:** Correlation coefficients between Upper Tier Local Authority estimates and Index of Multiple Deprivation (IMD) scores for the 20 causes with the highest national burden for years of life lost (YLLs)in England, 2016	21
**Appendix Figure 1.** Age-standardised disability-adjusted life years (DALYs) rate per 100,000 population, for the 20 causes with the highest DALY burden in order of decreasing national burden and decreasing Upper Tier Local Authority (UTLA) deprivation (Index of Multiple Deprivation [IMD]), England, both sexes, 2016	22–25
**Appendix Figure 2:** Age-standardised years of life lost (YLLs) rate per 100,000 population, for the 20 causes with the highest national YLL burden in order of decreasing burden and decreasing Upper Tier Local Authority (UTLA) deprivation (Index of Multiple Deprivation [IMD] score), England both sexes, 2016	26–29
**Appendix Figure 3:** Age-standardised years lived with disability (YLDs) rate per 100,000 population for the 20 causes with the highest national YLD burden in order of decreasing burden and decreasing Upper Tier Local Authority (UTLA) deprivation (Index of Multiple Deprivation [IMD]), England, both sexes, 2016	30–33
**Appendix Figure 4:** Attributable risk for age-standardised all-cause years of life lost (YLLs) rate per 100,000 population for nine major risk factors, and Upper Tier Local Authority (UTLA) level Index of Multiple Deprivation (IMD) score, for 150 UTLAs in England, 2016	34
**Appendix Figure 5:** Age-standardised attributable risk for all-cause years of life lost (YLLs) rate per 100,000 population in order of decreasing Upper Tier Local Authority (UTLA) deprivation (Index of Multiple Deprivation [IMD]), England, both sexes, 2016	35–39
**Appendix Figure 6:** Population attributable fraction for risk factors for all-cause age-standardised years of life lost (YLLs) rate per 100,000 population per year, in order of decreasing Upper Tier Local Authority (UTLA) deprivation (Index of Multiple Deprivation [IMD]), England, both sexes, 2016	40–44
**Appendix Figure 7.** Annualised percentage change in all-cause age-standardised years of life lost (YLLs) rate over two time periods (2000-2009 and 2010-2016) by UK country and English Upper Tier Local Authorities (UTLA).	45
**Appendix Figure 8:** Absolute change in death rate per 100,000 population over time since 1990 by age group, UK	46
**Appendix Figure 9:** Absolute change in years of life lost (YLL) rate per 100,000 population over time since 1990 by age group, UK	47
**Appendix Figure 10:** Mean annual change in attributable risk for all-cause age-standardised years of life lost (YLLs) rate per 100,000 population over three time periods, 1990-2000, 2000-2010, and 2010-2016 ranked by the highest rate, England	48
**Appendix Figure 11:** Mean annualised percentage change in all-cause age-standardised years of life lost (YLLs) over three time periods compared to Index of Multiple Deprivation (IMD) score, English Upper Tier Local Authorities (UTLAs) for both sexes combined	49
**Appendix Figure 12:** Mean annualised percentage change in all-cause age-standardised years of life lost (YLLs) rate per 100,000 population for the most affluent 10% and the least affluent 10% Upper Tier Local Authorities (UTLAs), England, both sexes, 1990 to 2016	50
**Appendix Figure 13**: Age standardised years lived with disability (YLDs) rate per 100,000 population for the 10 causes with the highest YLD burden by Global Burden of Disease level, United Kingdom both sexes combined, 2016	51
**Data source identification and adjustment**	52–53

Appendix Table 1: List of data sources used for diabetes across UK countries

Citation	Data type
**United Kingdom**	
Joint Health Surveys Unit, University College London and Medical Research Council. Social and Public Health Sciences Unit, Scottish Health Survey, 2003 [computer file]. Colchester, Essex: UK Data Archive [distributor], February 2006. SN: 5318	Survey
Joint Health Surveys Unit of Social and Community Planning Research and University College London, Health Survey for England, 1994 [computer file]. 4th ed. Colchester, Essex: UK Data Archive [distributor], 26 March 2001. SN: 3640	Survey
National Centre for Social Research, University College London Department of Epidemiology and Public Health, Health Survey for England, 1998 [computer file]. 4th ed. Colchester, Essex: UK Data Archive [distributor], 30 November 2002. SN: 4150	Survey
National Centre for Social Research and University College London. Department of Epidemiology and Public Health, Health Survey for England, 1999 [computer file]. 3rd Edition. Colchester, Essex: UK Data Archive [distributor], February 2002. SN: 4365	Survey
National Centre for Social Research, University College London Department of Epidemiology and Public Health, Health Survey for England, 2000 [computer file]. Colchester, Essex: UK Data Archive [distributor], 23 April 2002. SN: 4487	Survey
National Centre for Social Research and University College London. Department of Epidemiology and Public Health, Health Survey for England, 2003 [computer file]. Colchester, Essex: UK Data Archive [distributor], March 2005. SN: 5098	Survey
National Centre for Social Research and University College London. Department of Epidemiology and Public Health, Health Survey for England, 2004 [computer file]. Colchester, Essex: UK Data Archive [distributor], July 2006. SN: 5439	Survey
National Centre for Social Research and University College London. Department of Epidemiology and Public Health, Health Survey for England, 2005 [computer file]. Colchester, Essex: UK Data Archive [distributor], July 2007. SN: 5675	Survey
National Centre for Social Research and University College London. Department of Epidemiology and Public Health, Health Survey for England, 2006 [computer file]. 4th Edition. Colchester, Essex: UK Data Archive [distributor], July 2011. SN: 5809, http://dx.doi.org/10.5255/UKDA-SN-5809-1	Survey
National Centre for Social Research and University College London. Department of Epidemiology and Public Health, Health Survey for England, 2008 [computer file]. 3rd Edition. Colchester, Essex: UK Data Archive [distributor], July 2011. SN: 6397, http://dx.doi.org/10.5255/UKDA-SN-6397-1	Survey
National Centre for Social Research and University College London. Department of Epidemiology and Public Health, Health Survey for England, 2009 [computer file]. 2nd Edition. Colchester, Essex: UK Data Archive [distributor], July 2011. SN: 6732, http://dx.doi.org/10.5255/UKDA-SN-6732-1	Survey
NatCen Social Research and Royal Free and University College Medical School. Department of Epidemiology and Public Health, Health Survey for England, 2010 [computer file]. 2nd Edition. Colchester, Essex: UK Data Archive [distributor], July 2012. SN: 6986, http://dx.doi.org/10.5255/UKDA-SN-6986-2	Survey
NatCen Social Research and University College London. Department of Epidemiology and Public Health, Health Survey for England, 2011 [computer file]. Colchester, Essex: UK Data Archive [distributor], April 2013. SN: 7260, http://dx.doi.org/10.5255/UKDA-SN-7260-1	Survey
Sampson MJ, Shepstone L, Greenwood RH, Harvey I, Humphries J, Heyburn PJ, Temple RC, Dole G. An integrated mobile foot and retinal screening programme for people with Type 2 diabetes managed in primary care. Diabet Med. 2002; 19(1): 74-6	Scientific literature
Walters DP, Gatling W, Mullee MA, Hill RD. The prevalence of diabetic distal sensory neuropathy in an English community. Diabet Med. 1992; 9(4): 349-53	Scientific literature
Edmonds M, Boulton A, Buckenham T, Every N, Foster A, Freeman D, Gadsby R, Gibby O, Knowles A, Pooke M, Tovey F, Unwin N, Wolfe J. Report of the Diabetic Foot and Amputation Group. Diabet Med. 1996; 13(9 Suppl 4): S27-42	Scientific literature

Scottish Centre for Social Research and University College London. Department of Epidemiology and Public Health, Scottish Health Survey, 2008 [computer file]. 2nd Edition. Colchester, Essex: UK Data Archive [distributor], April 2013. SN: 6383, http://dx.doi.org/10.5255/UKDA-SN-6383-2	Survey
Croxson SC, Burden AC, Bodington M, Botha JL. The prevalence of diabetes in elderly people. Diabet Med. 1991; 8(1): 28-31	Scientific literature
Abbott CA, Carrington AL, Ashe H, Bath S, Every LC, Griffiths J, Hann AW, Hussein A, Jackson N, Johnson KE, Ryder CH, Torkington R, Van Ross ERE, Whalley AM, Widdows P, Williamson S, Boulton AJM, North-West Diabetes Foot Care Study. The North-West Diabetes Foot Care Study: incidence of, and risk factors for, new diabetic foot ulceration in a community-based patient cohort. Diabet Med. 2002; 19(5): 377-84	Scientific literature
Canavan RJ, Unwin NC, Kelly WF, Connolly VM. Diabetes- and nondiabetes-related lower extremity amputation incidence before and after the introduction of better organized diabetes foot care: continuous longitudinal monitoring using a standard method. Diabetes Care. 2008; 31(3): 459-63	Scientific literature
Cohen DL, Neil HA, Thorogood M, Mann JI. A population-based study of the incidence of complications associated with type 2 diabetes in the elderly. Diabet Med. 1991; 8(10): 928-33	Scientific literature
Vanderpump MP, Tunbridge WM, French JM, Appleton D, Bates D, Rodgers H, Evans JG, Clark F, Tunbridge F, Young ET. The incidence of diabetes mellitus in an English community: a 20-year follow-up of the Whickham Survey. Diabet Med. 1996; 13(8): 741-7	Scientific literature
Global Lower Extremity Amputation Study Group. Epidemiology of lower extremity amputation in centres in Europe, North America and East Asia. The Global Lower Extremity Amputation Study Group. Br J Surg. 2000; 87(3): 328-37	Scientific literature
Schofield CJ, Yu N, Jain AS, Leese GP. Decreasing amputation rates in patients with diabetes-a population-based study. Diabet Med. 2009; 26(8): 773-7	Scientific literature
Leggetter S, Chaturvedi N, Fuller JH, Edmonds ME. Ethnicity and risk of diabetes-related lower extremity amputation: a population-based, case-control study of African Caribbeans and Europeans in the United kingdom. Arch Intern Med. 2002; 162(1): 73-8	Scientific literature
Department of Epidemiology and Public Health, University College London, National Centre for Social Research (NatCen). United Kingdom Health Survey for England 2013-2014 - HSCIC	Survey
NatCen Social Research and University College London. Department of Epidemiology and Public Health, Health Survey for England, 2012 [computer file]. Colchester, Essex: UK Data Archive [distributor], April 2014. SN: 7480, http://dx.doi.org/10.5255/UKDA-SN-7480-1	Survey
Department of Epidemiology and Public Health, University College London, National Centre for Social Research (NatCen). United Kingdom Health Survey for England 2012-2013 - HSCIC	Survey
Department of Epidemiology and Public Health, University College London, National Centre for Social Research (NatCen). United Kingdom Health Survey for England 2009-2010 - HSCIC	Survey
Department of Epidemiology and Public Health, University College London, MRC Social and Public Health Sciences Unit, University of Glasgow, Scottish Centre for Social Research (ScotCen). United Kingdom - Scottish Health Survey 2011 - Scottish Government	Survey
Department of Epidemiology and Public Health, University College London, MRC Social and Public Health Sciences Unit, University of Glasgow, Scottish Centre for Social Research (ScotCen). United Kingdom - Scottish Health Survey 2010 - Scottish Government	Survey
Department of Epidemiology and Public Health, University College London, MRC Social and Public Health Sciences Unit, University of Glasgow, Scottish Centre for Social Research (ScotCen). United Kingdom - Scottish Health Survey 2009 - Scottish Government	Survey
NatCen Social Research and University College London. Department of Epidemiology and Public Health, Health Survey for England, 2013 [computer file]. Colchester, Essex: UK Data Archive [distributor], January 2015. SN: 7649, http://dx.doi.org/10.5255/UKDA-SN-7649-1	Survey
Kennon B, Leese GP, Cochrane L, Colhoun H, Wild S, Stang D, Sattar N, Pearson D, Lindsay RS, Morris AD, Livingstone S, Young M, McKnight J, Cunningham S. Reduced incidence of lower-extremity amputations in people with diabetes in Scotland: a nationwide study. Diabetes Care. 2012; 35(12): 2588-90	Scientific literature
Krishnan S, Nash F, Baker N, Fowler D, Rayman G. Reduction in diabetic amputations over 11 years in a defined U.K. population: benefits of multidisciplinary team work and continuous prospective audit. Diabetes Care. 2008; 31(1): 99-101	Scientific literature

Morris AD, McAlpine R, Steinke D, Boyle DI, Ebrahim AR, Vasudev N, Stewart CP, Jung RT, Leese GP, MacDonald TM, Newton RW. Diabetes and lower-limb amputations in the community. A retrospective cohort study. DARTS/MEMO Collaboration. Diabetes Audit and Research in Tayside Scotland/Medicines Monitoring Unit. Diabetes Care. 1998; 21(5): 738-43	Scientific literature
New JP, McDowell D, Burns E, Young RJ. Problem of amputations in patients with newly diagnosed diabetes mellitus. Diabet Med. 1998; 15(9): 760-4	Scientific literature
Rayman G, Krishnan ST, Baker NR, Wareham AM, Rayman A. Are we underestimating diabetes-related lower-extremity amputation rates? Results and benefits of the first prospective study. Diabetes Care. 2004; 27(8): 1892-6	Scientific literature
Crawford F, McCowan C, Dimitrov BD, Woodburn J, Wylie GH, Booth E, Leese GP, Bekker HL, Kleijnen J, Fahey T. The risk of foot ulceration in people with diabetes screened in community settings: findings from a cohort study. QJM. 2011; 104(5): 403-10	Scientific literature
Davies M, Brophy S, Williams R, Taylor A. The prevalence, severity, and impact of painful diabetic peripheral neuropathy in type 2 diabetes. Diabetes Care. 2006; 29(7): 1518–22	Scientific literature
Cardwell CR, Carson DJ, Patterson CC. Secular trends, disease maps and ecological analyses of the incidence of childhood onset Type 1 diabetes in Northern Ireland, 1989-2003. Diabet Med. 2007; 24(3): 289-95	Scientific literature
Rangasami JJ, Greenwood DC, McSporran B, Smail PJ, Patterson CC, Waugh NR. Rising incidence of type 1 diabetes in Scottish children, 1984-93. The Scottish Study Group for the Care of Young Diabetics. Arch Dis Child. 1997; 77(3): 210-3	Scientific literature
Betts PR, Logatchov M, Volkov I, Murphy H, Dombrowskaya N, Borzikh S, Ivanova I, Twyman S, Vartan J. An assessment of paediatric diabetes care in three centres in Russia and in Southampton, UK. The Paediatric Teams in Moscow, Tula, Tambov, Southampton. Diabet Med. 1999; 16(9): 772-8	Scientific literature
Gatling W, Budd S, Walters D, Mullee MA, Goddard JR, Hill RD. Evidence of an increasing prevalence of diagnosed diabetes mellitus in the Poole area from 1983 to 1996. Diabet Med. 1998; 15(12): 1015-21	Scientific literature
Patterson CC, Carson DJ, Hadden DR. Epidemiology of childhood IDDM in Northern Ireland 1989-1994: low incidence in areas with highest population density and most household crowding. Northern Ireland Diabetes Study Group. Diabetologia. 1996; 39(9): 1063-9	Scientific literature
Metcalfe MA, Baum JD. Incidence of insulin dependent diabetes in children aged under 15 years in the British Isles during 1988. BMJ. 1991; 302(6774): 443-7	Scientific literature
Staines A, Bodansky HJ, Lilley HE, Stephenson C, McNally RJ, Cartwright RA. The epidemiology of diabetes mellitus in the United Kingdom: the Yorkshire Regional Childhood Diabetes Register. Diabetologia. 1993; 36(12): 1282-7	Scientific literature
Forouhi NG, Luan J, Hennings S, Wareham NJ. Incidence of Type 2 diabetes in England and its association with baseline impaired fasting glucose: the Ely study 1990-2000. Diabet Med. 2007; 24(2): 200-7	Scientific literature
Wadsworth E, Shield J, Hunt L, Baum D. Insulin dependent diabetes in children under 5: incidence and ascertainment validation for 1992. BMJ. 1995; 310(6981): 700-3	Scientific literature
Whisman MA. Loneliness and the metabolic syndrome in a population-based sample of middle-aged and older adults. Health Psychol. 2010; 29(5): 550–4	Scientific literature
**England**	
Canavan RJ, Unwin NC, Kelly WF, Connolly VM. Diabetes- and nondiabetes-related lower extremity amputation incidence before and after the introduction of better organized diabetes foot care: continuous longitudinal monitoring using a standard method. Diabetes Care. 2008; 31(3): 459-63	Scientific literature
Vanderpump MP, Tunbridge WM, French JM, Appleton D, Bates D, Rodgers H, Evans JG, Clark F, Tunbridge F, Young ET. The incidence of diabetes mellitus in an English community: a 20-year follow-up of the Whickham Survey. Diabet Med. 1996; 13(8): 741-7	Scientific literature
Global Lower Extremity Amputation Study Group. Epidemiology of lower extremity amputation in centres in Europe, North America and East Asia. The Global Lower Extremity Amputation Study Group. Br J Surg. 2000; 87(3): 328-37	Scientific literature
Abbott CA, Carrington AL, Ashe H, Bath S, Every LC, Griffiths J, Hann AW, Hussein A, Jackson N, Johnson KE, Ryder CH, Torkington R, Van Ross ERE, Whalley AM, Widdows P, Williamson S, Boulton AJM, North-West Diabetes Foot Care Study. The North-West Diabetes Foot Care Study: incidence of, and risk factors for, new diabetic foot ulceration in a community-based patient cohort. Diabet Med. 2002; 19(5): 377-84	Scientific literature

Staines A, Bodansky HJ, Lilley HE, Stephenson C, McNally RJ, Cartwright RA. The epidemiology of diabetes mellitus in the United Kingdom: the Yorkshire Regional Childhood Diabetes Register. Diabetologia. 1993; 36(12): 1282-7	Scientific literature
Croxson SC, Burden AC, Bodington M, Botha JL. The prevalence of diabetes in elderly people. Diabet Med. 1991; 8(1): 28-31	Scientific literature
Cohen DL, Neil HA, Thorogood M, Mann JI. A population-based study of the incidence of complications associated with type 2 diabetes in the elderly. Diabet Med. 1991; 8(10): 928-33	Scientific literature
Sampson MJ, Shepstone L, Greenwood RH, Harvey I, Humphries J, Heyburn PJ, Temple RC, Dole G. An integrated mobile foot and retinal screening programme for people with Type 2 diabetes managed in primary care. Diabet Med. 2002; 19(1): 74-6	Scientific literature
Leggetter S, Chaturvedi N, Fuller JH, Edmonds ME. Ethnicity and risk of diabetes-related lower extremity amputation: a population-based, case-control study of African Caribbeans and Europeans in the United kingdom. Arch Intern Med. 2002; 162(1): 73-8	Scientific literature
Edmonds M, Boulton A, Buckenham T, Every N, Foster A, Freeman D, Gadsby R, Gibby O, Knowles A, Pooke M, Tovey F, Unwin N, Wolfe J. Report of the Diabetic Foot and Amputation Group. Diabet Med. 1996; 13(9 Suppl 4): S27-42	Scientific literature
Betts PR, Logatchov M, Volkov I, Murphy H, Dombrowskaya N, Borzikh S, Ivanova I, Twyman S, Vartan J. An assessment of paediatric diabetes care in three centres in Russia and in Southampton, UK. The Paediatric Teams in Moscow, Tula, Tambov, Southampton. Diabet Med. 1999; 16(9): 772-8	Scientific literature
Walters DP, Gatling W, Mullee MA, Hill RD. The prevalence of diabetic distal sensory neuropathy in an English community. Diabet Med. 1992; 9(4): 349-53	Scientific literature
Gatling W, Budd S, Walters D, Mullee MA, Goddard JR, Hill RD. Evidence of an increasing prevalence of diagnosed diabetes mellitus in the Poole area from 1983 to 1996. Diabet Med. 1998; 15(12): 1015-21	Scientific literature
Joint Health Surveys Unit of Social and Community Planning Research and University College London, Health Survey for England, 1994 [computer file]. 4th ed. Colchester, Essex: UK Data Archive [distributor], 26 March 2001. SN: 3640	Survey
National Centre for Social Research, University College London Department of Epidemiology and Public Health, Health Survey for England, 1998 [computer file]. 4th ed. Colchester, Essex: UK Data Archive [distributor], 30 November 2002. SN: 4150	Survey
National Centre for Social Research and University College London. Department of Epidemiology and Public Health, Health Survey for England, 1999 [computer file]. 3rd Edition. Colchester, Essex: UK Data Archive [distributor], February 2002. SN: 4365	Survey
National Centre for Social Research and University College London. Department of Epidemiology and Public Health, Health Survey for England, 2003 [computer file]. Colchester, Essex: UK Data Archive [distributor], March 2005. SN: 5098	Survey
National Centre for Social Research and University College London. Department of Epidemiology and Public Health, Health Survey for England, 2004 [computer file]. Colchester, Essex: UK Data Archive [distributor], July 2006. SN: 5439	Survey
NatCen Social Research and University College London. Department of Epidemiology and Public Health, Health Survey for England, 2012 [computer file]. Colchester, Essex: UK Data Archive [distributor], April 2014. SN: 7480, http://dx.doi.org/10.5255/UKDA-SN-7480-1	Survey
Department of Epidemiology and Public Health, University College London, National Centre for Social Research (NatCen). United Kingdom Health Survey for England 2009-2010 - HSCIC	Survey
NatCen Social Research and University College London. Department of Epidemiology and Public Health, Health Survey for England, 2013 [computer file]. Colchester, Essex: UK Data Archive [distributor], January 2015. SN: 7649, http://dx.doi.org/10.5255/UKDA-SN-7649-1	Survey
Forouhi NG, Luan J, Hennings S, Wareham NJ. Incidence of Type 2 diabetes in England and its association with baseline impaired fasting glucose: the Ely study 1990-2000. Diabet Med. 2007; 24(2): 200-7	Scientific literature
Wadsworth E, Shield J, Hunt L, Baum D. Insulin dependent diabetes in children under 5: incidence and ascertainment validation for 1992. BMJ. 1995; 310(6981): 700-3	Scientific literature
Whisman MA. Loneliness and the metabolic syndrome in a population-based sample of middle-aged and older adults. Health Psychol. 2010; 29(5): 550–4	Scientific literature

**Scotland**	
Wadsworth E, Shield J, Hunt L, Baum D. Insulin dependent diabetes in children under 5: incidence and ascertainment validation for 1992. BMJ. 1995; 310(6981): 700-3	Scientific literature
Joint Health Surveys Unit, University College London and Medical Research Council. Social and Public Health Sciences Unit, Scottish Health Survey, 2003 [computer file]. Colchester, Essex: UK Data Archive [distributor], February 2006. SN: 5318	Survey
Scottish Centre for Social Research and University College London. Department of Epidemiology and Public Health, Scottish Health Survey, 2008 [computer file]. 2nd Edition. Colchester, Essex: UK Data Archive [distributor], April 2013. SN: 6383, http://dx.doi.org/10.5255/UKDA-SN-6383-2	Survey
Schofield CJ, Yu N, Jain AS, Leese GP. Decreasing amputation rates in patients with diabetes-a population-based study. Diabet Med. 2009; 26(8): 773-7	Scientific literature
Department of Epidemiology and Public Health, University College London, MRC Social and Public Health Sciences Unit, University of Glasgow, Scottish Centre for Social Research (ScotCen). United Kingdom - Scottish Health Survey 2011 - Scottish Government	Survey
Department of Epidemiology and Public Health, University College London, MRC Social and Public Health Sciences Unit, University of Glasgow, Scottish Centre for Social Research (ScotCen). United Kingdom - Scottish Health Survey 2010 - Scottish Government	Survey
Department of Epidemiology and Public Health, University College London, MRC Social and Public Health Sciences Unit, University of Glasgow, Scottish Centre for Social Research (ScotCen). United Kingdom - Scottish Health Survey 2009 - Scottish Government	Survey
Kennon B, Leese GP, Cochrane L, Colhoun H, Wild S, Stang D, Sattar N, Pearson D, Lindsay RS, Morris AD, Livingstone S, Young M, McKnight J, Cunningham S. Reduced incidence of lower-extremity amputations in people with diabetes in Scotland: a nationwide study. Diabetes Care. 2012; 35(12): 2588-90	Scientific literature
Morris AD, McAlpine R, Steinke D, Boyle DI, Ebrahim AR, Vasudev N, Stewart CP, Jung RT, Leese GP, MacDonald TM, Newton RW. Diabetes and lower-limb amputations in the community. A retrospective cohort study. DARTS/MEMO Collaboration. Diabetes Audit and Research in Tayside Scotland/Medicines Monitoring Unit. Diabetes Care. 1998; 21(5): 738-43	Scientific literature
Crawford F, McCowan C, Dimitrov BD, Woodburn J, Wylie GH, Booth E, Leese GP, Bekker HL, Kleijnen J, Fahey T. The risk of foot ulceration in people with diabetes screened in community settings: findings from a cohort study. QJM. 2011; 104(5): 403-10	Scientific literature
Rangasami JJ, Greenwood DC, McSporran B, Smail PJ, Patterson CC, Waugh NR. Rising incidence of type 1 diabetes in Scottish children, 1984-93. The Scottish Study Group for the Care of Young Diabetics. Arch Dis Child. 1997; 77(3): 210-3	Scientific literature
**Wales**	
Wadsworth E, Shield J, Hunt L, Baum D. Insulin dependent diabetes in children under 5: incidence and ascertainment validation for 1992. BMJ. 1995; 310(6981): 700-3	Scientific literature
Davies M, Brophy S, Williams R, Taylor A. The prevalence, severity, and impact of painful diabetic peripheral neuropathy in type 2 diabetes. Diabetes Care. 2006; 29(7): 1518–22	Scientific literature
**Northern Ireland**	
Wadsworth E, Shield J, Hunt L, Baum D. Insulin dependent diabetes in children under 5: incidence and ascertainment validation for 1992. BMJ. 1995; 310(6981): 700-3	Scientific literature
Cardwell CR, Carson DJ, Patterson CC. Secular trends, disease maps and ecological analyses of the incidence of childhood onset Type 1 diabetes in Northern Ireland, 1989-2003. Diabet Med. 2007; 24(3): 289-95	Scientific literature
Patterson CC, Carson DJ, Hadden DR. Epidemiology of childhood IDDM in Northern Ireland 1989-1994: low incidence in areas with highest population density and most household crowding. Northern Ireland Diabetes Study Group. Diabetologia. 1996; 39(9): 1063-9	Scientific literature

Appendix Table 2: List of data sources used for chronic obstructive pulmonary disease (COPD) across UK countries

Citation	Data type
**United Kingdom**	
Swanney MP, Ruppel G, Enright PL, Pedersen OF, Crapo RO, Miller MR, Jensen RL, Falaschetti E, Schouten JP, Hankinson JL, Stocks J, Quanjer PH. Using the lower limit of normal for the FEV1/FVC ratio reduces the misclassification of airway obstruction. Thorax. 2008; 63(12): 1046-51	Scientific literature
**England**	
Swanney MP, Ruppel G, Enright PL, Pedersen OF, Crapo RO, Miller MR, Jensen RL, Falaschetti E, Schouten JP, Hankinson JL, Stocks J, Quanjer PH. Using the lower limit of normal for the FEV1/FVC ratio reduces the misclassification of airway obstruction. Thorax. 2008; 63(12): 1046-51	Scientific literature
**Scotland**	
Swanney MP, Ruppel G, Enright PL, Pedersen OF, Crapo RO, Miller MR, Jensen RL, Falaschetti E, Schouten JP, Hankinson JL, Stocks J, Quanjer PH. Using the lower limit of normal for the FEV1/FVC ratio reduces the misclassification of airway obstruction. Thorax. 2008; 63(12): 1046-51	Scientific literature
**Wales**	
Swanney MP, Ruppel G, Enright PL, Pedersen OF, Crapo RO, Miller MR, Jensen RL, Falaschetti E, Schouten JP, Hankinson JL, Stocks J, Quanjer PH. Using the lower limit of normal for the FEV1/FVC ratio reduces the misclassification of airway obstruction. Thorax. 2008; 63(12): 1046-51	Scientific literature
**Northern Ireland**	
Swanney MP, Ruppel G, Enright PL, Pedersen OF, Crapo RO, Miller MR, Jensen RL, Falaschetti E, Schouten JP, Hankinson JL, Stocks J, Quanjer PH. Using the lower limit of normal for the FEV1/FVC ratio reduces the misclassification of airway obstruction. Thorax. 2008; 63(12): 1046-51	Scientific literature

Appendix Table 3: List of data sources used for low back and neck pain across UK countries

Citation	Data type
**United Kingdom**	
World Health Organization (WHO). United Kingdom World Health Survey 2004. Geneva, Switzerland: World Health Organization (WHO), 2005	Survey
NatCen Social Research and University College London. Department of Epidemiology and Public Health, Health Survey for England, 2011 [computer file]. Colchester, Essex: UK Data Archive [distributor], April 2013. SN: 7260, http://dx.doi.org/10.5255/UKDA-SN-7260-1	Survey
Lacey RJ, Lewis M, Sim J. Presentation of pain drawings in questionnaire surveys: influence on prevalence of neck and upper limb pain in the community. Pain. 2003; 105(1-2): 293-301	Scientific literature
Palmer KT, Walker-Bone K, Griffin MJ, Syddall H, Pannett B, Coggon D, Cooper C. Prevalence and occupational associations of neck pain in the British population. Scand J Work Environ Health. 2001; 27(1): 49-56	Scientific literature
Sim J, Lacey RJ, Lewis M. The impact of workplace risk factors on the occurrence of neck and upper limb pain: a general population study. BMC Public Health. 2006; 6(1): 234	Scientific literature
Thomas E, Peat G, Harris L, Wilkie R, Croft PR. The prevalence of pain and pain interference in a general population of older adults: cross-sectional findings from the North Staffordshire Osteoarthritis Project (NorStOP). Pain. 2004; 110(1-2): 361-8	Scientific literature
Urwin M, Symmons D, Allison T, Brammah T, Busby H, Roxby M, Simmons A, Williams G. Estimating the burden of musculoskeletal disorders in the community: the comparative prevalence of symptoms at different anatomical sites, and the relation to social deprivation. Ann Rheum Dis. 1998; 57(11): 649-55	Scientific literature
Webb R, Brammah T, Lunt M, Urwin M, Allison T, Symmons D. Prevalence and predictors of intense, chronic, and disabling neck and back pain in the UK general population. Spine. 2003; 28(11): 1195-202	Scientific literature
Macfarlane GJ, Beasley M, Jones EA, Prescott GJ, Docking R, Keeley P, McBeth J, Jones GT. The prevalence and management of low back pain across adulthood: Results from a population-based cross-sectional study (the MUSICIAN study). Pain. 2012; 153(1): 27-32	Scientific literature
Docking RE, Fleming J, Brayne C, Zhao J, Macfarlane GJ, Jones GT, Cambridge City over-75s Cohort Study collaboration. Epidemiology of back pain in older adults: prevalence and risk factors for back pain onset. Rheumatology (Oxford). 2011; 50(9): 1645-53	Scientific literature
Department of Health (United Kingdom), Office for National Statistics (United Kingdom). The Prevalence of Back Pain in Great Britain in 1998. United Kingdom: Department of Health (United Kingdom), 1999	Report
Murphy S, Buckle P, Stubbs D. A cross-sectional study of self-reported back and neck pain among English schoolchildren and associated physical and psychological risk factors. Arch Dis Child. 2007; 38(6): 797-804	Scientific literature
Palmer KT, Walsh K, Bendall H, Cooper C, Coggon D. Back pain in Britain: comparison of two prevalence surveys at an interval of 10 years. BMJ. 2000; 320(7249): 1577-8	Scientific literature
Zhang L, Zhang W-H, Zhang L, Wang P-Y. Prevalence of overweight/obesity and its associations with hypertension, diabetes, dyslipidemia, and metabolic syndrome: a survey in the suburban area of Beijing, 2007. Obes Facts. 2011; 4(4): 284-9	Scientific literature
Croft PR, Rigby AS. Socioeconomic influences on back problems in the community in Britain. J Epidemiol Community Health. 1994; 48(2): 166-70	Scientific literature
Elliott AM, Smith BH, Penny KI, Smith WC, Chambers WA. The epidemiology of chronic pain in the community. Lancet. 1999; 354(9186): 1248-52	Scientific literature
Watson KD, Papageorgiou AC, Jones GT, Taylor S, Symmons DPM, Silman AJ, Macfarlane GJ. Low back pain in schoolchildren: occurrence and characteristics. Pain. 2002; 97(1-2): 87-92	Scientific literature
Harkness EF, Macfarlane GJ, Silman AJ, McBeth J. Is musculoskeletal pain more common now than 40 years ago?: Two population-based cross-sectional studies. Rheumatology (Oxford). 2005; 44(7): 890-5	Scientific literature
Papageorgiou AC, Croft PR, Ferry S, Jayson MI, Silman AJ. Estimating the prevalence of low back pain in the general population. Evidence from the South Manchester Back Pain Survey. Spine. 1995; 20(17): 1889-94	Scientific literature

Walsh K, Cruddas M, Coggon D. Low back pain in eight areas of Britain. J Epidemiol Community Health. 1992; 46(3): 227-30	Scientific literature
Wright D, Barrow S, Fisher AD, Horsley SD, Jayson MI. Influence of physical, psychological and behavioural factors on consultations for back pain. Br J Rheumatol. 1995; 34(2): 156-61	Scientific literature
World Health Organization Regional Office for Europe (EURO-WHO). Health Behaviour in School-aged Children: WHO Collaborative Cross-National survey/study (HBSC) 1998	Survey
World Health Organization Regional Office for Europe (EURO-WHO). Health Behaviour in School-aged Children: WHO Collaborative Cross-National survey/study (HBSC) 1998	Survey
World Health Organization Regional Office for Europe (EURO-WHO). Health Behaviour in School-aged Children: WHO Collaborative Cross-National survey/study (HBSC) 1998	Survey
World Health Organization Regional Office for Europe (EURO-WHO). Health Behaviour in School-aged Children: WHO Collaborative Cross-National survey/study (HBSC) 2002	Survey
World Health Organization Regional Office for Europe (EURO-WHO). Health Behaviour in School-aged Children: WHO Collaborative Cross-National survey/study (HBSC) 2006	Survey
World Health Organization Regional Office for Europe (EURO-WHO). Health Behaviour in School-aged Children: WHO Collaborative Cross-National survey/study (HBSC) 2009-2010	Survey
**England**	
Urwin M, Symmons D, Allison T, Brammah T, Busby H, Roxby M, Simmons A, Williams G. Estimating the burden of musculoskeletal disorders in the community: the comparative prevalence of symptoms at different anatomical sites, and the relation to social deprivation. Ann Rheum Dis. 1998; 57(11): 649-55	Scientific literature
Webb R, Brammah T, Lunt M, Urwin M, Allison T, Symmons D. Prevalence and predictors of intense, chronic, and disabling neck and back pain in the UK general population. Spine. 2003; 28(11): 1195-202	Scientific literature
Zhang L, Zhang W-H, Zhang L, Wang P-Y. Prevalence of overweight/obesity and its associations with hypertension, diabetes, dyslipidemia, and metabolic syndrome: a survey in the suburban area of Beijing, 2007. Obes Facts. 2011; 4(4): 284-9	Scientific literature
Watson KD, Papageorgiou AC, Jones GT, Taylor S, Symmons DPM, Silman AJ, Macfarlane GJ. Low back pain in schoolchildren: occurrence and characteristics. Pain. 2002; 97(1-2): 87-92	Scientific literature
Harkness EF, Macfarlane GJ, Silman AJ, McBeth J. Is musculoskeletal pain more common now than 40 years ago?: Two population-based cross-sectional studies. Rheumatology (Oxford). 2005; 44(7): 890-5	Scientific literature
Papageorgiou AC, Croft PR, Ferry S, Jayson MI, Silman AJ. Estimating the prevalence of low back pain in the general population. Evidence from the South Manchester Back Pain Survey. Spine. 1995; 20(17): 1889-94	Scientific literature
Wright D, Barrow S, Fisher AD, Horsley SD, Jayson MI. Influence of physical, psychological and behavioural factors on consultations for back pain. Br J Rheumatol. 1995; 34(2): 156-61	Scientific literature
Docking RE, Fleming J, Brayne C, Zhao J, Macfarlane GJ, Jones GT, Cambridge City over-75s Cohort Study collaboration. Epidemiology of back pain in older adults: prevalence and risk factors for back pain onset. Rheumatology (Oxford). 2011; 50(9): 1645-53	Scientific literature
Lacey RJ, Lewis M, Sim J. Presentation of pain drawings in questionnaire surveys: influence on prevalence of neck and upper limb pain in the community. Pain. 2003; 105(1-2): 293-301	Scientific literature
Sim J, Lacey RJ, Lewis M. The impact of workplace risk factors on the occurrence of neck and upper limb pain: a general population study. BMC Public Health. 2006; 6(1): 234	Scientific literature
Thomas E, Peat G, Harris L, Wilkie R, Croft PR. The prevalence of pain and pain interference in a general population of older adults: cross-sectional findings from the North Staffordshire Osteoarthritis Project (NorStOP). Pain. 2004; 110(1-2): 361-8	Scientific literature
Murphy S, Buckle P, Stubbs D. A cross-sectional study of self-reported back and neck pain among English schoolchildren and associated physical and psychological risk factors. Arch Dis Child. 2007; 38(6): 797-804	Scientific literature
World Health Organization (WHO). United Kingdom World Health Survey 2004. Geneva, Switzerland: World Health Organization (WHO), 2005	Survey
NatCen Social Research and University College London. Department of Epidemiology and Public Health, Health Survey for England, 2011 [computer file]. Colchester, Essex: UK Data Archive [distributor], April 2013. SN: 7260, http://dx.doi.org/10.5255/UKDA-SN-7260-1	Survey
Palmer KT, Walker-Bone K, Griffin MJ, Syddall H, Pannett B, Coggon D, Cooper C. Prevalence and occupational associations of neck pain in the British population. Scand J Work Environ Health. 2001; 27(1): 49-56	Scientific literature

World Health Organization Regional Office for Europe (EURO-WHO). Health Behaviour in School-aged Children: WHO Collaborative Cross-National survey/study (HBSC) 2002	Survey
World Health Organization Regional Office for Europe (EURO-WHO). Health Behaviour in School-aged Children: WHO Collaborative Cross-National survey/study (HBSC) 2006	Survey
World Health Organization Regional Office for Europe (EURO-WHO). Health Behaviour in School-aged Children: WHO Collaborative Cross-National survey/study (HBSC) 2009-2010	Survey
**Scotland**	
World Health Organization (WHO). United Kingdom World Health Survey 2004. Geneva, Switzerland: World Health Organization (WHO), 2005	Survey
Palmer KT, Walker-Bone K, Griffin MJ, Syddall H, Pannett B, Coggon D, Cooper C. Prevalence and occupational associations of neck pain in the British population. Scand J Work Environ Health. 2001; 27(1): 49-56	Scientific literature
World Health Organization Regional Office for Europe (EURO-WHO). Health Behaviour in School-aged Children: WHO Collaborative Cross-National survey/study (HBSC) 2002	Survey
World Health Organization Regional Office for Europe (EURO-WHO). Health Behaviour in School-aged Children: WHO Collaborative Cross-National survey/study (HBSC) 2006	Survey
World Health Organization Regional Office for Europe (EURO-WHO). Health Behaviour in School-aged Children: WHO Collaborative Cross-National survey/study (HBSC) 2009-2010	Survey
Macfarlane GJ, Beasley M, Jones EA, Prescott GJ, Docking R, Keeley P, McBeth J, Jones GT. The prevalence and management of low back pain across adulthood: Results from a population-based cross-sectional study (the MUSICIAN study). Pain. 2012; 153(1): 27-32	Scientific literature
Elliott AM, Smith BH, Penny KI, Smith WC, Chambers WA. The epidemiology of chronic pain in the community. Lancet. 1999; 354(9186): 1248-52	Scientific literature
World Health Organization Regional Office for Europe (EURO-WHO). Health Behaviour in School-aged Children: WHO Collaborative Cross-National survey/study (HBSC) 1998	Survey
**Wales**	
World Health Organization (WHO). United Kingdom World Health Survey 2004. Geneva, Switzerland: World Health Organization (WHO), 2005	Survey
Palmer KT, Walker-Bone K, Griffin MJ, Syddall H, Pannett B, Coggon D, Cooper C. Prevalence and occupational associations of neck pain in the British population. Scand J Work Environ Health. 2001; 27(1): 49-56	Scientific literature
World Health Organization Regional Office for Europe (EURO-WHO). Health Behaviour in School-aged Children: WHO Collaborative Cross-National survey/study (HBSC) 2002	Survey
World Health Organization Regional Office for Europe (EURO-WHO). Health Behaviour in School-aged Children: WHO Collaborative Cross-National survey/study (HBSC) 2006	Survey
World Health Organization Regional Office for Europe (EURO-WHO). Health Behaviour in School-aged Children: WHO Collaborative Cross-National survey/study (HBSC) 2009-2010	Survey
Macfarlane GJ, Beasley M, Jones EA, Prescott GJ, Docking R, Keeley P, McBeth J, Jones GT. The prevalence and management of low back pain across adulthood: Results from a population-based cross-sectional study (the MUSICIAN study). Pain. 2012; 153(1): 27-32	Scientific literature
Elliott AM, Smith BH, Penny KI, Smith WC, Chambers WA. The epidemiology of chronic pain in the community. Lancet. 1999; 354(9186): 1248-52	Scientific literature
World Health Organization Regional Office for Europe (EURO-WHO). Health Behaviour in School-aged Children: WHO Collaborative Cross-National survey/study (HBSC) 1998	Survey
**Northern Ireland**	
World Health Organization (WHO). United Kingdom World Health Survey 2004. Geneva, Switzerland: World Health Organization (WHO), 2005	Survey
World Health Organization Regional Office for Europe (EURO-WHO). Health Behaviour in School-aged Children: WHO Collaborative Cross-National survey/study (HBSC) 1998	Survey

Appendix Table 4: List of data sources used for skin conditions across UK countries

Citation	Data type
**United Kingdom**	
Rea JN, Newhouse ML, Halil T. Skin disease in Lambeth. A community study of prevalence and use of medical care. Br J Prev Soc Med. 1976; 30(2): 107-14	Scientific literature
Steele K. Primary dermatological care in general practice. J R Coll Gen Pract. 1984; 34(258): 22-3	Scientific literature
Williams H, Stewart A, Von Mutius E, Cookson W, Anderson HR. Is eczema really on the increase worldwide. J Allergy Clin Immunol. 2008; 121(4): 947-954	Scientific literature
Mallen CD, Mottram S, Wynne-Jones G, Thomas E. Birth-related exposures and asthma and allergy in adulthood: a population-based cross-sectional study of young adults in North Staffordshire. J Asthma. 2008; 45(4): 309-12	Scientific literature
Punekar YS, Sheikh A. Establishing the incidence and prevalence of clinician-diagnosed allergic conditions in children and adolescents using routinely collected data from general practices. Clin Exp Allergy. 2009; 39(8): 1209-16	Scientific literature
Schofield JK, Fleming D, Grindlay D, Williams H. Skin conditions are the commonest new reason people present to general practitioners in England and Wales. Br J Dermatol. 2011; 165(5): 1044-50	Scientific literature
Ziyab AH, Raza A, Karmaus W, Tongue N, Zhang H, Matthews S, Arshad SH, Roberts G. Trends in eczema in the first 18 years of life: results from the Isle of Wight 1989 birth cohort study. Clin Exp Allergy. 2010; 40(12): 1776-84	Scientific literature
Odhiambo JA, Williams HC, Clayton TO, Robertson CF, Asher MI, ISAAC Phase Three Study Group. Global variations in prevalence of eczema symptoms in children from ISAAC Phase Three. J Allergy Clin Immunol. 2009; 124(6): 1251-1258	Scientific literature
Flohr C, Weiland SK, Weinmayr G, Björkstén B, Bråbäck L, Brunekreef B, Büchele G, Clausen M, Cookson WOC, von Mutius E, Strachan DP, Williams HC, ISAAC Phase Two Study Group. The role of atopic sensitization in flexural eczema: findings from the International Study of Asthma and Allergies in Childhood Phase Two. J Allergy Clin Immunol. 2008; 121(1): 141-147	Scientific literature
Flohr C, Weinmayr G, Weiland SK, Addo-Yobo E, Annesi-Maesano I, Björkstén B, Bråbäck L, Büchele G, Chico M, Cooper P, Clausen M, El Sharif N, Martinez Gimeno A, Mathur RS, von Mutius E, Morales Suarez-Varela M, Pearce N, Svabe V, Wong GWK, Yu M, Zhong NS, Williams HC, ISAAC Phase Two Study Group. How well do questionnaires perform compared with physical examination in detecting flexural eczema? Findings from the International Study of Asthma and Allergies in Childhood (ISAAC) Phase Two. Br J Dermatol. 2009; 161(4): 846-53	Scientific literature
Anderson HR, Ruggles R, Strachan DP, Austin JB, Burr M, Jeffs D, Standring P, Steriu A, Goulding R. Trends in prevalence of symptoms of asthma, hay fever, and eczema in 12-14 year olds in the British Isles, 1995-2002: questionnaire survey. BMJ. 2004; 1052–3	Scientific literature
Devenny A. Respiratory symptoms and atopy in children in Aberdeen: questionnaire studies of a defined school population repeated over 35 years. BMJ. 2004; 329(7464): 489–90	Scientific literature
Kuehni CE, Davis A, Brooke AM, Silverman M. Are all wheezing disorders in very young (preschool) children increasing in prevalence?. Lancet. 2001; 357(9271): 1821–5	Scientific literature
Kwong GNM, Proctor A, Billings C, Duggan R, Das C, Whyte MKB, Powell CVE, Primhak R. Increasing prevalence of asthma diagnosis and symptoms in children is confined to mild symptoms. Thorax. 2001; 312–4	Scientific literature
McNeill G, Tagiyeva N, Aucott L, Russell G, Helms PJ. Changes in the prevalence of asthma, eczema and hay fever in pre-pubertal children: a 40-year perspective. Paediatr Perinat Epidemiol. 2009; 23(6): 506–12	Scientific literature
Shamssain M. Trends in the prevalence and severity of asthma, rhinitis and atopic eczema in 6- to 7- and 13- to 14-yr-old children from the north-east of England. Pediatr Allergy Immunol. 2007; 18(2): 149–53	Scientific literature
Garcia-Marcos L, Robertson CF, Ross Anderson H, Ellwood P, Williams HC, Wong GW. Does migration affect asthma, rhinoconjunctivitis and eczema prevalence? Global findings from the international study of asthma and allergies in childhood. Int J Epidemiol. 2014; 43(6): 1846-54	Scientific literature

Nagel G, Weinmayr G, Flohr C, Kleiner A, Strachan DP. Association of pertussis and measles infections and immunizations with asthma and allergic sensitization in ISAAC Phase Two. Pediatr Allergy Immunol. 2012; 23(8): 737-46	Scientific literature
Katebi R, Williams G, Bourke M, Harrison A, Verma A. What factors are associated with the prevalence of atopic symptoms amongst adolescents in Greater Manchester?. Eur J Public Health. 2015; nan	Scientific literature
Ziyab AH, Karmaus W, Zhang H, Holloway JW, Steck SE, Ewart S, Arshad SH. Allergic sensitization and filaggrin variants predispose to the comorbidity of eczema, asthma, and rhinitis: results from the Isle of Wight birth cohort. Clin Exp Allergy. 2014; 44(9): 1170-8	Scientific literature
Osman M, Tagiyeva N, Wassall HJ, Ninan TK, Devenny AM, McNeill G, Helms PJ, Russell G. Changing trends in sex specific prevalence rates for childhood asthma, eczema, and hay fever. Pediatr Pulmonol. 2007; 42(1): 60–5	Scientific literature
Nevitt GJ, Hutchinson PE. Psoriasis in the community: prevalence, severity and patients' beliefs and attitudes towards the disease. Br J Dermatol. 1996; 135(4): 533-7	Scientific literature
Huerta C, Rivero E, Rodríguez LAG. Incidence and risk factors for psoriasis in the general population. Arch Dermatol. 2007; 143(12): 1559-65	Scientific literature
Gelfand JM, Weinstein R, Porter SB, Neimann AL, Berlin JA, Margolis DJ. Prevalence and treatment of psoriasis in the United Kingdom: a population-based study. Arch Dermatol. 2005; 141(12): 1537-41	Scientific literature
Springate DA, Parisi R, Kontopantelis E, Reeves D, Griffiths CE, Ashcroft DM. Incidence, prevalence and mortality of patients with psoriasis: a UK population-based cohort study. Br J Dermatol. 2016; nan	Scientific literature
Gillard SE, Finlay AY. Current management of psoriasis in the United Kingdom: patterns of prescribing and resource use in primary care. Int J Clin Pract. 2005; 59(11): 1260–7	Scientific literature
Seminara NM, Abuabara K, Shin DB, Langan SM, Kimmel SE, Margolis D, Troxel AB, Gelfand JM. Validity of The Health Improvement Network (THIN) for the study of psoriasis. Br J Dermatol. 2011; 164(3): 602–9	Scientific literature
World Health Organization Regional Office for Europe (WHO/Europe). European Hospital Morbidity Database 1999-2007. Copenhagen, Denmark: World Health Organization Regional Office for Europe (WHO/Europe)	Administrative record
World Health Organization (WHO). United Kingdom World Health Survey 2004. Geneva, Switzerland: World Health Organization (WHO), 2005	Survey
Organization for Economic Co-operation and Development (OECD). OECD Health Statistics. Paris, France: Organization for Economic Co-operation and Development (OECD)	Administrative record
Trinity College Dublin. Ireland Longitudinal Study on Ageing 2009-2011. Dublin, Ireland: Irish Social Science Data Archive, University College Dublin	Survey
Trinity College Dublin. Ireland Longitudinal Study on Ageing 2012-2013. Dublin, Ireland: Irish Social Science Data Archive, University College Dublin	Survey
National Centre for Social Research (NatCen), World Health Organization (WHO). United Kingdom WHO Multi-country Survey Study on Health and Health System Responsiveness 2000-2001. Geneva, Switzerland: World Health Organization (WHO)	Survey
NHS England. United Kingdom - England Hospital Episode Statistics 2003-2007	Administrative record
NHS England. United Kingdom - England Hospital Episode Statistics 2001-2002	Administrative record
NHS England. United Kingdom - England Hospital Episode Statistics 2008-2012	Administrative record
NHS England. United Kingdom - England Hospital Episode Statistics 2013-2014	Administrative record
Department of Health, Social Services and Public Safety (Northern Ireland), Information Centre for Health and Social Care, NHS, NHS England, NHS Health Scotland, NHS Wales. United Kingdom Hospital Patient and Discharge Data 2006	Administrative record
Department of Health, Social Services and Public Safety (Northern Ireland), Information Centre for Health and Social Care, NHS, NHS England, NHS Health Scotland, NHS Wales. United Kingdom Hospital Patient and Discharge Data 2012	Administrative record
Department of Health, Social Services and Public Safety (Northern Ireland), Information Centre for Health and Social Care, NHS, NHS England, NHS Health Scotland, NHS Wales. United Kingdom Hospital Patient and Discharge Data 2013	Administrative record

Department of Health, Social Services and Public Safety (Northern Ireland), Information Centre for Health and Social Care, NHS, NHS England, NHS Health Scotland, NHS Wales. United Kingdom Hospital Patient and Discharge Data 2014	Administrative record
Pannell RS, Fleming DM, Cross KW. The incidence of molluscum contagiosum, scabies and lichen planus. Epidemiol Infect. 2005; 133(6): 985-91	Scientific literature
Konstantinou GN, Papadopoulos NG, Tavladaki T, Tsekoura T, Tsilimigaki A, Grattan CEH. Childhood acute urticaria in northern and southern Europe shows a similar epidemiological pattern and significant meteorological influences. Pediatr Allergy Immunol. 2011; 22(1 Pt 1): 36-42	Scientific literature
Margolis DJ, Bilker W, Knauss J, Baumgarten M, Strom BL. The incidence and prevalence of pressure ulcers among elderly patients in general medical practice. Ann Epidemiol. 2002; 12(5): 321-5	Scientific literature
Rea JN, Newhouse ML, Halil T. Skin disease in Lambeth. A community study of prevalence and use of medical care. Br J Prev Soc Med. 1976; 30(2): 107-14	Scientific literature
Steele K. Primary dermatological care in general practice. J R Coll Gen Pract. 1984; 34(258): 22-3	Scientific literature
Williams H, Stewart A, Von Mutius E, Cookson W, Anderson HR. Is eczema really on the increase worldwide. J Allergy Clin Immunol. 2008; 121(4): 947-954	Scientific literature
Mallen CD, Mottram S, Wynne-Jones G, Thomas E. Birth-related exposures and asthma and allergy in adulthood: a population-based cross-sectional study of young adults in North Staffordshire. J Asthma. 2008; 45(4): 309-12	Scientific literature
Punekar YS, Sheikh A. Establishing the incidence and prevalence of clinician-diagnosed allergic conditions in children and adolescents using routinely collected data from general practices. Clin Exp Allergy. 2009; 39(8): 1209-16	Scientific literature
Schofield JK, Fleming D, Grindlay D, Williams H. Skin conditions are the commonest new reason people present to general practitioners in England and Wales. Br J Dermatol. 2011; 165(5): 1044-50	Scientific literature
Ziyab AH, Raza A, Karmaus W, Tongue N, Zhang H, Matthews S, Arshad SH, Roberts G. Trends in eczema in the first 18 years of life: results from the Isle of Wight 1989 birth cohort study. Clin Exp Allergy. 2010; 40(12): 1776-84	Scientific literature
Odhiambo JA, Williams HC, Clayton TO, Robertson CF, Asher MI, ISAAC Phase Three Study Group. Global variations in prevalence of eczema symptoms in children from ISAAC Phase Three. J Allergy Clin Immunol. 2009; 124(6): 1251-1258	Scientific literature
Flohr C, Weiland SK, Weinmayr G, Björkstén B, Bråbäck L, Brunekreef B, Büchele G, Clausen M, Cookson WOC, von Mutius E, Strachan DP, Williams HC, ISAAC Phase Two Study Group. The role of atopic sensitization in flexural eczema: findings from the International Study of Asthma and Allergies in Childhood Phase Two. J Allergy Clin Immunol. 2008; 121(1): 141-147	Scientific literature
Flohr C, Weinmayr G, Weiland SK, Addo-Yobo E, Annesi-Maesano I, Björkstén B, Bråbäck L, Büchele G, Chico M, Cooper P, Clausen M, El Sharif N, Martinez Gimeno A, Mathur RS, von Mutius E, Morales Suarez-Varela M, Pearce N, Svabe V, Wong GWK, Yu M, Zhong NS, Williams HC, ISAAC Phase Two Study Group. How well do questionnaires perform compared with physical examination in detecting flexural eczema? Findings from the International Study of Asthma and Allergies in Childhood (ISAAC) Phase Two. Br J Dermatol. 2009; 161(4): 846-53	Scientific literature
Anderson HR, Ruggles R, Strachan DP, Austin JB, Burr M, Jeffs D, Standring P, Steriu A, Goulding R. Trends in prevalence of symptoms of asthma, hay fever, and eczema in 12-14 year olds in the British Isles, 1995-2002: questionnaire survey. BMJ. 2004; 1052–3	Scientific literature
Devenny A. Respiratory symptoms and atopy in children in Aberdeen: questionnaire studies of a defined school population repeated over 35 years. BMJ. 2004; 329(7464): 489–90	Scientific literature
Kuehni CE, Davis A, Brooke AM, Silverman M. Are all wheezing disorders in very young (preschool) children increasing in prevalence?. Lancet. 2001; 357(9271): 1821–5	Scientific literature
**England**	
Williams H, Stewart A, Von Mutius E, Cookson W, Anderson HR. Is eczema really on the increase worldwide. J Allergy Clin Immunol. 2008; 121(4): 947-954	Scientific literature
Odhiambo JA, Williams HC, Clayton TO, Robertson CF, Asher MI, ISAAC Phase Three Study Group. Global variations in prevalence of eczema symptoms in children from ISAAC Phase Three. J Allergy Clin Immunol. 2009; 124(6): 1251-1258	Scientific literature

Shamssain M. Trends in the prevalence and severity of asthma, rhinitis and atopic eczema in 6- to 7- and 13- to 14-yr-old children from the north-east of England. Pediatr Allergy Immunol. 2007; 18(2): 149–53	Scientific literature
Katebi R, Williams G, Bourke M, Harrison A, Verma A. What factors are associated with the prevalence of atopic symptoms amongst adolescents in Greater Manchester?. Eur J Public Health. 2015; nan	Scientific literature
Kwong GNM, Proctor A, Billings C, Duggan R, Das C, Whyte MKB, Powell CVE, Primhak R. Increasing prevalence of asthma diagnosis and symptoms in children is confined to mild symptoms. Thorax. 2001; 312–4	Scientific literature
Mallen CD, Mottram S, Wynne-Jones G, Thomas E. Birth-related exposures and asthma and allergy in adulthood: a population-based cross-sectional study of young adults in North Staffordshire. J Asthma. 2008; 45(4): 309-12	Scientific literature
Nevitt GJ, Hutchinson PE. Psoriasis in the community: prevalence, severity and patients' beliefs and attitudes towards the disease. Br J Dermatol. 1996; 135(4): 533-7	Scientific literature
Rea JN, Newhouse ML, Halil T. Skin disease in Lambeth. A community study of prevalence and use of medical care. Br J Prev Soc Med. 1976; 30(2): 107-14	Scientific literature
Nagel G, Weinmayr G, Flohr C, Kleiner A, Strachan DP. Association of pertussis and measles infections and immunizations with asthma and allergic sensitization in ISAAC Phase Two. Pediatr Allergy Immunol. 2012; 23(8): 737-46	Scientific literature
Konstantinou GN, Papadopoulos NG, Tavladaki T, Tsekoura T, Tsilimigaki A, Grattan CEH. Childhood acute urticaria in northern and southern Europe shows a similar epidemiological pattern and significant meteorological influences. Pediatr Allergy Immunol. 2011; 22(1 Pt 1): 36-42	Scientific literature
Ziyab AH, Raza A, Karmaus W, Tongue N, Zhang H, Matthews S, Arshad SH, Roberts G. Trends in eczema in the first 18 years of life: results from the Isle of Wight 1989 birth cohort study. Clin Exp Allergy. 2010; 40(12): 1776-84	Scientific literature
Flohr C, Weinmayr G, Weiland SK, Addo-Yobo E, Annesi-Maesano I, Björkstén B, Bråbäck L, Büchele G, Chico M, Cooper P, Clausen M, El Sharif N, Martinez Gimeno A, Mathur RS, von Mutius E, Morales Suarez-Varela M, Pearce N, Svabe V, Wong GWK, Yu M, Zhong NS, Williams HC, ISAAC Phase Two Study Group. How well do questionnaires perform compared with physical examination in detecting flexural eczema? Findings from the International Study of Asthma and Allergies in Childhood (ISAAC) Phase Two. Br J Dermatol. 2009; 161(4): 846-53	Scientific literature
Punekar YS, Sheikh A. Establishing the incidence and prevalence of clinician-diagnosed allergic conditions in children and adolescents using routinely collected data from general practices. Clin Exp Allergy. 2009; 39(8): 1209-16	Scientific literature
Schofield JK, Fleming D, Grindlay D, Williams H. Skin conditions are the commonest new reason people present to general practitioners in England and Wales. Br J Dermatol. 2011; 165(5): 1044-50	Scientific literature
Flohr C, Weiland SK, Weinmayr G, Björkstén B, Bråbäck L, Brunekreef B, Büchele G, Clausen M, Cookson WOC, von Mutius E, Strachan DP, Williams HC, ISAAC Phase Two Study Group. The role of atopic sensitization in flexural eczema: findings from the International Study of Asthma and Allergies in Childhood Phase Two. J Allergy Clin Immunol. 2008; 121(1): 141-147	Scientific literature
Anderson HR, Ruggles R, Strachan DP, Austin JB, Burr M, Jeffs D, Standring P, Steriu A, Goulding R. Trends in prevalence of symptoms of asthma, hay fever, and eczema in 12-14 year olds in the British Isles, 1995-2002: questionnaire survey. BMJ. 2004; 1052–3	Scientific literature
Kuehni CE, Davis A, Brooke AM, Silverman M. Are all wheezing disorders in very young (preschool) children increasing in prevalence?. Lancet. 2001; 357(9271): 1821–5	Scientific literature
Garcia-Marcos L, Robertson CF, Ross Anderson H, Ellwood P, Williams HC, Wong GW. Does migration affect asthma, rhinoconjunctivitis and eczema prevalence? Global findings from the international study of asthma and allergies in childhood. Int J Epidemiol. 2014; 43(6): 1846-54	Scientific literature
Ziyab AH, Karmaus W, Zhang H, Holloway JW, Steck SE, Ewart S, Arshad SH. Allergic sensitization and filaggrin variants predispose to the comorbidity of eczema, asthma, and rhinitis: results from the Isle of Wight birth cohort. Clin Exp Allergy. 2014; 44(9): 1170-8	Scientific literature
Huerta C, Rivero E, Rodríguez LAG. Incidence and risk factors for psoriasis in the general population. Arch Dermatol. 2007; 143(12): 1559-65	Scientific literature
Gelfand JM, Weinstein R, Porter SB, Neimann AL, Berlin JA, Margolis DJ. Prevalence and treatment of psoriasis in the United Kingdom: a population-based study. Arch Dermatol. 2005; 141(12): 1537-41	Scientific literature

Springate DA, Parisi R, Kontopantelis E, Reeves D, Griffiths CE, Ashcroft DM. Incidence, prevalence and mortality of patients with psoriasis: a UK population-based cohort study. Br J Dermatol. 2016; nan	Scientific literature
Gillard SE, Finlay AY. Current management of psoriasis in the United Kingdom: patterns of prescribing and resource use in primary care. Int J Clin Pract. 2005; 59(11): 1260–7	Scientific literature
Seminara NM, Abuabara K, Shin DB, Langan SM, Kimmel SE, Margolis D, Troxel AB, Gelfand JM. Validity of The Health Improvement Network (THIN) for the study of psoriasis. Br J Dermatol. 2011; 164(3): 602–9	Scientific literature
World Health Organization Regional Office for Europe (WHO/Europe). European Hospital Morbidity Database 1999-2007. Copenhagen, Denmark: World Health Organization Regional Office for Europe (WHO/Europe)	Survey
World Health Organization (WHO). United Kingdom World Health Survey 2004. Geneva, Switzerland: World Health Organization (WHO), 2005	
Organization for Economic Co-operation and Development (OECD). OECD Health Statistics. Paris, France: Organization for Economic Co-operation and Development (OECD)	Survey
National Centre for Social Research (NatCen), World Health Organization (WHO). United Kingdom WHO Multi-country Survey Study on Health and Health System Responsiveness 2000-2001. Geneva, Switzerland: World Health Organization (WHO)	Survey
NHS England. United Kingdom - England Hospital Episode Statistics 2003-2007	Administrative record
NHS England. United Kingdom - England Hospital Episode Statistics 2001-2002	Administrative record
NHS England. United Kingdom - England Hospital Episode Statistics 2008-2012	Administrative record
NHS England. United Kingdom - England Hospital Episode Statistics 2013-2014	Administrative record
Department of Health, Social Services and Public Safety (Northern Ireland), Information Centre for Health and Social Care, NHS, NHS England, NHS Health Scotland, NHS Wales. United Kingdom Hospital Patient and Discharge Data 2006	Administrative record
Department of Health, Social Services and Public Safety (Northern Ireland), Information Centre for Health and Social Care, NHS, NHS England, NHS Health Scotland, NHS Wales. United Kingdom Hospital Patient and Discharge Data 2012	Administrative record
Department of Health, Social Services and Public Safety (Northern Ireland), Information Centre for Health and Social Care, NHS, NHS England, NHS Health Scotland, NHS Wales. United Kingdom Hospital Patient and Discharge Data 2013	Administrative record
Department of Health, Social Services and Public Safety (Northern Ireland), Information Centre for Health and Social Care, NHS, NHS England, NHS Health Scotland, NHS Wales. United Kingdom Hospital Patient and Discharge Data 2014	Administrative record
Pannell RS, Fleming DM, Cross KW. The incidence of molluscum contagiosum, scabies and lichen planus. Epidemiol Infect. 2005; 133(6): 985-91	Scientific literature
Margolis DJ, Bilker W, Knauss J, Baumgarten M, Strom BL. The incidence and prevalence of pressure ulcers among elderly patients in general medical practice. Ann Epidemiol. 2002; 12(5): 321-5	Scientific literature
**Scotland**	
Williams H, Stewart A, Von Mutius E, Cookson W, Anderson HR. Is eczema really on the increase worldwide. J Allergy Clin Immunol. 2008; 121(4): 947-954	Scientific literature
Odhiambo JA, Williams HC, Clayton TO, Robertson CF, Asher MI, ISAAC Phase Three Study Group. Global variations in prevalence of eczema symptoms in children from ISAAC Phase Three. J Allergy Clin Immunol. 2009; 124(6): 1251-1258	Scientific literature
Anderson HR, Ruggles R, Strachan DP, Austin JB, Burr M, Jeffs D, Standring P, Steriu A, Goulding R. Trends in prevalence of symptoms of asthma, hay fever, and eczema in 12-14 year olds in the British Isles, 1995-2002: questionnaire survey. BMJ. 2004; 1052–3	Scientific literature
Devenny A. Respiratory symptoms and atopy in children in Aberdeen: questionnaire studies of a defined school population repeated over 35 years. BMJ. 2004; 329(7464): 489–90	Scientific literature
McNeill G, Tagiyeva N, Aucott L, Russell G, Helms PJ. Changes in the prevalence of asthma, eczema and hay fever in pre-pubertal children: a 40-year perspective. Paediatr Perinat Epidemiol. 2009; 23(6): 506–12	Scientific literature
Garcia-Marcos L, Robertson CF, Ross Anderson H, Ellwood P, Williams HC, Wong GW. Does migration affect asthma, rhinoconjunctivitis and eczema prevalence? Global findings from the international study of asthma and allergies in childhood. Int J Epidemiol. 2014; 43(6): 1846-54	Scientific literature
Osman M, Tagiyeva N, Wassall HJ, Ninan TK, Devenny AM, McNeill G, Helms PJ, Russell G. Changing trends in sex specific prevalence rates for childhood asthma, eczema, and hay fever. Pediatr Pulmonol. 2007; 42(1): 60–5	Scientific literature

Springate DA, Parisi R, Kontopantelis E, Reeves D, Griffiths CE, Ashcroft DM. Incidence, prevalence and mortality of patients with psoriasis: a UK population-based cohort study. Br J Dermatol. 2016; nan	Scientific literature
Seminara NM, Abuabara K, Shin DB, Langan SM, Kimmel SE, Margolis D, Troxel AB, Gelfand JM. Validity of The Health Improvement Network (THIN) for the study of psoriasis. Br J Dermatol. 2011; 164(3): 602–9	Scientific literature
World Health Organization Regional Office for Europe (WHO/Europe). European Hospital Morbidity Database 1999-2007. Copenhagen, Denmark: World Health Organization Regional Office for Europe (WHO/Europe)	Administrative record
Trinity College Dublin. Ireland Longitudinal Study on Ageing 2012-2013. Dublin, Ireland: Irish Social Science Data Archive, University College Dublin	Survey
Department of Health, Social Services and Public Safety (Northern Ireland), Information Centre for Health and Social Care, NHS, NHS England, NHS Health Scotland, NHS Wales. United Kingdom Hospital Patient and Discharge Data 2006	Administrative record
Department of Health, Social Services and Public Safety (Northern Ireland), Information Centre for Health and Social Care, NHS, NHS England, NHS Health Scotland, NHS Wales. United Kingdom Hospital Patient and Discharge Data 2012	Administrative record
Department of Health, Social Services and Public Safety (Northern Ireland), Information Centre for Health and Social Care, NHS, NHS England, NHS Health Scotland, NHS Wales. United Kingdom Hospital Patient and Discharge Data 2013	Administrative record
Department of Health, Social Services and Public Safety (Northern Ireland), Information Centre for Health and Social Care, NHS, NHS England, NHS Health Scotland, NHS Wales. United Kingdom Hospital Patient and Discharge Data 2014	Administrative record
**Wales**	
World Health Organization Regional Office for Europe (WHO/Europe). European Hospital Morbidity Database 1999-2007. Copenhagen, Denmark: World Health Organization Regional Office for Europe (WHO/Europe)	Administrative record
Williams H, Stewart A, Von Mutius E, Cookson W, Anderson HR. Is eczema really on the increase worldwide. J Allergy Clin Immunol. 2008; 121(4): 947-954	Scientific literature
Pannell RS, Fleming DM, Cross KW. The incidence of molluscum contagiosum, scabies and lichen planus. Epidemiol Infect. 2005; 133(6): 985-91	Scientific literature
Punekar YS, Sheikh A. Establishing the incidence and prevalence of clinician-diagnosed allergic conditions in children and adolescents using routinely collected data from general practices. Clin Exp Allergy. 2009; 39(8): 1209-16	Scientific literature
Schofield JK, Fleming D, Grindlay D, Williams H. Skin conditions are the commonest new reason people present to general practitioners in England and Wales. Br J Dermatol. 2011; 165(5): 1044-50	Scientific literature
Odhiambo JA, Williams HC, Clayton TO, Robertson CF, Asher MI, ISAAC Phase Three Study Group. Global variations in prevalence of eczema symptoms in children from ISAAC Phase Three. J Allergy Clin Immunol. 2009; 124(6): 1251-1258	Scientific literature
Huerta C, Rivero E, Rodríguez LAG. Incidence and risk factors for psoriasis in the general population. Arch Dermatol. 2007; 143(12): 1559-65	Scientific literature
Margolis DJ, Bilker W, Knauss J, Baumgarten M, Strom BL. The incidence and prevalence of pressure ulcers among elderly patients in general medical practice. Ann Epidemiol. 2002; 12(5): 321-5	Scientific literature
Gelfand JM, Weinstein R, Porter SB, Neimann AL, Berlin JA, Margolis DJ. Prevalence and treatment of psoriasis in the United Kingdom: a population-based study. Arch Dermatol. 2005; 141(12): 1537-41	Scientific literature
Anderson HR, Ruggles R, Strachan DP, Austin JB, Burr M, Jeffs D, Standring P, Steriu A, Goulding R. Trends in prevalence of symptoms of asthma, hay fever, and eczema in 12-14 year olds in the British Isles, 1995-2002: questionnaire survey. BMJ. 2004; 1052–3	Scientific literature
Springate DA, Parisi R, Kontopantelis E, Reeves D, Griffiths CE, Ashcroft DM. Incidence, prevalence and mortality of patients with psoriasis: a UK population-based cohort study. Br J Dermatol. 2016; nan	Scientific literature
Gillard SE, Finlay AY. Current management of psoriasis in the United Kingdom: patterns of prescribing and resource use in primary care. Int J Clin Pract. 2005; 59(11): 1260–7	Scientific literature

Garcia-Marcos L, Robertson CF, Ross Anderson H, Ellwood P, Williams HC, Wong GW. Does migration affect asthma, rhinoconjunctivitis and eczema prevalence? Global findings from the international study of asthma and allergies in childhood. Int J Epidemiol. 2014; 43(6): 1846-54	Scientific literature
Seminara NM, Abuabara K, Shin DB, Langan SM, Kimmel SE, Margolis D, Troxel AB, Gelfand JM. Validity of The Health Improvement Network (THIN) for the study of psoriasis. Br J Dermatol. 2011; 164(3): 602–9	Scientific literature
Department of Health, Social Services and Public Safety (Northern Ireland), Information Centre for Health and Social Care, NHS, NHS England, NHS Health Scotland, NHS Wales. United Kingdom Hospital Patient and Discharge Data 2006	Administrative record
Department of Health, Social Services and Public Safety (Northern Ireland), Information Centre for Health and Social Care, NHS, NHS England, NHS Health Scotland, NHS Wales. United Kingdom Hospital Patient and Discharge Data 2012	Administrative record
Department of Health, Social Services and Public Safety (Northern Ireland), Information Centre for Health and Social Care, NHS, NHS England, NHS Health Scotland, NHS Wales. United Kingdom Hospital Patient and Discharge Data 2013	Administrative record
Department of Health, Social Services and Public Safety (Northern Ireland), Information Centre for Health and Social Care, NHS, NHS England, NHS Health Scotland, NHS Wales. United Kingdom Hospital Patient and Discharge Data 2014	Administrative record
**Northern Ireland**	
World Health Organization Regional Office for Europe (WHO/Europe). European Hospital Morbidity Database 1999-2007. Copenhagen, Denmark: World Health Organization Regional Office for Europe (WHO/Europe)	Administrative record
Department of Health, Social Services and Public Safety (Northern Ireland), Information Centre for Health and Social Care, NHS, NHS England, NHS Health Scotland, NHS Wales. United Kingdom Hospital Patient and Discharge Data 2006	Administrative record
Department of Health, Social Services and Public Safety (Northern Ireland), Information Centre for Health and Social Care, NHS, NHS England, NHS Health Scotland, NHS Wales. United Kingdom Hospital Patient and Discharge Data 2012	Administrative record
Department of Health, Social Services and Public Safety (Northern Ireland), Information Centre for Health and Social Care, NHS, NHS England, NHS Health Scotland, NHS Wales. United Kingdom Hospital Patient and Discharge Data 2013	Administrative record
Department of Health, Social Services and Public Safety (Northern Ireland), Information Centre for Health and Social Care, NHS, NHS England, NHS Health Scotland, NHS Wales. United Kingdom Hospital Patient and Discharge Data 2014	Administrative record
Seminara NM, Abuabara K, Shin DB, Langan SM, Kimmel SE, Margolis D, Troxel AB, Gelfand JM. Validity of The Health Improvement Network (THIN) for the study of psoriasis. Br J Dermatol. 2011; 164(3): 602–9	Scientific literature
Garcia-Marcos L, Robertson CF, Ross Anderson H, Ellwood P, Williams HC, Wong GW. Does migration affect asthma, rhinoconjunctivitis and eczema prevalence? Global findings from the international study of asthma and allergies in childhood. Int J Epidemiol. 2014; 43(6): 1846-54	Scientific literature
Springate DA, Parisi R, Kontopantelis E, Reeves D, Griffiths CE, Ashcroft DM. Incidence, prevalence and mortality of patients with psoriasis: a UK population-based cohort study. Br J Dermatol. 2016; nan	Scientific literature
Steele K. Primary dermatological care in general practice. J R Coll Gen Pract. 1984; 34(258): 22-3	Scientific literature
Trinity College Dublin. Ireland Longitudinal Study on Ageing 2009-2011. Dublin, Ireland: Irish Social Science Data Archive, University College Dublin	Survey

Appendix Table 5: List of data sources used for depressive disorders across UK countries

Citation	Data type
**United Kingdom**	
World Health Organization (WHO). United Kingdom World Health Survey 2004. Geneva, Switzerland: World Health Organization (WHO), 2005	Survey
West P, Sweeting H, Der G, Barton J, Lucas C. Voice-DISC identified DSM-IV disorders among 15-year-olds in the west of Scotland. J Am Acad Child Adolesc Psychiatry. 2003; 42(8): 941-9	Scientific literature
Ayuso-Mateos JL, Vázquez-Barquero JL, Dowrick C, Lehtinen V, Dalgard OS, Casey P, Wilkinson C, Lasa L, Page H, Dunn G, Wilkinson G. Depressive disorders in Europe: prevalence figures from the ODIN study. Br J Psychiatry. 2001; 179(4): 308-16	Scientific literature
Office of Population Censuses and Surveys. Social Survey Division, OPCS Surveys of Psychiatric Morbidity : Private Household Survey, 1993 [computer file]. Colchester, Essex: UK Data Archive [distributor], September 1996. SN: 3560, http://dx.doi.org/10.5255/UKDA-SN-3560-1	Survey
Office for National Statistics, Psychiatric Morbidity among Adults Living in Private Households, 2000 [computer file]. Colchester, Essex: UK Data Archive [distributor], May 2003. SN: 4653, http://dx.doi.org/10.5255/UKDA-SN-4653-1	Survey
Bunting B, Murphy S, O'Neill S, Ferry F. Prevalence and treatment of 12-month DSM-IV disorders in the Northern Ireland study of health and stress. Soc Psychiatry Psychiatr Epidemiol. 2013; 48(1): 81-93	Scientific literature
Adamson JA, Price GM, Breeze E, Bulpitt CJ, Fletcher AE. Are older people dying of depression? Findings from the Medical Research Council trial of the assessment and management of older people in the community. J Am Geriatr Soc. 2005; 53(7): 1128-32	Scientific literature
Copeland JR, Beekman AT, Dewey ME, Hooijer C, Jordan A, Lawlor BA, Lobo A, Magnusson H, Mann AH, Meller I, Prince MJ, Reischies F, Turrina C, deVries MW, Wilson KC. Depression in Europe. Geographical distribution among older people. Br J Psychiatry. 1999; 174: 312-21	Scientific literature
Donnelly M. Depression among adolescents in Northern Ireland. Adolescence. 1995; 30(118): 339-50	Scientific literature
Lépine JP, Gastpar M, Mendlewicz J, Tylee A. Depression in the community: the first pan-European study DEPRES (Depression Research in European Society). Int Clin Psychopharmacol. 1997; 12(1): 19-29	Scientific literature
Meltzer H, Gatward R, Goodman R, Ford T. Mental health of children and adolescents in Great Britain. Int Rev Psychiatry. 2003; 15(1-2): 185-7	Scientific literature
Saunders PA, Copeland JR, Dewey ME, Gilmore C, Larkin BA, Phaterpekar H, Scott A. The prevalence of dementia, depression and neurosis in later life: the Liverpool MRC-ALPHA Study. Int J Epidemiol. 1993; 22(5): 838-47	Scientific literature
Office for National Statistics. Social and Vital Statistics Division et al. , Mental Health of Children and Young People in Great Britain, 2004 [computer file]. Colchester, Essex: UK Data Archive [distributor], October 2005. SN: 5269, http://dx.doi.org/10.5255/UKDA-SN-5269-1	Administrative data
National Centre for Social Research and University of Leicester, Adult Psychiatric Morbidity Survey, 2007 [computer file]. 3rd Edition. Colchester, Essex: UK Data Archive [distributor], January 2011. SN: 6379, http://dx.doi.org/10.5255/UKDA-SN-6379-1	Survey
Jenkins R, Lewis G, Bebbington P, Brugha T, Farrell M, Gill B, Meltzer H. The National Psychiatric Morbidity surveys of Great Britain-initial findings from the household survey. Psychol Med. 1997; 27(4): 775-89	Scientific literature
**England**	
World Health Organization (WHO). United Kingdom World Health Survey 2004. Geneva, Switzerland: World Health Organization (WHO), 2005	Survey
Ayuso-Mateos JL, Vázquez-Barquero JL, Dowrick C, Lehtinen V, Dalgard OS, Casey P, Wilkinson C, Lasa L, Page H, Dunn G, Wilkinson G. Depressive disorders in Europe: prevalence figures from the ODIN study. Br J Psychiatry. 2001; 179(4): 308-16	Scientific literature
Office of Population Censuses and Surveys. Social Survey Division, OPCS Surveys of Psychiatric Morbidity : Private Household Survey, 1993 [computer file]. Colchester, Essex: UK Data Archive [distributor], September 1996. SN: 3560, http://dx.doi.org/10.5255/UKDA-SN-3560-1	Survey

Office for National Statistics, Psychiatric Morbidity among Adults Living in Private Households, 2000 [computer file]. Colchester, Essex: UK Data Archive [distributor], May 2003. SN: 4653, http://dx.doi.org/10.5255/UKDA-SN-4653-1	Administrative data
Adamson JA, Price GM, Breeze E, Bulpitt CJ, Fletcher AE. Are older people dying of depression? Findings from the Medical Research Council trial of the assessment and management of older people in the community. J Am Geriatr Soc. 2005; 53(7): 1128-32	Scientific literature
Copeland JR, Beekman AT, Dewey ME, Hooijer C, Jordan A, Lawlor BA, Lobo A, Magnusson H, Mann AH, Meller I, Prince MJ, Reischies F, Turrina C, deVries MW, Wilson KC. Depression in Europe. Geographical distribution among older people. Br J Psychiatry. 1999; 174: 312-21	Scientific literature
Lépine JP, Gastpar M, Mendlewicz J, Tylee A. Depression in the community: the first pan-European study DEPRES (Depression Research in European Society). Int Clin Psychopharmacol. 1997; 12(1): 19-29	Scientific literature
Meltzer H, Gatward R, Goodman R, Ford T. Mental health of children and adolescents in Great Britain. Int Rev Psychiatry. 2003; 15(1-2): 185-7	Scientific literature
Saunders PA, Copeland JR, Dewey ME, Gilmore C, Larkin BA, Phaterpekar H, Scott A. The prevalence of dementia, depression and neurosis in later life: the Liverpool MRC-ALPHA Study. Int J Epidemiol. 1993; 22(5): 838-47	Scientific literature
Office for National Statistics. Social and Vital Statistics Division et al. , Mental Health of Children and Young People in Great Britain, 2004 [computer file]. Colchester, Essex: UK Data Archive [distributor], October 2005. SN: 5269, http://dx.doi.org/10.5255/UKDA-SN-5269-1	Administrative data
National Centre for Social Research and University of Leicester, Adult Psychiatric Morbidity Survey, 2007 [computer file]. 3rd Edition. Colchester, Essex: UK Data Archive [distributor], January 2011. SN: 6379, http://dx.doi.org/10.5255/UKDA-SN-6379-1	Survey
**Scotland**	
World Health Organization (WHO). United Kingdom World Health Survey 2004. Geneva, Switzerland: World Health Organization (WHO), 2005	Survey
Adamson JA, Price GM, Breeze E, Bulpitt CJ, Fletcher AE. Are older people dying of depression? Findings from the Medical Research Council trial of the assessment and management of older people in the community. J Am Geriatr Soc. 2005; 53(7): 1128-32	Scientific literature
Meltzer H, Gatward R, Goodman R, Ford T. Mental health of children and adolescents in Great Britain. Int Rev Psychiatry. 2003; 15(1-2): 185-7	Scientific literature
Lépine JP, Gastpar M, Mendlewicz J, Tylee A. Depression in the community: the first pan-European study DEPRES (Depression Research in European Society). Int Clin Psychopharmacol. 1997; 12(1): 19-29	Scientific literature
Office for National Statistics. Social and Vital Statistics Division et al. , Mental Health of Children and Young People in Great Britain, 2004 [computer file]. Colchester, Essex: UK Data Archive [distributor], October 2005. SN: 5269, http://dx.doi.org/10.5255/UKDA-SN-5269-1	Survey
Office for National Statistics, Psychiatric Morbidity among Adults Living in Private Households, 2000 [computer file]. Colchester, Essex: UK Data Archive [distributor], May 2003. SN: 4653, http://dx.doi.org/10.5255/UKDA-SN-4653-1	Survey
Jenkins R, Lewis G, Bebbington P, Brugha T, Farrell M, Gill B, Meltzer H. The National Psychiatric Morbidity surveys of Great Britain-initial findings from the household survey. Psychol Med. 1997; 27(4): 775-89	Scientific literature
West P, Sweeting H, Der G, Barton J, Lucas C. Voice-DISC identified DSM-IV disorders among 15-year-olds in the west of Scotland. J Am Acad Child Adolesc Psychiatry. 2003; 42(8): 941-9	Scientific literature
**Wales**	
World Health Organization (WHO). United Kingdom World Health Survey 2004. Geneva, Switzerland: World Health Organization (WHO), 2005	Survey
Adamson JA, Price GM, Breeze E, Bulpitt CJ, Fletcher AE. Are older people dying of depression? Findings from the Medical Research Council trial of the assessment and management of older people in the community. J Am Geriatr Soc. 2005; 53(7): 1128-32	Scientific literature
Meltzer H, Gatward R, Goodman R, Ford T. Mental health of children and adolescents in Great Britain. Int Rev Psychiatry. 2003; 15(1-2): 185-7	Scientific literature
Lépine JP, Gastpar M, Mendlewicz J, Tylee A. Depression in the community: the first pan-European study DEPRES (Depression Research in European Society). Int Clin Psychopharmacol. 1997; 12(1): 19-29	Scientific literature

Office for National Statistics. Social and Vital Statistics Division et al. , Mental Health of Children and Young People in Great Britain, 2004 [computer file]. Colchester, Essex: UK Data Archive [distributor], October 2005. SN: 5269, http://dx.doi.org/10.5255/UKDA-SN-5269-1	Survey
Office for National Statistics, Psychiatric Morbidity among Adults Living in Private Households, 2000 [computer file]. Colchester, Essex: UK Data Archive [distributor], May 2003. SN: 4653, http://dx.doi.org/10.5255/UKDA-SN-4653-1	Survey
Jenkins R, Lewis G, Bebbington P, Brugha T, Farrell M, Gill B, Meltzer H. The National Psychiatric Morbidity surveys of Great Britain-initial findings from the household survey. Psychol Med. 1997; 27(4): 775-89	Scientific literature
Ayuso-Mateos JL, Vázquez-Barquero JL, Dowrick C, Lehtinen V, Dalgard OS, Casey P, Wilkinson C, Lasa L, Page H, Dunn G, Wilkinson G. Depressive disorders in Europe: prevalence figures from the ODIN study. Br J Psychiatry. 2001; 179(4): 308-16	Scientific literature
**Northern Ireland**	
World Health Organization (WHO). United Kingdom World Health Survey 2004. Geneva, Switzerland: World Health Organization (WHO), 2005	Survey
Adamson JA, Price GM, Breeze E, Bulpitt CJ, Fletcher AE. Are older people dying of depression? Findings from the Medical Research Council trial of the assessment and management of older people in the community. J Am Geriatr Soc. 2005; 53(7): 1128-32	Scientific literature
Meltzer H, Gatward R, Goodman R, Ford T. Mental health of children and adolescents in Great Britain. Int Rev Psychiatry. 2003; 15(1-2): 185-7	Scientific literature
Lépine JP, Gastpar M, Mendlewicz J, Tylee A. Depression in the community: the first pan-European study DEPRES (Depression Research in European Society). Int Clin Psychopharmacol. 1997; 12(1): 19-29	Scientific literature
Office for National Statistics. Social and Vital Statistics Division et al. , Mental Health of Children and Young People in Great Britain, 2004 [computer file]. Colchester, Essex: UK Data Archive [distributor], October 2005. SN: 5269, http://dx.doi.org/10.5255/UKDA-SN-5269-1	Survey
Jenkins R, Lewis G, Bebbington P, Brugha T, Farrell M, Gill B, Meltzer H. The National Psychiatric Morbidity surveys of Great Britain-initial findings from the household survey. Psychol Med. 1997; 27(4): 775-89	Scientific literature
Bunting B, Murphy S, O'Neill S, Ferry F. Prevalence and treatment of 12-month DSM-IV disorders in the Northern Ireland study of health and stress. Soc Psychiatry Psychiatr Epidemiol. 2013; 48(1): 81-93	Scientific literature
Donnelly M. Depression among adolescents in Northern Ireland. Adolescence. 1995; 30(118): 339-50	Scientific literature

Appendix Table 6: Correlation coefficients between Upper Tier Local Authority estimates and Index of Multiple Deprivation (IMD) scores for the 20 causes with the highest national burden for years of life lost (YLLs)in England, 2016

**20 causes with the highest burden for YLLs**		
**Cause**	**Correlation coefficient (r)**	**P value**
All causes	0.82	<0.001
Ischaemic heart disease	0.75	<0.001
Trachea, bronchus, and lung cancer	0.81	<0.001
Cerebrovascular disease	0.60	<0.001
Alzheimer's disease and other dementias	NA	
Chronic obstructive pulmonary disease	0.81	<0.001
Lower respiratory infections	0.68	<0.001
Colon and rectum cancer	0.59	<0.001
Cirrhosis and other chronic liver diseases	0.75	<0.001
Breast cancer	NA	
Self-harm	NA	
Other cardiovascular and circulatory diseases	0.68	<0.001
Pancreatic cancer	0.73	<0.001
Prostate cancer	0.33	<0.001
Oesophageal cancer	0.57	<0.001
Other neoplasms	0.52	<0.001
Stomach cancer	0.79	<0.001
Leukaemia	0.66	<0.001
Neonatal preterm birth complications	NA	
Congenital birth defects	NA	
Brain and nervous system cancer	0.06	0.457

NA = Not applicable because assumptions of Pearson's correlation coefficient not met.

Appendix Figure 1. Age-standardised disability-adjusted life years (DALYs) rate per 100,000 population, for the 20 causes with the highest DALY burden and decreasing Upper Tier Local Authority (UTLA) deprivation (Index of Multiple Deprivation [IMD]), England, both sexes, 2016

Upper Tier Local Authority	IMD score	All causes	Low back and neck pain	Ischaemic heart disease	Skin and subcutaneous diseases	Migraine	Sense organ diseases	Depressive disorders	Trachea, bronchus, and lung cancer	Cerebrovascular disease	Chronic obstructive pulmonary disease	Falls	Drug use disorders	Alzheimer's disease and other dementias	Anxiety disorders	Asthma	Oral disorders	Other musculoskeletal disorders	Congenital birth defects	Neonatal preterm birth	Lower respiratory infections	Self-harm
England (95% uncertainty interval)	NA	19995 (17149 - 23222)	1820 (1277 - 2387)	1139 (1099 - 1184)	1068 (730 - 1505)	719 (463 - 1007)	667 (462 - 922)	664 (454 - 910)	633 (618 - 652)	570 (524 - 611)	507 (479 - 548)	450 (330 - 597)	443 (363 - 523)	440 (371 - 526)	435 (304 - 591)	375 (255 - 526)	355 (217 - 551)	352 (245 - 490)	350 (313 - 398)	349 (311 - 390)	339 (317 - 358)	335 (287 - 367)
Blackpool	42	25574	1820	1811	1053	718	695	661	1025	913	837	671	643	455	436	416	356	321	379	451	573	676
Knowsley	41	22199	1835	1481	1066	732	666	668	1129	652	871	523	490	450	442	379	354	320	370	311	485	264
Kingston upon Hull, City of	41	22575	1819	1551	1060	714	674	661	1103	751	853	482	426	448	434	398	356	332	338	340	453	454
Liverpool	41	22740	1822	1371	1069	716	660	657	1113	670	813	555	817	451	346	376	353	338	382	415	514	312
Manchester	41	23009	1816	1585	1083	711	634	659	1065	754	867	593	708	448	433	384	351	317	355	430	471	326
Middlesbrough	40	22835	1825	1503	1050	720	679	663	1088	778	789	493	418	450	436	387	359	381	342	346	498	424
Birmingham	38	21511	1823	1348	1062	720	667	664	701	634	586	552	625	442	437	376	354	323	445	664	394	289
Nottingham	37	22494	1819	1438	1071	711	766	661	892	682	749	492	405	447	433	383	356	338	474	511	423	374
Tower Hamlets	36	20784	1795	1283	1096	704	592	657	866	546	690	447	679	428	430	416	347	368	330	361	334	234
Hackney	35	20719	1806	1226	1115	718	584	663	720	529	565	448	651	426	436	421	346	398	279	425	330	246
Barking and Dagenham	35	21800	1816	1458	1142	725	687	666	959	578	742	439	316	442	438	403	355	350	382	338	474	298
Sandwell	35	22017	1821	1444	1061	719	685	664	801	705	671	498	427	450	436	393	356	338	470	516	410	340
Stoke-on-Trent	34	23023	1817	1432	1051	715	677	661	930	626	774	663	605	452	435	383	354	370	575	617	416	365
Blackburn with Darwen	34	22603	1822	1723	1041	718	685	662	817	744	789	490	599	453	435	396	358	383	350	444	439	446
Rochdale	34	22319	1825	1601	1053	722	695	664	863	766	747	473	540	455	437	392	356	344	353	363	443	397
Wolverhampton	33	22293	1820	1455	1061	717	678	663	778	721	618	461	466	448	436	380	352	420	409	513	421	378
Hartlepool	33	22019	1829	1395	1053	725	691	665	1026	735	671	452	423	448	438	383	352	326	297	363	429	393
Bradford	33	21477	1825	1443	1047	719	685	663	739	697	694	448	519	449	437	373	359	395	452	504	418	345
Leicester	33	21743	1826	1598	1065	718	670	663	698	683	625	507	420	450	436	380	365	310	442	487	473	344
Salford	33	22671	1817	1526	1065	713	658	660	1020	711	811	527	538	450	434	388	354	327	383	362	485	381
Newham	33	20398	1801	1354	1058	704	673	659	716	552	628	428	355	434	431	413	352	329	315	346	400	269
Islington	33	20610	1806	1172	1103	715	597	663	808	509	583	466	662	430	435	407	348	375	310	257	370	280
Halton	32	21766	1826	1418	1072	723	656	665	933	657	694	489	388	451	438	386	357	336	357	327	478	375
Haringey	31	19959	1810	1165	1074	715	669	663	603	527	478	414	448	432	435	408	355	354	277	324	360	326
North East Lincolnshire	31	21567	1826	1489	1056	720	667	664	785	663	663	419	359	447	437	378	362	357	386	340	385	457
South Tyneside	31	21342	1830	1264	1102	724	698	665	1010	668	690	462	510	449	438	371	355	337	286	229	376	364
Walsall	30	21876	1823	1462	1052	720	692	664	726	627	636	493	538	446	437	387	353	338	461	635	368	319

Oldham	30	22277	1825	1548	1047	720	695	663	853	753	751	468	461	460	436	379	361	367	421	444	403	393
Waltham Forest	30	20442	1809	1307	1056	717	736	664	692	534	537	432	429	440	436	402	356	339	355	285	444	327
St Helens	30	22137	1826	1532	1054	721	689	664	866	682	773	520	435	453	437	372	356	310	332	311	507	405
Sunderland	30	22048	1828	1398	1120	723	675	666	964	721	748	469	475	452	437	386	355	330	357	320	437	403
Barnsley	30	21661	1824	1563	1052	719	697	663	837	665	691	420	425	451	436	391	357	496	376	327	411	434
Southwark	29	20384	1807	1023	1099	717	608	662	762	459	630	458	455	427	436	411	347	381	311	385	327	286
Tameside	29	22354	1825	1759	1047	722	691	664	925	725	742	551	535	455	437	396	357	342	367	311	414	372
Doncaster	29	21870	1822	1404	1048	717	690	662	889	614	711	429	411	451	435	387	362	423	505	420	420	403
Lambeth	29	20870	1805	1064	1092	715	630	661	735	510	588	472	582	433	435	412	349	369	340	404	344	301
Torbay	29	20840	1824	1173	1046	724	695	665	627	628	437	464	323	441	438	358	359	336	546	344	336	513
Lewisham	29	20580	1814	1173	1065	722	681	666	777	557	620	400	455	440	438	398	355	406	319	398	452	272
Redcar and Cleveland	29	21338	1830	1407	1045	725	698	666	873	698	633	476	300	449	438	369	357	349	273	271	404	432
Bolton	28	21336	1825	1396	1048	720	689	663	725	721	666	488	478	453	436	375	356	361	354	359	456	425
Rotherham	28	21439	1826	1510	1047	721	690	664	806	652	616	460	338	453	437	381	359	356	440	400	465	423
Newcastle upon Tyne	28	21576	1818	1229	1076	712	648	661	1022	631	680	478	572	428	433	370	355	329	321	330	369	355
Coventry	28	21207	1818	1238	1068	714	662	661	757	634	642	471	489	444	434	397	352	343	386	386	345	340
Derby	28	21189	1827	1387	1073	718	652	664	722	621	598	488	343	447	435	377	356	329	408	467	411	329
Westminster	28	18399	1798	818	1103	705	582	657	540	380	397	468	564	409	430	407	345	349	232	237	216	359
Peterborough	28	20802	1815	1298	1059	715	654	662	645	604	572	452	394	443	435	376	356	325	336	308	387	469
Luton	28	20826	1813	1293	1064	714	665	662	661	579	549	472	467	443	434	383	354	320	395	435	391	331
Sheffield	28	20607	1822	1262	1062	717	667	663	735	618	549	420	466	446	435	369	358	540	405	395	310	305
Bristol, City of	27	20214	1815	1087	1076	714	638	663	691	554	519	423	557	443	435	358	358	487	348	305	338	304
Portsmouth	27	20996	1810	1291	1070	711	659	661	776	660	629	450	320	446	433	371	357	337	287	245	356	351
Enfield	27	19027	1818	1079	1064	727	668	669	561	511	426	395	334	432	439	403	357	334	333	329	324	257
Wakefield	27	21081	1826	1379	1054	721	676	665	825	649	648	409	437	450	437	391	355	448	324	336	425	384
Wirral	27	21590	1830	1246	1053	726	698	666	804	674	622	462	491	448	439	371	355	345	405	381	400	411
Southampton	27	20552	1810	1203	1064	710	665	661	751	585	619	463	422	442	433	370	356	334	316	295	334	356
Brent	27	19057	1807	1120	1062	713	665	662	521	490	406	405	440	424	433	412	354	340	269	353	302	276
Plymouth	27	20769	1818	1213	1061	717	674	663	732	558	517	483	424	440	435	363	354	349	458	326	350	368
Leeds	27	20727	1826	1358	1072	721	647	666	803	579	596	443	526	440	437	369	360	401	342	361	347	337
Gateshead	26	21564	1825	1362	1113	719	669	663	963	641	661	489	573	448	436	381	354	359	343	370	389	330
County Durham	26	21445	1826	1372	1106	721	691	664	851	699	676	464	476	448	436	383	357	337	349	335	362	442
Sefton	26	21842	1829	1317	1051	724	692	666	856	630	650	508	586	453	438	374	355	341	391	385	391	400
Greenwich	26	20245	1808	1152	1052	716	685	662	765	585	645	390	397	446	435	405	351	399	372	369	355	295
Camden	25	18988	1807	860	1103	715	590	661	585	405	454	450	801	415	435	408	348	348	212	259	260	289
Wigan	25	21989	1823	1555	1052	719	697	664	854	726	744	477	500	455	436	373	361	348	364	324	482	460
Telford and Wrekin	25	21557	1819	1364	1054	717	666	662	756	711	588	465	428	453	436	363	356	427	445	487	407	312
Stockton-on-Tees	25	20911	1828	1270	1057	722	673	665	827	661	556	460	400	445	437	371	359	323	270	379	397	368
Calderdale	25	21358	1826	1404	1062	721	668	664	766	652	679	436	380	451	437	373	360	389	387	363	413	471
Southend-on-Sea	25	20591	1818	1179	1061	720	685	665	677	584	525	400	371	445	437	367	353	480	379	298	386	450
Hammersmith and Fulham	24	19706	1814	1005	1096	720	606	666	694	431	552	447	388	422	438	414	350	395	255	294	271	320
Kirklees	24	20698	1824	1330	1051	718	687	662	686	673	554	438	369	448	436	371	356	375	431	442	398	380
Cornwall	24	19844	1824	1079	1049	724	684	665	519	570	424	457	441	435	438	356	355	355	413	405	281	432
Croydon	24	19732	1816	1112	1061	726	675	668	571	553	530	409	395	442	439	411	356	416	374	332	357	295
Darlington	24	21031	1828	1320	1063	725	662	666	741	657	533	463	328	448	438	379	360	353	265	325	348	546
Ealing	24	19209	1808	1130	1071	716	658	663	541	471	433	396	450	430	435	408	359	371	265	286	380	278

Brighton and Hove	23	20449	1811	967	1124	714	653	661	662	529	477	455	575	437	434	368	350	379	372	327	305	426
Kensington and Chelsea	23	17558	1812	665	1094	718	608	665	478	342	372	436	440	404	436	399	353	345	191	238	198	372
Isle of Wight	23	19217	1820	1054	1046	721	672	665	526	508	371	415	322	429	436	374	355	351	299	199	284	472
Dudley	23	20810	1824	1192	1058	721	693	664	627	636	585	470	463	444	437	370	352	346	484	434	343	359
Slough	23	20526	1815	1359	1079	716	622	663	682	554	570	444	420	446	435	391	356	322	387	350	452	319
Lancashire	22	20936	1823	1275	1057	719	674	663	681	630	573	496	675	443	436	371	356	331	380	409	396	373
Hounslow	22	19464	1805	1093	1077	713	633	662	604	483	497	413	402	432	434	413	353	412	323	334	342	271
Medway	22	20581	1817	1173	1048	719	688	664	730	548	646	435	345	448	436	362	355	346	323	301	356	336
Bournemouth	22	20267	1812	1118	1061	710	665	660	633	634	429	452	386	444	433	366	355	326	317	253	338	439
Bury	22	21767	1827	1483	1056	723	689	665	827	728	675	492	405	457	437	374	359	335	365	344	425	379
Thurrock	22	19882	1820	1220	1058	722	683	665	683	539	581	403	362	442	438	380	356	344	348	276	350	307
North Lincolnshire	21	20983	1823	1387	1058	719	677	662	726	655	610	429	319	446	436	386	353	337	339	353	367	451
Cumbria	21	20503	1823	1241	1053	719	668	663	620	628	501	478	529	442	436	375	356	368	377	301	282	403
North Tyneside	21	21319	1829	1262	1058	724	676	666	949	647	604	591	453	445	438	368	358	302	294	257	379	379
Norfolk	21	19556	1818	1036	1056	719	669	664	524	547	387	432	561	434	436	361	356	343	360	341	273	356
Lincolnshire	21	20031	1829	1257	1054	722	683	665	577	576	495	420	413	440	437	364	355	333	340	326	308	366
Northumberland	21	21004	1827	1309	1051	722	692	664	802	689	571	517	424	451	437	371	356	327	396	293	377	421
Redbridge	20	19321	1811	1201	1059	719	718	664	553	506	413	415	363	434	436	405	353	332	323	259	375	294
Herefordshire, County of	20	20010	1820	1153	1053	717	672	664	491	619	403	423	393	434	435	377	360	349	414	331	258	498
Reading	19	20467	1811	1111	1174	715	623	661	617	588	546	485	337	442	434	377	353	327	266	365	436	317
Warrington	19	21045	1822	1387	1074	717	640	662	740	669	597	459	394	453	435	393	354	323	316	361	478	381
Bedford	19	20434	1819	1221	1061	721	667	664	574	616	511	439	363	451	437	363	354	331	427	336	341	377
Stockport	19	20842	1825	1299	1063	722	666	664	728	633	567	519	409	443	437	373	360	335	346	375	322	376
Northamptonshire	19	20120	1827	1080	1059	720	667	663	599	564	533	432	472	442	437	369	356	345	370	395	350	317
Nottinghamshire	19	19998	1828	1116	1052	720	686	665	624	573	525	442	376	445	436	367	353	331	369	380	321	322
East Sussex	19	19765	1820	938	1097	723	685	666	540	489	391	427	461	433	437	427	354	393	408	309	295	426
Kent	19	19296	1819	1001	1102	721	673	665	560	531	475	438	477	436	437	353	352	341	294	293	315	310
Derbyshire	19	19940	1828	1213	1055	722	682	665	592	585	497	487	419	444	437	364	353	332	352	323	341	306
Suffolk	18	19001	1817	1020	1052	718	673	663	500	527	368	411	398	437	436	357	354	367	334	294	286	334
Wandsworth	18	19384	1820	1051	1093	729	636	670	653	483	532	433	372	432	441	407	354	348	251	277	365	277
Hillingdon	18	19753	1808	1098	1081	716	625	664	634	495	472	406	410	429	435	407	357	394	321	294	354	310
Cheshire West and Chester	18	20200	1827	1153	1069	724	653	665	647	585	520	469	476	444	438	371	354	326	301	351	325	373
Milton Keynes	18	19999	1819	1078	1072	721	629	665	655	554	555	498	375	444	437	374	353	314	343	378	459	346
Havering	18	19588	1818	1156	1060	726	675	668	662	513	515	421	332	440	440	398	356	332	300	247	364	276
Swindon	18	19941	1819	1180	1073	718	651	664	667	557	530	410	329	448	436	363	354	412	267	300	396	317
Barnet	18	18481	1815	1002	1070	720	666	666	503	453	372	397	369	428	437	397	354	333	247	259	318	296
Somerset	18	19252	1823	953	1053	722	678	666	474	537	394	433	357	439	437	353	358	363	391	343	271	395
Worcestershire	18	19900	1822	1033	1056	720	673	664	526	615	448	482	435	441	436	368	353	334	409	384	347	376
Solihull	17	19443	1826	1022	1066	724	648	666	568	527	404	508	447	434	438	366	357	327	473	355	264	306
Essex	17	19200	1822	1011	1062	722	671	666	561	527	459	402	440	436	437	360	354	357	314	331	299	307
Devon	17	19162	1823	971	1055	721	673	665	477	526	348	457	417	435	437	356	357	334	372	337	281	394
Shropshire	17	19834	1819	1149	1048	714	673	662	545	625	452	421	420	444	435	361	355	405	340	327	304	407
Staffordshire	16	19932	1819	1102	1053	716	679	662	549	581	476	513	475	442	435	366	353	335	405	469	311	334
Bexley	16	19213	1817	1097	1059	727	680	669	666	490	488	404	326	432	439	398	356	378	343	263	261	291
East Riding of Yorkshire	16	19883	1826	1214	1050	720	681	664	614	631	492	427	281	441	436	370	357	336	414	266	323	399
North Somerset	16	19954	1823	1031	1056	723	674	666	536	613	404	401	399	449	437	361	356	486	459	304	312	345

Trafford	15	19892	1826	1222	1071	721	640	664	696	587	517	500	435	441	437	380	355	343	290	278	279	337
Poole	15	19106	1820	955	1060	718	657	664	543	531	402	457	314	443	436	353	356	309	360	250	306	385
Bromley	15	18755	1819	1012	1063	728	668	669	545	486	425	383	339	433	440	400	356	370	286	232	278	303
Gloucestershire	15	19035	1822	986	1061	721	659	665	494	541	415	448	448	438	437	356	353	328	350	285	317	354
Warwickshire	15	19706	1821	1035	1067	718	652	663	546	566	429	466	455	439	436	380	353	322	382	394	325	354
Merton	15	18800	1815	1042	1068	721	655	667	573	446	475	435	326	429	438	406	353	343	295	293	296	289
North Yorkshire	15	19434	1824	1178	1052	717	666	663	521	625	423	420	357	440	435	362	360	345	335	306	283	406
Sutton	15	19003	1817	1016	1061	726	669	668	603	481	511	402	370	438	439	401	353	350	352	253	326	325
Dorset	14	18773	1821	888	1048	719	677	664	462	490	329	429	346	430	436	355	355	351	328	321	245	393
Harrow	14	18285	1811	1017	1055	717	677	664	458	399	368	390	310	421	435	400	358	324	287	388	265	284
Cheshire East	14	19697	1826	1093	1067	722	644	665	536	600	459	452	418	443	437	371	354	341	382	316	317	371
West Sussex	14	19164	1822	940	1072	723	660	666	519	520	396	410	399	437	438	355	353	389	340	300	302	360
Wiltshire	13	18887	1819	937	1052	717	673	664	472	521	377	401	337	440	435	354	356	435	353	315	315	336
Cambridgeshire	13	18472	1814	911	1066	716	647	664	483	485	386	432	392	438	434	360	353	320	318	290	253	303
Leicestershire	12	19110	1828	1070	1061	720	666	665	508	508	415	444	353	437	436	362	360	316	317	376	313	317
York	12	19629	1825	1209	1071	719	649	665	598	591	510	448	272	441	437	360	358	396	381	265	291	386
Central Bedfordshire	12	18745	1819	993	1063	721	673	665	536	481	455	424	367	436	436	367	354	321	300	229	301	270
Hertfordshire	12	18557	1822	945	1071	723	645	667	532	497	433	422	411	435	438	359	354	319	258	264	327	255
Bath and North East Somerset	12	18502	1821	902	1062	719	663	666	467	493	349	414	341	437	436	352	355	458	369	233	266	299
Hampshire	12	18404	1820	884	1063	722	654	666	482	495	389	429	405	434	437	376	353	326	286	221	271	300
Oxfordshire	12	18481	1815	846	1155	716	642	664	479	483	396	415	382	434	435	356	354	346	299	283	288	289
South Gloucestershire	11	18324	1819	914	1067	718	638	665	486	456	376	398	302	438	436	354	356	450	371	241	253	291
Kingston upon Thames	11	18140	1812	927	1075	721	650	666	511	427	385	377	300	430	438	405	354	360	343	255	314	310
Bracknell Forest	10	18522	1818	904	1072	722	636	666	572	504	421	451	273	443	437	358	356	305	329	242	342	200
West Berkshire	10	18710	1817	886	1072	720	623	666	522	518	426	431	253	437	436	368	355	336	329	235	309	315
Richmond upon Thames	10	17723	1817	767	1081	726	645	668	463	368	376	399	332	421	440	398	354	379	286	253	255	281
Buckinghamshire	10	18367	1821	867	1067	723	648	667	425	461	373	441	380	432	437	358	355	318	316	314	276	300
Rutland	10	19137	1814	1144	1034	703	679	657	431	527	436	489	373	436	429	348	357	312	417	377	266	274
Surrey	9	18114	1819	816	1114	721	639	666	457	470	375	406	345	434	437	352	354	338	302	251	315	271
Windsor and Maidenhead	9	18624	1819	1000	1080	720	627	665	532	527	400	433	285	440	436	361	357	316	237	281	354	339
Wokingham	6	17775	1819	817	1072	722	637	668	413	457	350	438	275	437	437	357	354	308	273	294	300	303

Statistically significantly lower than the England mean
Statistically significantly higher than the England mean

Appendix Figure 2: Age-standardised years of life lost (YLLs) rate per 100,000 population, for the 20 causes with the highest national YLL burden and decreasing Upper Tier Local Authority (UTLA) deprivation (Index of Multiple Deprivation [IMD] score), England both sexes, 2016

Upper Tier Local Authority	IMD score	All causes	Ischaemic heart disease	Trachea, bronchus, and lung cancer	Cerebrovascular disease	Chronic obstructive pulmonary disease	Alzheimer's disease and other dementias	Lower respiratory infections	Self-harm	Cirrhosis and other chronic liver diseases	Colon and rectum cancer	Breast cancer	Neonatal preterm birth	Congenital birth defects	Other cardiovascular and circulatory diseases	Road injuries	Pancreatic cancer	Drug use disorders	Other neoplasms	Oesophageal cancer	Brain and nervous system cancer	Prostate cancer
England (95% uncertainty interval)		8941 (8847 - 9029)	1040 (1018 - 1071)	623 (608 - 642)	431 (407 - 452)	408 (391 - 445)	345 (296 - 411)	336 (314 - 356)	326 (278 - 359)	302 (292 - 312)	286 (276 - 298)	271 (261 - 282)	262 (237 - 273)	238 (224 - 276)	174 (168 - 180)	170 (163 - 180)	170 (165 - 175)	167 (152 - 177)	149 (143 - 154)	143 (136 - 149)	139 (112 - 148)	139 (129 - 161)
Blackpool	42	14274	1708	1012	761	718	359	570	665	742	429	374	359	264	286	293	234	301	211	225	170	173
Knowsley	41	11033	1379	1112	509	746	354	481	256	362	356	293	218	269	171	192	191	178	171	195	150	139
Kingston upon Hull, City of	41	11501	1448	1086	602	732	353	450	443	336	357	305	257	224	208	199	198	172	190	179	170	148
Liverpool	41	11607	1268	1094	525	691	356	511	304	537	361	285	326	274	199	176	189	429	165	198	130	151
Manchester	41	11729	1483	1045	598	738	353	468	316	531	343	275	345	241	240	147	192	352	150	186	124	138
Middlesbrough	40	11693	1398	1073	629	670	355	494	415	413	396	310	271	223	193	209	201	164	224	171	144	152
Birmingham	38	10369	1254	690	490	482	347	391	280	379	304	263	571	329	176	159	176	277	150	160	122	134
Nottingham	37	11313	1337	876	533	635	353	420	364	421	352	305	417	359	229	171	206	146	183	176	151	154
Tower Hamlets	36	9629	1186	849	392	560	334	332	226	338	268	219	286	218	186	124	184	354	138	136	126	129
Hackney	35	9388	1128	705	368	435	333	327	237	355	244	242	354	169	187	136	176	323	142	126	136	145
Barking and Dagenham	35	10617	1364	944	447	634	348	471	290	319	308	325	245	261	209	175	204	71	204	152	151	153
Sandwell	35	10870	1346	791	559	563	355	407	331	405	327	306	418	351	192	193	193	124	165	168	132	142
Stoke-on-Trent	34	11847	1335	918	484	660	358	413	357	388	374	317	526	459	222	190	211	254	207	202	153	145
Blackburn with Darwen	34	11464	1619	806	595	671	357	436	436	378	351	271	357	231	181	211	186	269	179	196	171	134
Rochdale	34	11150	1497	852	619	634	360	440	389	406	313	303	276	235	205	203	188	226	183	175	143	144
Wolverhampton	33	11114	1356	767	574	513	354	418	369	473	356	314	415	294	214	196	204	154	188	182	152	162
Hartlepool	33	10961	1291	1011	588	560	353	426	383	480	337	287	277	184	158	200	213	163	195	155	131	128
Bradford	33	10379	1339	729	550	583	354	415	336	328	284	255	415	333	181	192	167	241	168	140	132	132
Leicester	33	10691	1494	687	537	520	355	470	334	405	296	287	396	325	197	180	180	157	157	152	122	145
Salford	33	11502	1424	1003	561	688	355	482	372	433	366	294	279	266	243	202	195	214	173	195	149	140
Newham	33	9408	1259	703	420	522	340	396	261	348	261	250	254	194	179	140	185	92	159	132	116	147
Islington	33	9381	1077	792	363	465	336	367	272	394	261	258	181	201	204	147	175	334	126	138	133	130
Halton	32	10598	1314	918	511	577	356	475	366	360	334	321	231	247	187	185	202	99	182	183	149	133
Haringey	31	8890	1072	591	396	381	337	357	318	336	248	272	233	166	188	201	174	155	116	114	134	142
North East Lincolnshire	31	10525	1384	773	516	551	352	382	448	420	330	301	249	272	161	224	180	122	191	173	169	165
South Tyneside	31	10223	1160	996	527	580	354	373	356	418	328	282	139	184	173	190	184	241	203	155	146	129

Walsall	30	10742	1362	717	487	532	351	366	311	333	324	301	537	345	181	200	175	207	165	176	146	141
Oldham	30	11130	1445	842	604	637	365	400	384	422	332	315	352	303	211	194	194	161	164	167	148	144
Waltham Forest	30	9327	1212	680	406	441	345	441	319	326	274	281	197	226	192	179	198	135	152	137	126	153
St Helens	30	11057	1428	854	541	658	358	504	396	486	361	318	219	230	187	207	196	130	159	192	145	148
Sunderland	30	10872	1293	949	574	631	357	434	394	418	340	288	239	241	184	197	191	207	188	146	139	135
Barnsley	30	10565	1458	826	526	582	356	408	425	250	325	290	240	262	201	203	199	179	165	147	151	136
Southwark	29	9036	932	746	322	514	333	324	278	327	253	245	292	199	199	154	181	181	130	131	108	123
Tameside	29	11156	1654	912	579	628	360	410	364	393	349	322	218	247	219	196	184	193	170	186	139	146
Doncaster	29	10832	1301	876	476	601	356	417	394	364	327	308	317	387	205	205	188	167	190	157	162	149
Lambeth	29	9461	973	721	375	480	339	341	292	367	255	248	307	222	221	166	183	278	139	126	112	154
Torbay	29	9831	1080	619	492	346	346	333	503	363	322	313	250	407	189	182	183	114	173	173	170	164
Lewisham	29	9494	1080	763	427	519	346	448	264	314	291	296	292	200	185	138	190	180	141	143	127	151
Redcar and Cleveland	29	10298	1300	861	555	527	354	401	423	361	360	329	188	158	184	209	183	72	196	167	149	148
Bolton	28	10225	1291	715	572	556	358	452	416	375	300	280	270	233	187	199	173	172	166	157	145	131
Rotherham	28	10432	1402	795	511	511	358	462	413	314	326	300	306	324	162	215	194	107	169	151	164	142
Newcastle upon Tyne	28	10443	1124	1003	486	563	337	366	346	415	319	288	243	220	219	160	187	282	174	141	144	129
Coventry	28	10110	1141	744	490	533	350	342	331	391	312	281	295	278	187	155	183	161	159	166	138	148
Derby	28	10164	1282	711	475	492	353	408	320	341	319	293	385	297	212	164	190	94	162	165	153	146
Westminster	28	7254	721	528	230	280	316	213	347	346	209	190	165	147	165	135	143	253	104	95	113	112
Peterborough	28	9764	1201	636	461	468	349	384	459	349	294	282	222	224	188	219	172	135	167	147	169	149
Luton	28	9751	1197	651	440	448	349	388	321	323	283	272	341	284	195	181	163	192	151	127	129	153
Sheffield	28	9482	1158	722	475	445	351	307	296	281	306	279	311	290	174	142	181	205	157	138	146	141
Bristol, City of	27	9085	993	679	414	418	348	335	295	323	292	268	222	222	184	133	178	254	142	152	148	140
Portsmouth	27	9999	1200	763	517	525	352	353	342	443	313	285	162	187	234	152	191	86	178	172	147	158
Enfield	27	8027	984	551	377	332	337	321	249	237	245	278	237	213	153	162	163	78	122	102	126	115
Wakefield	27	10002	1275	813	506	537	354	422	375	324	311	257	250	206	184	225	176	182	165	137	146	142
Wirral	27	10447	1143	793	533	516	352	397	403	482	320	316	282	300	179	194	199	185	179	178	152	142
Southampton	27	9567	1111	738	447	515	348	331	347	342	302	275	209	210	219	147	193	167	179	167	143	133
Brent	27	8104	1026	511	359	313	329	299	268	314	220	228	262	178	172	147	157	163	125	107	120	129
Plymouth	27	9727	1121	720	424	420	346	347	359	321	294	243	236	329	231	146	188	175	200	170	154	155
Leeds	27	9642	1253	788	437	486	345	344	328	347	292	260	280	232	183	176	173	247	163	135	144	146
Gateshead	26	10408	1256	947	497	548	353	386	322	372	321	274	279	240	200	189	168	276	189	146	142	129
County Durham	26	10322	1266	838	555	566	353	359	434	379	321	275	248	243	196	224	176	205	168	139	140	142
Sefton	26	10674	1214	844	491	542	358	388	392	428	349	311	292	287	178	201	188	254	164	182	155	154
Greenwich	26	9211	1058	751	450	540	351	352	286	328	273	263	279	252	170	151	182	127	147	136	134	140
Camden	25	7821	764	572	261	339	321	257	281	349	231	224	185	130	160	149	153	422	116	100	124	117
Wigan	25	10890	1452	842	582	631	359	479	450	410	352	292	235	248	217	185	167	187	171	190	134	142
Telford and Wrekin	25	10452	1263	744	561	484	359	404	303	364	329	304	400	334	193	170	176	120	200	176	152	156
Stockton-on-Tees	25	9887	1164	814	516	449	350	394	360	378	315	274	292	157	175	207	184	153	202	160	152	135
Calderdale	25	10330	1299	754	508	567	356	410	461	348	301	278	282	268	196	204	179	144	198	165	143	146
Southend-on-Sea	25	9527	1084	668	447	430	350	383	440	292	289	306	208	268	198	158	178	126	158	151	158	145
Hammersmith and Fulham	24	8604	913	680	296	441	328	269	311	413	262	237	220	171	196	175	171	118	117	135	121	122
Kirklees	24	9697	1225	676	529	452	353	395	372	305	281	252	354	314	185	194	169	135	174	132	138	132
Cornwall	24	8790	985	511	436	334	341	278	423	252	264	265	313	277	201	192	171	184	154	145	140	145
Croydon	24	8622	1020	561	419	431	347	354	287	284	258	274	238	251	191	152	168	135	122	114	136	131
Darlington	24	9954	1212	729	510	426	353	345	535	323	329	284	243	165	200	179	177	95	192	165	157	140

Ealing	24	8179	1035	531	341	337	336	377	271	342	233	230	202	170	163	150	155	170	117	106	119	127
Brighton and Hove	23	9380	877	649	395	380	343	302	415	369	309	274	244	262	194	160	191	288	158	154	148	148
Kensington and Chelsea	23	6578	572	467	211	272	310	195	362	327	202	197	162	108	126	122	138	148	114	80	121	110
Isle of Wight	23	8335	962	519	377	281	334	281	463	339	265	275	112	194	184	160	158	93	149	144	146	137
Dudley	23	9759	1094	619	494	485	350	340	350	340	329	305	339	367	169	182	173	149	157	161	135	148
Slough	23	9535	1263	671	410	462	351	449	310	364	264	248	267	282	201	192	169	164	158	139	125	145
Lancashire	22	9773	1170	670	498	466	348	393	365	427	289	266	322	261	189	173	162	320	138	166	134	126
Hounslow	22	8417	998	593	347	394	338	339	263	337	236	239	253	208	167	160	163	131	124	120	131	128
Medway	22	9600	1085	720	418	543	354	353	328	323	338	305	214	214	195	157	171	106	175	156	140	161
Bournemouth	22	9263	1024	622	493	336	350	335	428	435	323	277	168	191	188	140	185	155	160	159	154	157
Bury	22	10616	1377	815	582	566	362	422	370	368	336	305	259	251	200	197	188	113	169	181	147	153
Thurrock	22	8913	1123	674	405	481	348	347	298	247	270	277	178	239	164	144	155	124	158	136	135	141
North Lincolnshire	21	9982	1281	715	511	503	351	364	442	314	324	303	265	228	154	238	170	97	178	165	161	148
Cumbria	21	9360	1134	611	483	398	347	280	395	331	299	276	215	272	190	225	172	211	166	146	146	136
North Tyneside	21	10173	1157	934	505	496	351	376	370	413	320	271	175	188	207	165	182	189	200	144	140	153
Norfolk	21	8499	938	516	410	298	340	270	348	237	278	286	252	247	170	213	164	259	147	139	143	140
Lincolnshire	21	9014	1152	569	436	398	346	305	357	258	298	283	238	224	179	249	177	158	153	157	140	148
Northumberland	21	9908	1203	790	546	469	356	374	412	325	306	264	205	288	188	205	175	161	163	151	156	149
Redbridge	20	8259	1103	543	377	324	339	372	286	259	240	271	168	185	160	168	172	100	147	111	136	130
Herefordshire, County of	20	8976	1052	483	475	311	340	255	488	321	307	271	235	298	185	256	165	91	153	156	147	155
Reading	19	9348	1019	606	443	442	348	432	308	363	305	270	286	163	198	162	178	101	168	153	162	153
Warrington	19	9892	1280	727	517	485	358	475	372	336	295	290	280	203	187	192	167	69	156	163	140	152
Bedford	19	9443	1122	566	473	414	357	339	368	290	301	283	251	322	167	219	154	127	169	144	163	172
Stockport	19	9688	1193	717	487	461	349	319	366	349	322	300	289	232	199	187	189	117	161	179	151	146
Northamptonshire	19	9068	980	589	422	432	348	347	308	290	304	277	307	255	190	186	171	195	153	149	134	148
Nottinghamshire	19	9001	1013	615	435	427	350	318	314	288	296	297	285	253	168	203	173	127	157	141	140	149
East Sussex	19	8656	849	533	360	304	339	292	417	272	296	306	218	294	163	186	174	192	142	141	150	142
Kent	19	8268	908	551	398	380	341	313	302	264	272	272	207	181	185	157	176	204	132	146	132	139
Derbyshire	19	8921	1109	583	445	401	349	338	298	288	298	278	233	235	184	166	159	158	148	153	142	138
Suffolk	18	8018	921	493	391	280	343	283	325	228	282	275	206	223	167	180	164	144	145	135	146	135
Wandsworth	18	8336	958	640	350	428	337	362	268	282	246	241	197	139	176	125	165	119	123	105	136	137
Hillingdon	18	8667	1006	622	356	370	335	351	301	306	278	270	213	208	169	145	179	138	140	130	153	145
Cheshire West and Chester	18	9095	1047	636	439	414	349	322	364	315	314	281	263	198	186	185	164	168	155	162	140	141
Milton Keynes	18	8985	986	644	412	449	350	455	336	233	273	268	295	236	186	156	169	125	152	131	135	144
Havering	18	8537	1058	651	383	418	345	361	268	268	278	297	159	185	156	159	169	82	156	131	161	140
Swindon	18	8926	1085	656	417	430	354	393	308	266	299	267	218	149	191	148	162	112	162	165	139	147
Barnet	18	7538	907	494	324	283	334	315	288	223	236	264	174	158	141	146	164	106	122	102	137	114
Somerset	18	8246	860	467	402	306	344	269	385	250	286	264	252	280	165	207	160	121	144	141	151	136
Worcestershire	18	8814	933	518	471	353	347	344	367	301	304	277	290	293	167	198	161	124	149	148	144	142
Solihull	17	8370	919	559	385	310	340	261	298	256	285	275	267	360	135	186	156	107	154	140	150	130
Essex	17	8194	913	552	392	365	342	296	298	233	274	289	243	200	156	151	173	173	139	131	129	138
Devon	17	8146	876	470	392	262	340	278	385	239	283	273	249	242	162	158	168	173	147	136	147	151
Shropshire	17	8818	1047	537	480	356	349	301	399	252	311	283	237	227	156	234	169	115	164	163	153	149
Staffordshire	16	8890	1002	540	440	380	348	309	326	282	289	261	377	289	155	182	160	156	150	158	134	132
Bexley	16	8160	1002	655	361	393	338	259	283	254	265	256	169	227	158	130	165	76	143	140	138	137
East Riding of Yorkshire	16	8912	1108	605	488	394	346	320	390	264	289	296	177	303	163	212	162	69	151	141	163	137

North Somerset	16	8858	938	528	474	314	354	310	337	277	282	301	216	330	159	160	177	160	149	146	160	154
Trafford	15	8777	1116	683	439	410	346	277	328	312	295	248	194	180	180	147	152	139	152	149	139	132
Poole	15	8136	860	534	394	310	348	303	374	309	271	268	165	235	147	133	173	96	148	146	155	149
Bromley	15	7719	917	535	354	333	338	275	295	264	267	269	145	171	137	141	166	86	137	118	150	130
Gloucestershire	15	8055	891	486	403	323	344	314	345	263	270	261	200	221	142	184	170	192	132	124	142	129
Warwickshire	15	8627	934	537	422	332	345	322	346	283	261	270	304	268	160	192	161	135	149	138	135	136
Merton	15	7835	947	562	317	378	335	293	281	230	263	256	208	182	164	127	163	77	119	120	140	134
North Yorkshire	15	8429	1070	512	479	327	344	280	397	254	278	267	220	224	157	226	153	123	155	136	143	135
Sutton	15	7996	920	592	350	413	343	323	316	221	248	266	168	234	159	149	157	106	138	117	155	128
Dorset	14	7798	793	455	358	244	336	243	385	266	259	271	230	223	149	216	161	120	148	134	152	130
Harrow	14	7365	921	450	273	280	326	263	276	235	214	232	298	169	137	143	148	60	120	102	128	116
Cheshire East	14	8612	986	526	451	356	348	314	362	273	276	269	233	278	174	190	164	124	149	150	142	128
West Sussex	14	8176	848	511	386	305	343	299	351	255	274	294	212	227	171	180	179	146	140	140	149	139
Wiltshire	13	7915	842	465	387	289	346	312	327	231	270	265	225	248	150	211	158	107	139	127	143	132
Cambridgeshire	13	7513	814	474	349	294	343	251	294	207	250	232	204	210	166	170	159	134	132	116	133	132
Leicestershire	12	8148	964	499	370	321	343	310	308	252	264	257	291	203	152	179	159	110	148	131	145	136
York	12	8641	1102	588	446	408	346	289	377	247	315	263	181	274	180	167	150	62	161	142	144	140
Central Bedfordshire	12	7798	895	527	348	361	342	299	262	221	260	248	143	202	155	184	151	118	131	133	137	138
Hertfordshire	12	7601	848	523	360	338	340	324	247	208	257	263	180	168	165	134	161	148	122	114	130	135
Bath and North East Somerset	12	7512	807	459	358	262	343	263	290	223	258	234	147	249	154	138	158	120	138	127	156	145
Hampshire	12	7438	790	474	360	298	340	269	291	214	254	258	135	184	152	148	160	147	134	120	136	132
Oxfordshire	12	7494	752	470	347	302	340	285	280	241	265	248	201	205	150	153	161	128	131	121	136	137
South Gloucestershire	11	7389	818	478	321	285	343	251	282	224	244	229	158	245	131	159	144	87	137	136	153	131
Kingston upon Thames	11	7196	830	501	297	293	335	311	300	265	210	227	172	233	138	120	137	53	111	107	129	122
Bracknell Forest	10	7596	811	562	365	327	349	339	192	244	239	246	157	225	147	167	171	43	129	123	129	123
West Berkshire	10	7780	793	513	375	329	343	306	306	288	283	255	158	236	150	165	172	23	144	131	150	134
Richmond upon Thames	10	6734	671	453	242	284	327	253	272	283	207	216	173	180	127	118	129	83	107	101	117	110
Buckinghamshire	10	7384	773	418	327	282	337	274	292	213	240	254	229	220	155	158	160	133	134	108	139	126
Rutland	10	8131	1037	425	389	341	342	263	266	209	215	313	274	303	141	175	98	114	164	85	103	110
Surrey	9	7154	722	449	335	284	340	313	262	223	247	243	169	192	149	131	155	104	120	112	134	126
Windsor and Maidenhead	9	7748	904	522	385	306	345	351	330	271	266	245	204	135	144	172	156	54	121	120	137	135
Wokingham	6	6888	723	406	321	261	342	297	293	213	239	200	213	170	120	142	136	47	129	114	135	127

Statistically significantly lower than the England mean

Statistically significantly higher than the England mean

Appendix Figure 3: Age-standardised years lived with disability (YLDs) rate per 100,000 population for the 20 causes with the highest national YLD burden and decreasing Upper Tier Local Authority (UTLA) deprivation (Index of Multiple Deprivation [IMD]), England, both sexes, 2016

Upper Tier Local Authority	IMD score	All causes	Low back and neck pain	Skin and subcutaneous diseases	Migraine	Sense organ diseases	Depressive disorders	Anxiety disorders	Falls	Oral disorders	Asthma	Other musculoskeletal disorders	Drug use disorders	Diabetes mellitus	Bipolar disorder	Osteoarthritis	Schizophrenia	Other mental disorders	Cerebrovascular disease	Autism spectrum disorders	Upper respiratory infections	Other cardiovascular and circulatory diseases
England (95% uncertainty interval)	NA	11054 (8211 - 14261)	1820 (1277 - 2387)	1043 (705 - 1482)	719 (463 - 1007)	667 (462 - 922)	664 (454 - 910)	435 (304 - 591)	364 (247 - 509)	355 (217 - 551)	348 (228 - 499)	323 (216 - 461)	276 (200 - 356)	215 (146 - 300)	211 (131 - 313)	176 (116 - 254)	161 (120 - 201)	141 (96 - 202)	139 (99 - 176)	137 (93 - 192)	131 (78 - 203)	127 (87 - 178)
Blackpool	42	11300	1820	1019	718	695	661	436	459	356	359	291	343	243	206	177	155	141	152	132	131	125
Knowsley	41	11165	1835	1037	732	666	668	442	394	354	348	298	312	221	210	179	159	140	144	132	131	99
Kingston upon Hull, City of	41	11075	1819	1029	714	674	661	434	372	356	353	308	254	222	208	176	160	142	149	136	131	119
Liverpool	41	11133	1822	1041	716	660	657	346	413	353	346	309	388	226	209	178	143	142	146	134	131	111
Manchester	41	11280	1816	1050	711	634	659	433	439	351	353	292	356	237	207	177	161	142	156	133	131	131
Middlesbrough	40	11142	1825	1029	720	679	663	436	381	359	349	342	254	234	208	184	158	141	149	133	131	110
Birmingham	38	11141	1823	1035	720	667	664	437	420	354	344	298	347	241	211	174	160	141	144	135	131	105
Nottingham	37	11182	1819	1041	711	766	661	433	381	356	347	310	259	240	208	176	166	142	149	135	131	132
Tower Hamlets	36	11156	1795	1071	704	592	657	430	366	347	391	337	325	266	209	173	171	143	154	137	131	121
Hackney	35	11331	1806	1082	718	584	663	436	372	346	391	362	328	288	208	174	174	141	161	134	131	124
Barking and Dagenham	35	11183	1816	1112	725	687	666	438	351	355	378	325	245	214	211	175	159	141	131	149	131	111
Sandwell	35	11146	1821	1023	719	685	664	436	385	356	350	311	303	252	210	174	159	141	146	135	131	116
Stoke-on-Trent	34	11176	1817	1026	715	677	661	435	471	354	346	327	351	212	207	174	159	142	142	135	131	113
Blackburn with Darwen	34	11139	1822	1023	718	685	662	435	375	358	351	345	329	230	207	178	158	141	149	134	131	99
Rochdale	34	11170	1825	1022	722	695	664	437	368	356	349	317	315	231	208	178	157	141	147	134	131	117
Wolverhampton	33	11178	1820	1027	717	678	663	436	365	352	345	372	311	252	209	174	159	141	147	135	131	127
Hartlepool	33	11058	1829	1026	725	691	665	438	358	352	346	307	260	228	209	184	157	141	147	133	131	96
Bradford	33	11098	1825	1024	719	685	663	437	361	359	343	355	278	221	209	177	159	141	147	135	131	117
Leicester	33	11051	1826	1033	718	670	663	436	388	365	346	291	263	238	210	177	160	142	146	135	131	115
Salford	33	11169	1817	1037	713	658	660	434	399	354	351	301	325	228	208	177	160	142	150	135	131	127
Newham	33	10990	1801	1032	704	673	659	431	349	352	382	309	263	235	208	174	161	143	132	138	131	107
Islington	33	11229	1806	1077	715	597	663	435	373	348	386	340	327	246	209	174	167	141	146	134	131	129
Halton	32	11168	1826	1039	723	656	665	438	378	357	351	311	289	238	209	178	160	141	146	134	131	110
Haringey	31	11069	1810	1043	715	669	663	435	343	355	380	327	294	233	209	175	157	142	131	136	131	114
North East Lincolnshire	31	11042	1826	1029	720	667	664	437	342	362	345	330	236	240	209	176	160	141	146	135	131	107
South Tyneside	31	11119	1830	1077	724	698	665	438	361	355	341	313	269	221	210	184	157	141	140	134	132	120

Walsall	30	11134	1823	1021	720	692	664	437	382	353	345	312	331	244	210	174	159	141	140	135	131	116
Oldham	30	11147	1825	1020	720	695	663	436	366	361	344	334	300	225	208	178	157	141	148	135	131	118
Waltham Forest	30	11114	1809	1026	717	736	664	436	348	356	376	315	294	208	212	174	159	141	128	138	131	116
St Helens	30	11080	1826	1026	721	689	664	437	389	356	343	291	305	214	209	178	159	141	141	135	131	108
Sunderland	30	11176	1828	1087	723	675	666	437	365	355	356	306	269	225	211	184	159	141	147	135	131	117
Barnsley	30	11097	1824	1023	719	697	663	436	340	357	347	427	246	226	208	176	158	141	139	136	131	117
Southwark	29	11347	1807	1071	717	608	662	436	368	347	386	346	275	230	219	174	219	141	137	121	131	123
Tameside	29	11198	1825	1023	722	691	664	437	411	357	352	311	343	217	209	178	158	141	146	134	131	122
Doncaster	29	11039	1822	1024	717	690	662	435	346	362	347	375	244	221	209	177	160	142	138	136	131	123
Lambeth	29	11409	1805	1062	715	630	661	435	377	349	384	334	304	233	217	174	180	141	135	134	131	131
Torbay	29	11009	1824	1020	724	695	665	438	369	359	333	313	209	219	210	174	159	141	136	136	132	122
Lewisham	29	11086	1814	1040	722	681	666	438	335	355	377	365	275	217	221	175	159	141	130	122	131	107
Redcar and Cleveland	29	11040	1830	1024	725	698	666	438	368	357	341	319	229	227	210	184	157	141	142	135	131	122
Bolton	28	11111	1825	1023	720	689	663	436	379	356	344	328	306	223	208	178	158	141	149	135	131	119
Rotherham	28	11006	1826	1025	721	690	664	437	361	359	344	326	231	219	210	176	159	141	141	136	131	101
Newcastle upon Tyne	28	11134	1818	1047	712	648	661	433	375	355	344	305	289	209	209	184	161	142	145	137	131	144
Coventry	28	11097	1818	1037	714	662	661	434	374	352	354	316	328	250	209	174	161	142	144	137	131	108
Derby	28	11025	1827	1040	718	652	664	435	385	356	345	304	248	226	210	177	162	141	146	136	131	142
Westminster	28	11145	1798	1081	705	582	657	430	386	345	389	323	311	224	208	173	176	143	150	139	131	140
Peterborough	28	11038	1815	1030	715	654	662	435	361	356	342	304	259	240	208	177	162	142	143	137	131	128
Luton	28	11075	1813	1030	714	665	662	434	372	354	345	299	276	239	209	177	161	142	139	137	131	143
Sheffield	28	11125	1822	1039	717	667	663	435	348	358	342	453	261	216	212	176	161	141	143	137	131	123
Bristol, City of	27	11129	1815	1050	714	638	663	435	351	358	336	420	303	217	210	173	157	142	140	137	132	125
Portsmouth	27	10997	1810	1039	711	659	661	433	360	357	339	312	234	227	208	175	162	142	143	138	131	137
Enfield	27	11001	1818	1039	727	668	669	439	337	357	380	313	256	211	214	175	162	141	134	138	132	94
Wakefield	27	11078	1826	1029	721	676	665	437	337	355	351	396	255	216	210	176	160	141	143	135	131	126
Wirral	27	11142	1830	1024	726	698	666	439	363	355	342	318	306	218	210	178	157	140	141	135	131	121
Southampton	27	10985	1810	1039	710	665	661	433	371	356	339	309	255	210	210	175	163	142	137	138	131	140
Brent	27	10952	1807	1038	713	665	662	433	343	354	383	318	277	221	211	174	162	142	132	139	131	105
Plymouth	27	11042	1818	1032	717	674	663	435	378	354	336	320	249	220	210	174	160	142	134	137	131	145
Leeds	27	11085	1826	1047	721	647	666	437	360	360	344	358	279	215	211	177	161	141	142	136	131	126
Gateshead	26	11156	1825	1086	719	669	663	436	378	354	347	325	297	214	209	184	160	141	144	135	131	131
County Durham	26	11123	1826	1082	721	691	664	436	362	357	346	313	271	219	209	184	158	141	143	136	131	120
Sefton	26	11168	1829	1027	724	692	666	438	388	355	343	314	332	210	209	179	158	141	139	135	131	105
Greenwich	26	11034	1808	1030	716	685	662	435	330	351	378	362	270	216	210	174	160	142	135	123	131	106
Camden	25	11167	1807	1079	715	590	661	435	372	348	388	323	380	227	209	174	149	142	144	135	131	135
Wigan	25	11099	1823	1024	719	697	664	436	370	361	342	319	314	220	210	178	158	141	144	136	131	117
Telford and Wrekin	25	11105	1819	1032	717	666	662	436	371	356	339	374	307	224	210	174	162	141	150	137	131	130
Stockton-on-Tees	25	11024	1828	1035	722	673	665	437	365	359	343	303	247	222	211	184	160	141	145	136	131	125
Calderdale	25	11027	1826	1035	721	668	664	437	351	360	344	350	237	226	208	177	160	141	144	135	131	125
Southend-on-Sea	25	11064	1818	1028	720	685	665	437	333	353	337	417	245	208	212	177	160	141	137	136	132	125
Hammersmith and Fulham	24	11102	1814	1073	720	606	666	438	362	350	387	356	270	228	209	175	165	141	135	134	131	130
Kirklees	24	11001	1824	1027	718	687	662	436	355	356	341	341	234	223	208	176	159	142	144	136	131	126
Cornwall	24	11054	1824	1028	724	684	665	438	369	355	333	325	257	208	210	174	159	141	135	137	132	150
Croydon	24	11110	1816	1037	726	675	668	439	343	356	380	371	260	211	222	175	154	140	135	137	132	120
Darlington	24	11077	1828	1037	725	662	666	438	364	360	347	325	233	224	210	184	160	141	147	134	132	129

Ealing	24	11030	1808	1046	716	658	663	435	337	359	381	341	281	215	211	174	162	142	130	138	131	111
Brighton and Hove	23	11069	1811	1099	714	653	661	434	362	350	338	343	287	217	209	174	160	142	134	137	131	120
Kensington and Chelsea	23	10980	1812	1072	718	608	665	436	365	353	382	320	292	207	213	175	166	142	131	138	131	128
Isle of Wight	23	10882	1820	1018	721	672	665	436	349	355	341	326	229	213	212	175	161	141	131	138	131	113
Dudley	23	11051	1824	1023	721	693	664	437	372	352	340	319	314	226	210	174	160	141	142	137	131	121
Slough	23	10991	1815	1047	716	622	663	435	364	356	347	303	257	231	209	175	166	142	143	137	131	125
Lancashire	22	11163	1823	1034	719	674	663	436	391	356	343	306	355	214	210	178	159	141	132	136	131	132
Hounslow	22	11047	1805	1051	713	633	662	434	348	353	386	368	271	221	210	174	164	142	136	138	131	117
Medway	22	10982	1817	1024	719	688	664	436	351	355	335	320	240	221	211	175	160	141	130	138	131	109
Bournemouth	22	11004	1812	1037	710	665	660	433	363	355	337	303	231	222	209	173	161	143	141	139	131	139
Bury	22	11151	1827	1027	723	689	665	437	379	359	343	308	292	220	210	178	159	141	145	136	131	133
Thurrock	22	10969	1820	1026	722	683	665	438	337	356	342	320	238	222	211	177	161	141	134	137	132	121
North Lincolnshire	21	11001	1823	1029	719	677	662	436	348	353	348	314	222	245	208	176	159	142	144	136	131	111
Cumbria	21	11143	1823	1033	719	668	663	436	377	356	345	333	318	212	208	177	160	141	146	137	131	136
North Tyneside	21	11146	1829	1034	724	676	666	438	435	358	342	282	264	204	211	184	159	140	143	135	131	145
Norfolk	21	11058	1818	1033	719	669	664	436	355	356	337	317	301	216	209	177	161	141	137	138	132	138
Lincolnshire	21	11017	1829	1029	722	683	665	437	344	355	338	311	256	224	209	177	160	141	140	136	131	129
Northumberland	21	11096	1827	1028	722	692	664	437	388	356	342	302	263	209	210	184	159	141	143	137	131	129
Redbridge	20	11062	1811	1031	719	718	664	436	343	353	380	312	263	207	210	174	161	141	129	139	131	119
Herefordshire, County of	20	11034	1820	1030	717	672	664	435	348	360	344	321	302	221	210	174	162	142	144	139	131	141
Reading	19	11120	1811	1140	715	623	661	434	381	353	344	301	236	226	210	174	165	142	145	152	131	145
Warrington	19	11153	1822	1044	717	640	662	435	366	354	354	301	325	226	210	178	163	141	151	137	131	128
Bedford	19	10992	1819	1034	721	667	664	437	356	354	337	307	236	232	211	177	161	141	143	137	131	128
Stockport	19	11154	1825	1038	722	666	664	437	401	360	345	307	291	204	211	178	161	141	146	137	131	147
Northamptonshire	19	11052	1827	1033	720	667	663	437	352	356	341	319	277	218	210	177	161	141	141	137	132	140
Nottinghamshire	19	10997	1828	1028	720	686	665	436	355	353	338	309	248	219	210	177	160	141	138	138	131	134
East Sussex	19	11109	1820	1075	723	685	666	437	353	354	403	355	268	190	210	175	160	141	128	124	131	97
Kent	19	11028	1819	1077	721	673	665	437	361	352	333	316	273	204	212	175	161	141	133	124	132	135
Derbyshire	19	11020	1828	1032	722	682	665	437	380	353	338	307	261	216	211	177	160	141	140	137	132	137
Suffolk	18	10983	1817	1029	718	673	663	436	345	354	335	336	254	215	211	177	162	142	136	139	132	144
Wandsworth	18	11048	1820	1069	729	636	670	441	355	354	382	320	254	218	214	175	163	140	133	134	132	110
Hillingdon	18	11086	1808	1054	716	625	664	435	344	357	383	356	272	223	212	174	165	142	139	139	131	99
Cheshire West and Chester	18	11104	1827	1043	724	653	665	438	373	354	345	304	308	213	211	178	162	141	146	137	131	144
Milton Keynes	18	11014	1819	1048	721	629	665	437	391	353	344	294	250	227	210	175	165	141	143	138	131	140
Havering	18	11050	1818	1034	726	675	668	440	347	356	378	311	249	202	214	175	162	140	130	137	132	121
Swindon	18	11016	1819	1042	718	651	664	436	345	354	337	367	217	226	211	173	163	141	140	138	131	140
Barnet	18	10943	1815	1045	720	666	666	437	340	354	377	313	263	203	214	175	163	141	129	140	132	98
Somerset	18	11006	1823	1032	722	678	666	437	355	358	333	331	236	214	210	174	161	141	134	139	132	137
Worcestershire	18	11086	1822	1032	720	673	664	436	381	353	341	308	311	211	210	174	162	141	144	139	132	134
Solihull	17	11073	1826	1042	724	648	666	438	400	357	341	302	340	211	213	174	164	141	142	138	132	124
Essex	17	11006	1822	1033	722	671	666	437	340	354	336	328	267	213	212	177	162	141	135	138	132	124
Devon	17	11016	1823	1035	721	673	665	437	371	357	334	310	244	204	211	174	161	141	133	138	132	140
Shropshire	17	11017	1819	1029	714	673	662	435	347	355	338	362	305	206	209	174	162	142	144	139	131	113
Staffordshire	16	11043	1819	1030	716	679	662	435	398	353	339	306	319	213	211	174	161	142	140	128	131	120
Bexley	16	11053	1817	1035	727	680	669	439	340	356	376	345	250	196	215	175	162	140	129	124	132	118
East Riding of Yorkshire	16	10971	1826	1028	720	681	664	436	349	357	341	312	213	214	209	177	161	141	143	139	131	128

North Somerset	16	11096	1823	1032	723	674	666	437	340	356	335	419	239	212	212	174	161	141	139	138	132	111
Trafford	15	11115	1826	1047	721	640	664	437	391	355	349	315	296	212	210	178	163	141	148	137	131	131
Poole	15	10971	1820	1037	718	657	664	436	368	356	334	292	218	213	212	174	163	142	136	139	132	140
Bromley	15	11036	1819	1042	728	668	669	440	333	356	378	341	253	196	216	175	162	140	132	125	132	111
Gloucestershire	15	10980	1822	1041	721	659	665	437	364	353	335	305	256	211	211	174	162	141	138	139	132	119
Warwickshire	15	11080	1821	1040	718	652	663	436	374	353	347	301	320	221	210	174	163	141	144	138	132	135
Merton	15	10965	1815	1047	721	655	667	438	360	353	380	318	249	203	214	175	163	141	129	138	132	125
North Yorkshire	15	11005	1824	1033	717	666	663	435	348	360	340	319	234	211	209	177	162	142	146	139	131	133
Sutton	15	11006	1817	1040	726	669	668	439	341	353	377	325	264	190	214	175	162	141	131	138	132	124
Dorset	14	10976	1821	1029	719	677	664	436	356	355	333	323	226	199	210	174	161	141	132	140	131	147
Harrow	14	10920	1811	1035	717	677	664	435	339	358	378	307	250	199	214	174	163	142	125	141	132	104
Cheshire East	14	11085	1826	1044	722	644	665	437	368	354	346	314	294	206	211	178	163	141	149	138	131	145
West Sussex	14	10989	1822	1049	723	660	666	438	346	353	334	351	253	200	212	175	162	141	134	138	132	118
Wiltshire	13	10973	1819	1031	717	673	664	435	341	356	333	381	230	208	210	174	162	142	134	140	131	130
Cambridgeshire	13	10959	1814	1043	716	647	664	434	356	353	338	300	258	213	211	177	164	142	137	140	132	145
Leicestershire	12	10962	1828	1037	720	666	665	436	363	360	338	299	243	208	212	177	163	141	139	139	132	136
York	12	10988	1825	1048	719	649	665	437	363	358	341	354	210	205	212	177	163	141	145	138	131	142
Central Bedfordshire	12	10948	1819	1034	721	673	665	436	347	354	339	303	248	215	212	177	162	141	133	139	131	140
Hertfordshire	12	10956	1822	1047	723	645	667	438	350	354	338	301	263	205	214	177	164	141	137	139	132	139
Bath and North East Somerset	12	10990	1821	1044	719	663	666	436	351	355	334	399	220	203	214	174	163	141	135	140	132	146
Hampshire	12	10966	1820	1041	722	654	666	437	359	353	355	305	258	203	213	175	164	141	135	139	132	135
Oxfordshire	12	10987	1815	1132	716	642	664	435	351	354	335	318	254	213	212	175	137	142	135	154	131	140
South Gloucestershire	11	10934	1819	1046	718	638	665	436	343	356	335	390	215	202	212	174	165	142	135	141	131	119
Kingston upon Thames	11	10944	1812	1053	721	650	666	438	333	354	380	333	247	193	215	174	164	141	130	139	132	117
Bracknell Forest	10	10926	1818	1049	722	636	666	437	370	356	337	288	230	206	214	175	165	141	138	139	132	130
West Berkshire	10	10929	1817	1049	720	623	666	436	359	355	342	311	230	217	213	175	167	142	143	140	132	140
Richmond upon Thames	10	10988	1817	1060	726	645	668	440	345	354	378	344	249	194	216	175	164	141	126	138	132	132
Buckinghamshire	10	10983	1821	1044	723	648	667	437	365	355	336	298	247	200	213	175	164	141	134	139	132	143
Rutland	10	11006	1814	1019	703	679	657	429	390	357	336	285	259	182	211	177	162	143	138	143	131	156
Surrey	9	10960	1819	1093	721	639	666	437	347	354	335	314	242	196	213	175	165	141	135	140	132	126
Windsor and Maidenhead	9	10876	1819	1054	720	627	665	436	360	357	338	297	232	207	214	175	166	142	142	140	132	132
Wokingham	6	10887	1819	1051	722	637	668	437	367	354	336	291	228	202	217	175	166	141	136	141	132	133

Note: no estimates are statistically significantly different from England mean

Appendix Figure 4: Attributable risk for age-standardised all-cause years of life lost (YLLs) rate per 100,000 population for nine major risk factors, and Upper Tier Local Authority (UTLA) level Index of Multiple Deprivation (IMD) score, for 150 UTLAs in England, 2016

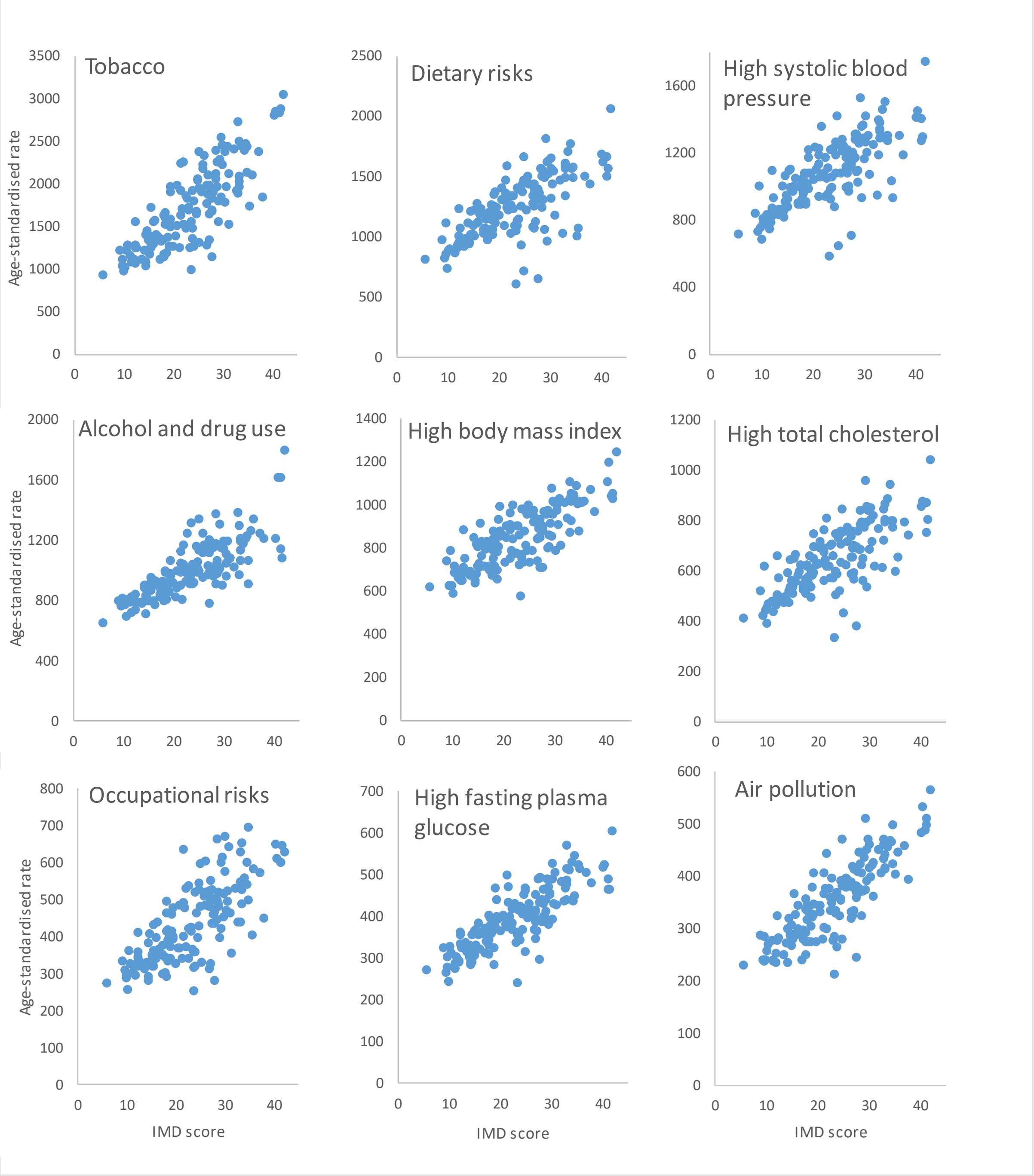

Appendix Figure 5: Age-standardised attributable risk for all-cause years of life lost (YLLs) rate per 100,000 population in order of decreasing Upper Tier Local Authority (UTLA) deprivation (Index of Multiple Deprivation [IMD]), England, both sexes, 2016

Upper Tier Local Authority	IMD score	All risk factors	Tobacco	Dietary risks	High systolic blood pressure	Alcohol and drug use	High body-mass index	High total cholesterol	Occupational risks	High fasting plasma glucose	Air pollution	Child and maternal malnutrition	Low physical activity	Impaired kidney function	Unsafe sex	Low bone mineral density	Other environmental risks	Sexual abuse and violence	Unsafe water, sanitation, and handwashing
**England (95% uncertainty interval)**	**NA**	**4694 (4516-4869)**	**1593 (1526-1669)**	**1192 (1060-1326)**	**1032 (926-1134)**	**967 (704-1281)**	**807 (522-1102)**	**614 (517-725)**	**406 (359-451)**	**374 (257-514)**	**328 (267-396)**	**296 (274-307)**	**166 (88-253)**	**142 (131-153)**	**53 (52-56)**	**37 (28-39)**	**35 (21-57)**	**6 (6-9)**	**5 (3-7)**
Blackpool	42	8017	3061	2065	1746	1804	1246	1044	627	605	566	392	270	221	96	71	62	13	8
Knowsley	41	6368	2891	1578	1297	1089	1030	806	646	466	499	242	212	180	72	51	63	6	7
Kingston upon Hull, City of	41	6558	2840	1675	1410	1143	1051	870	600	490	510	292	230	198	67	45	50	7	6
Liverpool	41	6879	2842	1506	1274	1618	1041	753	602	464	488	370	200	178	74	57	60	8	7
Manchester	41	7095	2867	1630	1456	1619	1196	878	610	523	533	376	232	189	78	65	58	8	6
Middlesbrough	40	6665	2811	1689	1414	1220	1104	857	650	517	483	303	226	206	91	45	45	9	6
Birmingham	38	5805	1863	1442	1194	1217	965	743	450	479	395	645	195	163	56	54	45	8	6
Nottingham	37	6301	2386	1513	1309	1253	1073	792	572	504	459	477	211	168	66	47	84	8	5
Tower Hamlets	36	5637	2121	1077	935	1348	1019	656	585	513	446	332	180	158	52	36	19	5	4
Hackney	35	5282	1748	1012	1034	1265	1012	600	405	524	404	399	171	167	75	32	16	8	4
Barking and Dagenham	35	5856	2455	1494	1276	917	877	775	695	448	497	276	207	168	70	39	24	6	7
Sandwell	35	5974	2147	1589	1311	1068	1015	801	498	546	424	475	212	187	64	48	51	7	7
Stoke-on-Trent	34	6556	2479	1570	1288	1232	1002	800	602	435	465	602	209	166	71	72	58	8	6
Blackburn with Darwen	34	6470	2411	1782	1509	1213	1089	942	542	528	467	397	250	215	73	44	51	6	7
Rochdale	34	6306	2447	1718	1463	1178	1048	889	558	512	457	305	234	217	76	44	52	8	6
Wolverhampton	33	6132	2104	1615	1344	1215	1050	805	526	570	415	480	215	202	59	40	49	9	7
Hartlepool	33	6272	2513	1571	1331	1198	1013	797	654	477	441	312	209	202	83	38	41	9	7
Bradford	33	5704	2045	1502	1295	1068	927	796	487	440	415	445	209	174	63	36	39	9	6
Leicester	33	5915	1977	1600	1383	1142	1028	867	439	513	460	455	229	185	76	50	70	7	7
Salford	33	6586	2740	1617	1400	1304	1109	846	629	485	471	310	223	173	67	52	57	6	6
Newham	33	5224	1908	1351	1191	979	873	723	549	468	433	286	190	182	58	33	20	7	6
Islington	33	5287	1927	1032	954	1391	940	615	439	436	405	206	166	144	84	40	15	10	4
Halton	32	5849	2420	1481	1254	1026	1012	768	532	482	450	267	205	180	67	44	51	6	7
Haringey	31	4719	1533	1183	1079	1086	810	616	356	425	361	263	167	164	61	32	15	9	5
North East Lincolnshire	31	5711	2128	1547	1310	1083	1027	820	462	505	426	280	215	213	72	32	38	8	4

South Tyneside	31	5843	2448	1447	1208	1200	906	718	641	429	420	155	189	174	59	40	41	8	6
Walsall	30	5875	1980	1576	1273	1051	972	804	496	526	400	611	209	191	55	43	47	7	7
Oldham	30	6219	2390	1662	1421	1117	999	853	524	490	460	394	229	196	72	43	51	8	6
Waltham Forest	30	4973	1796	1327	1165	965	789	687	469	393	417	221	184	154	60	37	20	7	6
St Helens	30	6141	2460	1639	1372	1147	1008	839	575	451	470	250	221	191	76	52	52	6	8
Sunderland	30	6133	2469	1532	1293	1203	1017	793	672	456	390	272	207	179	65	43	39	9	7
Barnsley	30	5723	2238	1630	1364	910	956	857	515	470	450	257	226	171	70	32	43	6	6
Southwark	29	5018	1848	967	935	1176	853	533	454	382	374	337	144	143	76	37	16	12	4
Tameside	29	6418	2550	1819	1531	1154	1078	958	614	488	511	238	252	189	67	56	55	8	7
Doncaster	29	5853	2305	1493	1261	1017	911	772	599	439	437	359	203	165	68	35	43	8	5
Lambeth	29	5218	1812	1071	1026	1315	857	561	421	410	372	353	153	152	70	41	16	10	4
Torbay	29	4870	1562	1312	1096	1003	803	649	398	400	325	286	176	156	77	39	58	7	4
Lewisham	29	5231	1980	1248	1093	1054	799	622	481	399	445	335	172	143	68	30	19	5	5
Redcar and Cleveland	29	5668	2270	1566	1313	988	974	795	521	463	406	206	209	191	83	42	38	8	5
Bolton	28	5569	2069	1511	1287	1068	953	783	487	431	423	297	205	161	70	42	44	8	6
Rotherham	28	5615	2134	1577	1319	916	958	840	498	438	446	348	216	173	65	38	40	7	5
Newcastle upon Tyne	28	5962	2410	1318	1172	1382	968	687	662	386	370	270	180	154	58	41	37	8	5
Coventry	28	5507	1964	1374	1168	1182	968	686	448	493	377	334	183	166	65	40	46	8	6
Derby	28	5437	1914	1404	1207	968	969	760	502	432	409	440	200	150	55	41	69	7	5
Westminster	28	3822	1151	662	711	1153	707	382	281	296	244	188	112	96	60	35	10	9	3
Peterborough	28	5063	1706	1314	1125	1022	902	695	467	494	382	246	189	171	52	40	25	8	6
Luton	28	5179	1688	1311	1139	1026	874	690	437	489	369	392	188	175	74	44	26	7	5
Sheffield	28	5120	1847	1328	1155	1023	891	693	461	388	360	343	184	136	52	30	35	8	4
Bristol, City of	27	4873	1664	1133	1004	1173	846	592	436	365	335	247	161	136	56	31	63	7	5
Portsmouth	27	5454	1995	1364	1188	1174	934	700	526	452	414	173	192	174	56	39	38	9	4
Enfield	27	4101	1350	1075	977	787	711	567	327	346	324	268	156	130	48	26	14	5	4
Wakefield	27	5483	2114	1443	1226	994	908	760	552	412	417	282	199	160	62	29	40	6	6
Wirral	27	5649	2122	1416	1187	1204	888	691	497	413	379	320	185	167	68	38	46	8	6
Southampton	27	5190	1891	1265	1083	1124	844	653	504	385	385	233	177	158	56	42	37	8	5
Brent	27	4292	1292	1095	1001	976	739	586	314	367	320	295	157	138	67	27	14	8	3
Plymouth	27	5085	1777	1285	1120	1036	838	655	482	401	332	263	176	157	58	43	66	7	4
Leeds	27	5403	1989	1351	1175	1133	931	739	522	396	390	310	192	150	55	34	37	7	5
Gateshead	26	5877	2338	1445	1247	1170	975	760	604	414	394	317	196	165	70	45	39	5	5
County Durham	26	5708	2189	1504	1285	1120	949	774	519	430	379	279	202	173	58	41	36	8	5
Sefton	26	5853	2240	1459	1214	1252	917	718	485	404	396	335	191	164	67	47	48	9	6
Greenwich	26	5050	1923	1234	1080	962	784	623	511	396	389	320	170	155	51	27	19	5	5
Camden	25	4232	1315	721	648	1344	732	431	330	315	279	208	120	112	68	34	11	6	3
Wigan	25	6123	2387	1667	1423	1143	996	847	596	467	470	262	228	186	57	45	50	6	7
Telford and Wrekin	25	5578	1962	1474	1252	1003	979	751	481	462	382	455	203	175	50	38	47	7	5
Stockton-on-Tees	25	5466	2054	1379	1188	1061	954	708	546	418	354	332	188	169	66	38	33	5	5
Calderdale	25	5553	2064	1444	1268	1014	954	760	528	451	392	315	204	182	66	35	38	6	5
Southend-on-Sea	25	4866	1691	1257	1084	993	789	635	427	404	356	236	176	147	62	29	25	10	5

Hammersmith and Fulham	24	4663	1659	936	878	1167	814	522	396	368	325	255	141	140	68	36	13	7	4
Kirklees	24	5199	1807	1400	1228	922	876	729	464	424	370	400	195	172	58	33	35	8	5
Cornwall	24	4390	1283	1149	1001	920	730	589	318	341	266	361	159	139	54	37	48	9	3
Croydon	24	4494	1492	1146	1064	911	770	596	359	360	334	268	163	144	68	30	15	6	4
Darlington	24	5292	1931	1408	1220	1042	983	746	520	414	362	270	192	152	56	37	31	8	4
Ealing	24	4310	1360	1097	989	1015	762	597	315	359	330	228	157	144	52	25	14	7	4
Brighton and Hove	23	4887	1566	1053	932	1322	752	507	359	356	285	283	147	127	92	41	32	10	4
Kensington and Chelsea	23	3309	1001	618	585	972	578	333	252	239	213	180	93	84	60	30	9	6	2
Isle of Wight	23	4094	1264	1104	958	918	723	569	367	336	278	124	152	138	42	27	27	6	4
Dudley	23	5064	1697	1349	1114	984	861	657	425	421	349	390	178	151	53	40	40	7	7
Slough	23	5026	1737	1236	1052	1022	900	704	414	431	396	300	191	147	37	38	34	4	5
Lancashire	22	5372	1836	1353	1173	1254	901	703	421	384	358	344	185	154	54	41	40	7	5
Hounslow	22	4440	1485	1038	947	982	775	572	340	368	335	292	155	140	59	29	14	7	4
Medway	22	5084	1870	1271	1080	931	777	618	537	416	377	240	175	171	56	36	37	6	4
Bournemouth	22	4822	1543	1230	1070	1176	843	617	373	397	299	191	170	151	77	39	58	8	4
Bury	22	5847	2267	1594	1361	1042	996	807	529	470	442	290	218	193	69	46	49	8	6
Thurrock	22	4700	1717	1252	1062	814	785	641	477	433	375	198	175	153	56	31	25	6	5
North Lincolnshire	21	5315	1928	1473	1234	910	965	761	493	497	406	292	202	205	68	33	36	6	5
Cumbria	21	4871	1636	1302	1137	1055	882	677	401	375	302	239	180	155	56	40	37	9	4
North Tyneside	21	5637	2250	1372	1191	1127	910	691	634	385	366	194	187	153	65	64	38	8	7
Norfolk	21	4298	1264	1100	946	994	751	561	341	380	280	281	153	139	51	34	20	7	4
Lincolnshire	21	4644	1527	1311	1105	888	847	688	369	403	332	261	180	158	54	35	56	6	4
Northumberland	21	5297	1991	1417	1236	1000	900	714	486	398	345	237	195	158	48	53	34	6	5
Redbridge	20	4227	1392	1191	1046	833	733	627	371	365	344	183	169	148	45	32	16	6	5
Herefordshire, County of	20	4396	1274	1249	1083	925	858	625	329	392	274	267	170	147	52	32	32	5	4
Reading	19	4847	1599	1121	991	1066	877	594	393	399	326	332	166	149	61	45	30	6	5
Warrington	19	5358	1980	1380	1224	968	994	746	479	440	406	322	201	160	60	38	42	8	6
Bedford	19	4716	1500	1264	1082	929	850	647	401	468	348	277	182	167	47	37	22	6	5
Stockport	19	5194	1914	1357	1178	988	934	694	462	375	380	329	188	138	55	47	41	5	4
Northamptonshire	19	4716	1547	1146	1019	977	805	583	404	369	322	348	160	143	50	35	57	7	6
Nottinghamshire	19	4631	1579	1196	1019	863	783	602	415	381	325	319	165	145	47	40	60	6	5
East Sussex	19	4178	1274	1027	898	980	653	496	340	283	274	249	139	114	58	31	27	7	4
Kent	19	4249	1385	1067	909	916	698	534	371	324	304	227	148	132	48	33	29	6	4
Derbyshire	19	4645	1539	1269	1084	900	819	661	396	376	325	262	177	135	48	45	59	6	5
Suffolk	18	3968	1199	1073	917	815	712	544	334	366	276	226	150	123	46	30	19	5	4
Wandsworth	18	4479	1597	1026	968	995	779	551	390	359	337	229	151	131	61	35	14	7	4
Hillingdon	18	4499	1530	1049	956	992	809	578	417	376	333	240	157	134	47	27	14	5	4
Cheshire West and Chester	18	4805	1673	1205	1051	999	875	620	415	359	318	297	169	133	58	38	36	7	5
Milton Keynes	18	4636	1619	1056	928	855	786	561	415	394	345	342	161	144	49	48	32	4	6
Havering	18	4434	1641	1176	998	801	748	621	494	342	357	180	164	133	46	31	16	5	4
Swindon	18	4684	1637	1187	1031	873	841	624	462	402	337	250	172	143	45	28	60	5	5

Barnet	18	3761	1183	983	895	809	672	522	291	309	275	194	142	120	40	26	12	4	3
Somerset	18	3973	1148	1034	894	839	690	511	304	332	249	289	144	125	47	34	43	6	4
Worcestershire	18	4412	1351	1156	999	930	795	568	345	348	292	334	155	133	44	42	33	6	5
Solihull	17	4166	1338	1077	903	832	797	546	352	340	279	309	147	127	42	43	34	5	5
Essex	17	4196	1361	1066	908	842	716	537	376	369	298	270	149	129	47	29	21	6	4
Devon	17	3986	1129	1038	895	890	699	523	299	312	240	287	144	122	51	36	44	7	4
Shropshire	17	4444	1401	1242	1049	902	830	623	351	357	290	272	170	134	47	30	34	8	4
Staffordshire	16	4614	1414	1196	1008	929	795	599	366	363	300	425	162	141	48	49	35	8	5
Bexley	16	4241	1579	1129	975	781	716	580	441	322	345	187	155	124	41	26	17	4	3
East Riding of Yorkshire	16	4510	1571	1284	1110	802	829	664	396	383	327	196	180	160	54	33	30	7	4
North Somerset	16	4311	1299	1124	995	942	766	557	340	356	286	241	157	142	46	27	50	7	5
Trafford	15	4756	1728	1228	1095	958	913	651	432	368	366	216	176	135	48	45	38	6	3
Poole	15	3995	1268	1014	878	868	729	512	318	330	287	186	141	122	66	38	50	6	4
Bromley	15	3905	1309	1029	925	800	710	533	364	305	281	164	146	122	48	23	14	5	3
Gloucestershire	15	4009	1183	1025	887	906	712	529	318	325	267	223	148	122	47	38	46	6	4
Warwickshire	15	4372	1346	1098	953	917	807	560	347	364	289	352	152	138	46	39	33	6	6
Merton	15	4044	1404	1025	918	806	716	543	352	316	307	232	147	120	40	33	14	4	4
North Yorkshire	15	4270	1345	1220	1067	844	819	645	325	356	288	246	172	144	46	30	26	6	3
Sutton	15	4045	1462	1024	909	805	678	533	407	290	320	184	145	115	36	27	15	4	4
Dorset	14	3706	1050	951	816	837	648	474	291	283	235	267	130	115	45	30	43	6	3
Harrow	14	3675	1095	976	856	718	634	522	281	290	272	339	140	107	38	23	13	5	4
Cheshire East	14	4366	1418	1118	1005	910	850	592	384	331	299	267	160	132	48	35	30	7	4
West Sussex	14	3992	1227	994	864	893	681	498	330	303	272	239	140	118	45	29	26	7	4
Wiltshire	13	3811	1117	990	868	790	675	496	314	313	250	257	140	120	46	28	43	6	5
Cambridgeshire	13	3719	1122	923	833	797	690	475	317	337	249	228	134	115	38	36	18	5	4
Leicestershire	12	4107	1259	1080	935	798	751	569	334	325	283	335	152	123	47	36	49	5	5
York	12	4470	1561	1239	1099	850	883	661	413	354	325	202	176	149	44	34	29	5	3
Central Bedfordshire	12	3874	1290	1013	877	747	686	507	357	362	279	160	145	136	41	35	20	4	5
Hertfordshire	12	3860	1260	956	856	795	706	495	346	334	278	202	138	115	39	33	19	5	4
Bath and North East Somerset	12	3592	1085	942	831	794	686	482	321	289	235	164	133	107	43	26	43	4	4
Hampshire	12	3659	1119	919	792	806	647	464	328	292	251	186	131	114	41	32	24	6	3
Oxfordshire	12	3641	1107	872	747	828	648	437	296	303	241	226	126	108	38	30	23	5	4
South Gloucestershire	11	3531	1097	900	795	722	685	474	319	287	238	176	131	110	43	24	45	4	4
Kingston upon Thames	11	3608	1174	891	832	795	659	478	304	274	277	195	130	116	34	20	12	5	4
Bracknell Forest	10	3739	1297	911	809	697	683	466	329	312	270	175	133	123	56	38	28	6	4
West Berkshire	10	3764	1224	891	778	776	715	455	361	326	256	182	134	113	41	33	25	4	4
Richmond upon Thames	10	3312	1030	747	690	809	586	390	257	243	240	199	107	96	43	23	10	5	3
Buckinghamshire	10	3535	993	866	761	780	627	443	290	276	236	261	126	109	41	35	22	6	4
Rutland	10	4041	1123	1119	1004	817	787	618	295	301	285	333	160	190	17	39	42	7	5
Surrey	9	3460	1046	827	736	769	626	419	310	264	241	191	121	100	40	28	22	5	4

Windsor and Maidenhead	9	3894	1228	984	845	800	737	517	336	324	288	236	146	121	46	32	26	6	4
Wokingham	6	3256	939	816	717	654	617	413	274	272	230	247	119	102	28	32	21	3	4

Statistically significantly lower than the England mean
Statistically significantly higher than the England mean

Appendix Figure 6: Population attributable fraction for risk factors for all-cause age-standardised years of life lost (YLLs) rate per 100,000 population per year, in order of decreasing Upper Tier Local Authority (UTLA) deprivation (Index of Multiple Deprivation [IMD]), England, both sexes, 2016

Upper Tier Local Authority	IMD score	All risk factors	Tobacco	Dietary risks	High systolic blood pressure	Alcohol and drug use	High body-mass index	High total cholesterol	Occupational risks	High fasting plasma glucose	Air pollution	Child and maternal malnutrition	Low physical activity	Impaired kidney function	Unsafe sex	Low bone mineral density	Other environmental risks	Sexual abuse and violence	Unsafe water, sanitation, and handwashing
**England (95% uncertainty interval)**	**NA**	**52.5 (50.6-54.4)**	**17.8 (17.1-18.7)**	**13.3 (11.9-14.8)**	**11.5 (10.3-12.7)**	**10.8 (7.9-14.3)**	**9.0 (5.8-12.3)**	**6.9 (5.8-8.1)**	**4.5 (4.0-5.1)**	**4.2 (2.9-5.7)**	**3.7 (3.0-4.4)**	**3.3 (3.1-3.4)**	**1.9 (1.0-2.8)**	**1.6 (1.5-1.7)**	**0.6 (0.6-0.6)**	**0.4 (0.3-0.4)**	**0.4 (0.2-0.6)**	**0.1 (0.1-0.1)**	**0.1 (0.0-0.1)**
Blackpool	42	56.1	21.4	14.5	12.2	12.6	8.7	7.3	4.4	4.2	4	2.8	1.9	1.5	0.7	0.5	0.4	0.1	0.1
Knowsley	41	57.7	26.2	14.3	11.8	9.9	9.3	7.3	5.9	4.2	4.5	2.2	1.9	1.6	0.7	0.5	0.6	0.1	0.1
Kingston upon Hull, City of	41	57	24.7	14.6	12.3	9.9	9.1	7.6	5.2	4.3	4.4	2.5	2	1.7	0.6	0.4	0.4	0.1	0
Liverpool	41	59.2	24.5	13	11	13.9	9	6.5	5.2	4	4.2	3.2	1.7	1.5	0.6	0.5	0.5	0.1	0.1
Manchester	41	60.5	24.4	13.9	12.4	13.8	10.2	7.5	5.2	4.5	4.5	3.2	2	1.6	0.7	0.6	0.5	0.1	0
Middlesbrough	40	57	24	14.4	12.1	10.4	9.4	7.3	5.6	4.4	4.1	2.6	1.9	1.8	0.8	0.4	0.4	0.1	0.1
Birmingham	38	56	18	13.9	11.5	11.7	9.3	7.2	4.3	4.6	3.8	6.2	1.9	1.6	0.5	0.5	0.4	0.1	0.1
Nottingham	37	55.7	21.1	13.4	11.6	11.1	9.5	7	5.1	4.5	4.1	4.2	1.9	1.5	0.6	0.4	0.7	0.1	0
Tower Hamlets	36	58.5	22	11.2	9.7	14	10.6	6.8	6.1	5.3	4.6	3.5	1.9	1.6	0.5	0.4	0.2	0.1	0
Hackney	35	56.2	18.6	10.8	11	13.5	10.8	6.4	4.3	5.6	4.3	4.3	1.8	1.8	0.8	0.3	0.2	0.1	0
Barking and Dagenham	35	55.1	23.1	14.1	12	8.6	8.2	7.3	6.5	4.2	4.7	2.6	1.9	1.6	0.7	0.4	0.2	0.1	0.1
Sandwell	35	54.9	19.7	14.6	12.1	9.8	9.3	7.4	4.6	5	3.9	4.4	2	1.7	0.6	0.4	0.5	0.1	0.1
Stoke-on-Trent	34	55.3	20.9	13.2	10.9	10.4	8.5	6.8	5.1	3.7	3.9	5.1	1.8	1.4	0.6	0.6	0.5	0.1	0.1
Blackburn with Darwen	34	56.4	21	15.5	13.2	10.6	9.5	8.2	4.7	4.6	4.1	3.5	2.2	1.9	0.6	0.4	0.4	0.1	0.1
Rochdale	34	56.5	21.9	15.4	13.1	10.6	9.4	8	5	4.6	4.1	2.7	2.1	1.9	0.7	0.4	0.5	0.1	0.1
Wolverhampton	33	55.2	18.9	14.5	12.1	10.9	9.5	7.2	4.7	5.1	3.7	4.3	1.9	1.8	0.5	0.4	0.4	0.1	0.1
Hartlepool	33	57.2	22.9	14.3	12.1	10.9	9.2	7.3	6	4.4	4	2.9	1.9	1.8	0.8	0.3	0.4	0.1	0.1
Bradford	33	54.9	19.7	14.5	12.5	10.3	8.9	7.7	4.7	4.2	4	4.3	2	1.7	0.6	0.3	0.4	0.1	0.1
Leicester	33	55.3	18.5	15	12.9	10.7	9.6	8.1	4.1	4.8	4.3	4.3	2.1	1.7	0.7	0.5	0.7	0.1	0.1
Salford	33	57.2	23.8	14.1	12.2	11.3	9.6	7.4	5.5	4.2	4.1	2.7	1.9	1.5	0.6	0.5	0.5	0.1	0.1
Newham	33	55.5	20.3	14.4	12.7	10.4	9.3	7.7	5.8	5	4.6	3	2	1.9	0.6	0.4	0.2	0.1	0.1
Islington	33	56.3	20.5	11	10.2	14.8	10	6.5	4.7	4.6	4.3	2.2	1.8	1.5	0.9	0.4	0.2	0.1	0
Halton	32	55.2	22.8	14	11.8	9.7	9.5	7.2	5	4.5	4.2	2.5	1.9	1.7	0.6	0.4	0.5	0.1	0.1
Haringey	31	53.1	17.2	13.3	12.1	12.2	9.1	6.9	4	4.8	4.1	3	1.9	1.8	0.7	0.4	0.2	0.1	0.1

North East Lincolnshire	31	54.3	20.2	14.7	12.4	10.3	9.8	7.8	4.4	4.8	4.1	2.7	2	2	0.7	0.3	0.4	0.1	0
South Tyneside	31	57.1	23.9	14.2	11.8	11.7	8.9	7	6.3	4.2	4.1	1.5	1.9	1.7	0.6	0.4	0.4	0.1	0.1
Walsall	30	54.7	18.4	14.7	11.8	9.8	9	7.5	4.6	4.9	3.7	5.7	1.9	1.8	0.5	0.4	0.4	0.1	0.1
Oldham	30	55.9	21.5	14.9	12.8	10	9	7.7	4.7	4.4	4.1	3.5	2.1	1.8	0.6	0.4	0.5	0.1	0.1
Waltham Forest	30	53.3	19.2	14.2	12.5	10.3	8.5	7.4	5	4.2	4.5	2.4	2	1.6	0.6	0.4	0.2	0.1	0.1
St Helens	30	55.5	22.2	14.8	12.4	10.4	9.1	7.6	5.2	4.1	4.3	2.3	2	1.7	0.7	0.5	0.5	0.1	0.1
Sunderland	30	56.4	22.7	14.1	11.9	11.1	9.4	7.3	6.2	4.2	3.6	2.5	1.9	1.7	0.6	0.4	0.4	0.1	0.1
Barnsley	30	54.2	21.2	15.4	12.9	8.6	9	8.1	4.9	4.5	4.3	2.4	2.1	1.6	0.7	0.3	0.4	0.1	0.1
Southwark	29	55.5	20.4	10.7	10.3	13	9.4	5.9	5	4.2	4.1	3.7	1.6	1.6	0.8	0.4	0.2	0.1	0
Tameside	29	57.5	22.8	16.3	13.7	10.3	9.7	8.6	5.5	4.4	4.6	2.1	2.3	1.7	0.6	0.5	0.5	0.1	0.1
Doncaster	29	54	21.3	13.8	11.6	9.4	8.4	7.1	5.5	4	4	3.3	1.9	1.5	0.6	0.3	0.4	0.1	0.1
Lambeth	29	55.1	19.1	11.3	10.8	13.9	9.1	5.9	4.4	4.3	3.9	3.7	1.6	1.6	0.7	0.4	0.2	0.1	0
Torbay	29	49.5	15.9	13.3	11.2	10.2	8.2	6.6	4	4.1	3.3	2.9	1.8	1.6	0.8	0.4	0.6	0.1	0
Lewisham	29	55.1	20.8	13.1	11.5	11.1	8.4	6.6	5.1	4.2	4.7	3.5	1.8	1.5	0.7	0.3	0.2	0.1	0.1
Redcar and Cleveland	29	55	22	15.2	12.7	9.6	9.5	7.7	5.1	4.5	3.9	2	2	1.9	0.8	0.4	0.4	0.1	0
Bolton	28	54.4	20.2	14.8	12.6	10.4	9.3	7.7	4.8	4.2	4.1	2.9	2	1.6	0.7	0.4	0.4	0.1	0.1
Rotherham	28	53.8	20.4	15.1	12.6	8.8	9.2	8	4.8	4.2	4.3	3.3	2.1	1.7	0.6	0.4	0.4	0.1	0
Newcastle upon Tyne	28	57.1	23.1	12.6	11.2	13.2	9.3	6.6	6.3	3.7	3.5	2.6	1.7	1.5	0.6	0.4	0.4	0.1	0
Coventry	28	54.5	19.4	13.6	11.5	11.7	9.6	6.8	4.4	4.9	3.7	3.3	1.8	1.6	0.6	0.4	0.5	0.1	0.1
Derby	28	53.5	18.8	13.8	11.9	9.5	9.5	7.5	4.9	4.3	4	4.3	2	1.5	0.5	0.4	0.7	0.1	0.1
Westminster	28	52.7	15.9	9.1	9.8	15.9	9.7	5.3	3.9	4.1	3.4	2.6	1.5	1.3	0.8	0.5	0.1	0.1	0
Peterborough	28	51.8	17.5	13.5	11.5	10.5	9.2	7.1	4.8	5.1	3.9	2.5	1.9	1.8	0.5	0.4	0.3	0.1	0.1
Luton	28	53.1	17.3	13.4	11.7	10.5	9	7.1	4.5	5	3.8	4	1.9	1.8	0.8	0.4	0.3	0.1	0.1
Sheffield	28	54	19.5	14	12.2	10.8	9.4	7.3	4.9	4.1	3.8	3.6	1.9	1.4	0.5	0.3	0.4	0.1	0
Bristol, City of	27	53.6	18.3	12.5	11.1	12.9	9.3	6.5	4.8	4	3.7	2.7	1.8	1.5	0.6	0.3	0.7	0.1	0.1
Portsmouth	27	54.5	19.9	13.6	11.9	11.7	9.3	7	5.3	4.5	4.1	1.7	1.9	1.7	0.6	0.4	0.4	0.1	0
Enfield	27	51.1	16.8	13.4	12.2	9.8	8.9	7.1	4.1	4.3	4	3.3	1.9	1.6	0.6	0.3	0.2	0.1	0.1
Wakefield	27	54.8	21.1	14.4	12.3	9.9	9.1	7.6	5.5	4.1	4.2	2.8	2	1.6	0.6	0.3	0.4	0.1	0.1
Wirral	27	54.1	20.3	13.6	11.4	11.5	8.5	6.6	4.8	4	3.6	3.1	1.8	1.6	0.7	0.4	0.4	0.1	0.1
Southampton	27	54.2	19.7	13.2	11.3	11.8	8.8	6.8	5.3	4	4	2.4	1.8	1.7	0.6	0.4	0.4	0.1	0
Brent	27	52.9	15.9	13.5	12.3	12	9.1	7.2	3.9	4.5	3.9	3.7	1.9	1.7	0.8	0.3	0.2	0.1	0
Plymouth	27	52.3	18.3	13.2	11.5	10.6	8.6	6.7	5	4.1	3.4	2.7	1.8	1.6	0.6	0.4	0.7	0.1	0
Leeds	27	56	20.6	14	12.2	11.7	9.7	7.7	5.4	4.1	4	3.2	2	1.6	0.6	0.4	0.4	0.1	0
Gateshead	26	56.5	22.4	13.9	12	11.2	9.4	7.3	5.8	4	3.8	3.1	1.9	1.6	0.7	0.4	0.4	0.1	0.1
County Durham	26	55.3	21.2	14.6	12.4	10.9	9.2	7.5	5	4.2	3.7	2.7	2	1.7	0.6	0.4	0.3	0.1	0.1
Sefton	26	54.8	21	13.7	11.4	11.7	8.6	6.7	4.5	3.8	3.7	3.1	1.8	1.5	0.6	0.4	0.5	0.1	0.1
Greenwich	26	54.8	20.9	13.4	11.7	10.4	8.5	6.8	5.5	4.3	4.2	3.5	1.8	1.7	0.6	0.3	0.2	0.1	0.1
Camden	25	54.1	16.8	9.2	8.3	17.2	9.4	5.5	4.2	4	3.6	2.7	1.5	1.4	0.9	0.4	0.1	0.1	0
Wigan	25	56.2	21.9	15.3	13.1	10.5	9.1	7.8	5.5	4.3	4.3	2.4	2.1	1.7	0.5	0.4	0.5	0.1	0.1
Telford and Wrekin	25	53.4	18.8	14.1	12	9.6	9.4	7.2	4.6	4.4	3.7	4.4	1.9	1.7	0.5	0.4	0.4	0.1	0
Stockton-on-Tees	25	55.3	20.8	13.9	12	10.7	9.6	7.2	5.5	4.2	3.6	3.4	1.9	1.7	0.7	0.4	0.3	0	0.1
Calderdale	25	53.7	20	14	12.3	9.8	9.2	7.3	5.1	4.4	3.8	3.1	2	1.8	0.6	0.3	0.4	0.1	0.1

Southend-on-Sea	25	51.1	17.7	13.2	11.4	10.4	8.3	6.7	4.5	4.2	3.7	2.5	1.8	1.5	0.7	0.3	0.3	0.1	0.1
Hammersmith and Fulham	24	54.2	19.3	10.9	10.2	13.6	9.5	6.1	4.6	4.3	3.8	3	1.6	1.6	0.8	0.4	0.2	0.1	0
Kirklees	24	53.6	18.6	14.4	12.7	9.5	9	7.5	4.8	4.4	3.8	4.1	2	1.8	0.6	0.3	0.4	0.1	0.1
Cornwall	24	49.9	14.6	13.1	11.4	10.5	8.3	6.7	3.6	3.9	3	4.1	1.8	1.6	0.6	0.4	0.5	0.1	0
Croydon	24	52.1	17.3	13.3	12.3	10.6	8.9	6.9	4.2	4.2	3.9	3.1	1.9	1.7	0.8	0.3	0.2	0.1	0
Darlington	24	53.1	19.4	14.1	12.3	10.5	9.9	7.5	5.2	4.2	3.6	2.7	1.9	1.5	0.6	0.4	0.3	0.1	0
Ealing	24	52.7	16.6	13.4	12.1	12.4	9.3	7.3	3.9	4.4	4	2.8	1.9	1.8	0.6	0.3	0.2	0.1	0.1
Brighton and Hove	23	52.1	16.7	11.2	9.9	14.1	8	5.4	3.8	3.8	3	3	1.6	1.4	1	0.4	0.3	0.1	0
Kensington and Chelsea	23	50.3	15.2	9.4	8.9	14.8	8.8	5.1	3.8	3.6	3.2	2.7	1.4	1.3	0.9	0.5	0.1	0.1	0
Isle of Wight	23	49.1	15.1	13.2	11.5	11	8.7	6.8	4.4	4	3.3	1.5	1.8	1.7	0.5	0.3	0.3	0.1	0
Dudley	23	51.9	17.4	13.8	11.4	10.1	8.8	6.7	4.4	4.3	3.6	4	1.8	1.5	0.5	0.4	0.4	0.1	0.1
Slough	23	52.7	18.2	13	11	10.7	9.4	7.4	4.3	4.5	4.2	3.2	2	1.5	0.4	0.4	0.4	0	0.1
Lancashire	22	55	18.8	13.8	12	12.8	9.2	7.2	4.3	3.9	3.7	3.5	1.9	1.6	0.5	0.4	0.4	0.1	0
Hounslow	22	52.7	17.6	12.3	11.2	11.7	9.2	6.8	4	4.4	4	3.5	1.8	1.7	0.7	0.3	0.2	0.1	0.1
Medway	22	52.9	19.5	13.2	11.3	9.7	8.1	6.4	5.6	4.3	3.9	2.5	1.8	1.8	0.6	0.4	0.4	0.1	0
Bournemouth	22	52	16.6	13.3	11.6	12.7	9.1	6.7	4	4.3	3.2	2.1	1.8	1.6	0.8	0.4	0.6	0.1	0
Bury	22	55.1	21.3	15	12.8	9.8	9.4	7.6	5	4.4	4.2	2.7	2	1.8	0.6	0.4	0.5	0.1	0.1
Thurrock	22	52.7	19.2	14	11.9	9.1	8.8	7.2	5.4	4.9	4.2	2.2	2	1.7	0.6	0.3	0.3	0.1	0.1
North Lincolnshire	21	53.2	19.3	14.8	12.4	9.1	9.7	7.6	4.9	5	4.1	2.9	2	2	0.7	0.3	0.4	0.1	0.1
Cumbria	21	52	17.5	13.9	12.1	11.3	9.4	7.2	4.3	4	3.2	2.6	1.9	1.7	0.6	0.4	0.4	0.1	0
North Tyneside	21	55.4	22.1	13.5	11.7	11.1	8.9	6.8	6.2	3.8	3.6	1.9	1.8	1.5	0.6	0.6	0.4	0.1	0.1
Norfolk	21	50.6	14.9	12.9	11.1	11.7	8.8	6.6	4	4.5	3.3	3.3	1.8	1.6	0.6	0.4	0.2	0.1	0
Lincolnshire	21	51.5	16.9	14.5	12.3	9.8	9.4	7.6	4.1	4.5	3.7	2.9	2	1.8	0.6	0.4	0.6	0.1	0
Northumberland	21	53.5	20.1	14.3	12.5	10.1	9.1	7.2	4.9	4	3.5	2.4	2	1.6	0.5	0.5	0.3	0.1	0.1
Redbridge	20	51.2	16.8	14.4	12.7	10.1	8.9	7.6	4.5	4.4	4.2	2.2	2	1.8	0.5	0.4	0.2	0.1	0.1
Herefordshire, County of	20	49	14.2	13.9	12.1	10.3	9.6	7	3.7	4.4	3.1	3	1.9	1.6	0.6	0.4	0.4	0.1	0
Reading	19	51.8	17.1	12	10.6	11.4	9.4	6.4	4.2	4.3	3.5	3.6	1.8	1.6	0.7	0.5	0.3	0.1	0.1
Warrington	19	54.2	20	13.9	12.4	9.8	10.1	7.5	4.8	4.4	4.1	3.3	2	1.6	0.6	0.4	0.4	0.1	0.1
Bedford	19	49.9	15.9	13.4	11.5	9.8	9	6.9	4.2	5	3.7	2.9	1.9	1.8	0.5	0.4	0.2	0.1	0.1
Stockport	19	53.6	19.7	14	12.2	10.2	9.6	7.2	4.8	3.9	3.9	3.4	1.9	1.4	0.6	0.5	0.4	0	0
Northamptonshire	19	52	17	12.6	11.2	10.8	8.9	6.4	4.4	4.1	3.6	3.8	1.8	1.6	0.6	0.4	0.6	0.1	0.1
Nottinghamshire	19	51.4	17.5	13.3	11.3	9.6	8.7	6.7	4.6	4.2	3.6	3.6	1.8	1.6	0.5	0.4	0.7	0.1	0.1
East Sussex	19	48.2	14.7	11.9	10.4	11.3	7.5	5.7	3.9	3.3	3.2	2.9	1.6	1.3	0.7	0.4	0.3	0.1	0
Kent	19	51.4	16.7	12.9	11	11.1	8.5	6.5	4.5	3.9	3.7	2.8	1.8	1.6	0.6	0.4	0.3	0.1	0
Derbyshire	19	52.1	17.2	14.2	12.1	10.1	9.2	7.4	4.4	4.2	3.6	2.9	2	1.5	0.5	0.5	0.7	0.1	0.1
Suffolk	18	49.5	14.9	13.4	11.4	10.2	8.9	6.8	4.2	4.6	3.4	2.8	1.9	1.5	0.6	0.4	0.2	0.1	0
Wandsworth	18	53.7	19.1	12.3	11.6	11.9	9.3	6.6	4.7	4.3	4	2.8	1.8	1.6	0.7	0.4	0.2	0.1	0
Hillingdon	18	51.9	17.6	12.1	11	11.4	9.3	6.7	4.8	4.3	3.8	2.8	1.8	1.5	0.5	0.3	0.2	0.1	0.1
Cheshire West and Chester	18	52.8	18.4	13.2	11.6	11	9.6	6.8	4.6	4	3.5	3.3	1.9	1.5	0.6	0.4	0.4	0.1	0.1
Milton Keynes	18	51.6	18	11.7	10.3	9.5	8.7	6.2	4.6	4.4	3.8	3.8	1.8	1.6	0.5	0.5	0.4	0	0.1
Havering	18	51.9	19.2	13.8	11.7	9.4	8.8	7.3	5.8	4	4.2	2.1	1.9	1.6	0.5	0.4	0.2	0.1	0
Swindon	18	52.5	18.3	13.3	11.5	9.8	9.4	7	5.2	4.5	3.8	2.8	1.9	1.6	0.5	0.3	0.7	0.1	0.1

Barnet	18	49.9	15.7	13	11.9	10.7	8.9	6.9	3.9	4.1	3.6	2.6	1.9	1.6	0.5	0.3	0.2	0.1	0
Somerset	18	48.2	13.9	12.5	10.8	10.2	8.4	6.2	3.7	4	3	3.5	1.7	1.5	0.6	0.4	0.5	0.1	0.1
Worcestershire	18	50	15.3	13.1	11.3	10.5	9	6.4	3.9	4	3.3	3.8	1.8	1.5	0.5	0.5	0.4	0.1	0.1
Solihull	17	49.8	16	12.9	10.8	9.9	9.5	6.5	4.2	4.1	3.3	3.7	1.8	1.5	0.5	0.5	0.4	0.1	0.1
Essex	17	51.2	16.6	13	11.1	10.3	8.7	6.5	4.6	4.5	3.6	3.3	1.8	1.6	0.6	0.4	0.3	0.1	0
Devon	17	48.9	13.8	12.7	11	10.9	8.6	6.4	3.7	3.8	2.9	3.5	1.8	1.5	0.6	0.4	0.5	0.1	0
Shropshire	17	50.4	15.9	14.1	11.9	10.2	9.4	7.1	4	4	3.3	3.1	1.9	1.5	0.5	0.3	0.4	0.1	0
Staffordshire	16	51.9	15.9	13.4	11.3	10.4	8.9	6.7	4.1	4.1	3.4	4.8	1.8	1.6	0.5	0.5	0.4	0.1	0.1
Bexley	16	52	19.3	13.8	11.9	9.6	8.8	7.1	5.4	3.9	4.2	2.3	1.9	1.5	0.5	0.3	0.2	0.1	0
East Riding of Yorkshire	16	50.6	17.6	14.4	12.5	9	9.3	7.4	4.4	4.3	3.7	2.2	2	1.8	0.6	0.4	0.3	0.1	0
North Somerset	16	48.7	14.6	12.7	11.2	10.6	8.6	6.3	3.8	4	3.2	2.7	1.8	1.6	0.5	0.3	0.6	0.1	0.1
Trafford	15	54.2	19.7	14	12.5	10.9	10.4	7.4	4.9	4.2	4.2	2.5	2	1.5	0.5	0.5	0.4	0.1	0
Poole	15	49.1	15.6	12.5	10.8	10.7	9	6.3	3.9	4	3.5	2.3	1.7	1.5	0.8	0.5	0.6	0.1	0
Bromley	15	50.6	16.9	13.3	12	10.3	9.2	6.9	4.7	4	3.6	2.1	1.9	1.6	0.6	0.3	0.2	0.1	0
Gloucestershire	15	49.8	14.7	12.7	11	11.2	8.8	6.6	4	4	3.3	2.8	1.8	1.5	0.6	0.5	0.6	0.1	0.1
Warwickshire	15	50.7	15.6	12.7	11	10.6	9.3	6.5	4	4.2	3.3	4.1	1.8	1.6	0.5	0.4	0.4	0.1	0.1
Merton	15	51.6	17.9	13.1	11.7	10.3	9.1	6.9	4.5	4	3.9	3	1.9	1.5	0.5	0.4	0.2	0.1	0
North Yorkshire	15	50.6	15.9	14.5	12.7	10	9.7	7.6	3.9	4.2	3.4	2.9	2	1.7	0.5	0.4	0.3	0.1	0
Sutton	15	50.6	18.3	12.8	11.4	10.1	8.5	6.7	5.1	3.6	4	2.3	1.8	1.4	0.4	0.3	0.2	0	0.1
Dorset	14	47.5	13.5	12.2	10.5	10.7	8.3	6.1	3.7	3.6	3	3.4	1.7	1.5	0.6	0.4	0.5	0.1	0
Harrow	14	49.9	14.8	13.2	11.6	9.8	8.6	7.1	3.8	3.9	3.7	4.6	1.9	1.5	0.5	0.3	0.2	0.1	0
Cheshire East	14	50.7	16.5	13	11.7	10.6	9.9	6.9	4.5	3.8	3.5	3.1	1.9	1.5	0.6	0.4	0.4	0.1	0
West Sussex	14	48.8	15	12.2	10.6	10.9	8.3	6.1	4	3.7	3.3	2.9	1.7	1.4	0.5	0.4	0.3	0.1	0
Wiltshire	13	48.1	14.1	12.5	11	10	8.5	6.3	4	4	3.2	3.2	1.8	1.5	0.6	0.4	0.5	0.1	0.1
Cambridgeshire	13	49.5	14.9	12.3	11.1	10.6	9.2	6.3	4.2	4.5	3.3	3	1.8	1.5	0.5	0.5	0.2	0.1	0.1
Leicestershire	12	50.4	15.4	13.3	11.5	9.8	9.2	7	4.1	4	3.5	4.1	1.9	1.5	0.6	0.4	0.6	0.1	0.1
York	12	51.7	18	14.3	12.7	9.8	10.2	7.6	4.8	4.1	3.8	2.3	2	1.7	0.5	0.4	0.3	0.1	0
Central Bedfordshire	12	49.7	16.5	13	11.3	9.6	8.8	6.5	4.6	4.6	3.6	2.1	1.9	1.7	0.5	0.5	0.3	0	0.1
Hertfordshire	12	50.8	16.6	12.6	11.3	10.5	9.3	6.5	4.5	4.4	3.7	2.7	1.8	1.5	0.5	0.4	0.2	0.1	0.1
Bath and North East Somerset	12	47.8	14.4	12.5	11.1	10.6	9.1	6.4	4.3	3.8	3.1	2.2	1.8	1.4	0.6	0.4	0.6	0.1	0.1
Hampshire	12	49.2	15	12.3	10.6	10.8	8.7	6.2	4.4	3.9	3.4	2.5	1.8	1.5	0.5	0.4	0.3	0.1	0
Oxfordshire	12	48.6	14.8	11.6	10	11.1	8.6	5.8	4	4	3.2	3	1.7	1.4	0.5	0.4	0.3	0.1	0.1
South Gloucestershire	11	47.8	14.8	12.2	10.8	9.8	9.3	6.4	4.3	3.9	3.2	2.4	1.8	1.5	0.6	0.3	0.6	0.1	0
Kingston upon Thames	11	50.1	16.3	12.4	11.6	11.1	9.2	6.6	4.2	3.8	3.8	2.7	1.8	1.6	0.5	0.3	0.2	0.1	0
Bracknell Forest	10	49.2	17.1	12	10.7	9.2	9	6.1	4.3	4.1	3.6	2.3	1.8	1.6	0.7	0.5	0.4	0.1	0.1
West Berkshire	10	48.4	15.7	11.4	10	10	9.2	5.8	4.6	4.2	3.3	2.3	1.7	1.5	0.5	0.4	0.3	0	0
Richmond upon Thames	10	49.2	15.3	11.1	10.2	12	8.7	5.8	3.8	3.6	3.6	3	1.6	1.4	0.6	0.3	0.1	0.1	0
Buckinghamshire	10	47.9	13.4	11.7	10.3	10.6	8.5	6	3.9	3.7	3.2	3.5	1.7	1.5	0.6	0.5	0.3	0.1	0
Rutland	10	49.7	13.8	13.8	12.3	10.1	9.7	7.6	3.6	3.7	3.5	4.1	2	2.3	0.2	0.5	0.5	0.1	0.1
Surrey	9	48.4	14.6	11.6	10.3	10.7	8.7	5.9	4.3	3.7	3.4	2.7	1.7	1.4	0.6	0.4	0.3	0.1	0.1

Windsor and Maidenhead	9	50.3	15.8	12.7	10.9	10.3	9.5	6.7	4.3	4.2	3.7	3	1.9	1.6	0.6	0.4	0.3	0.1	0.1
Wokingham	6	47.3	13.6	11.8	10.4	9.5	9	6	4	4	3.3	3.6	1.7	1.5	0.4	0.5	0.3	0	0.1

Statistically significantly lower than the England mean

Statistically significantly higher than the England mean

Appendix Figure 7. Annualised percentage change in all-cause age-standardised years of life lost (YLLs) rate over two time periods (2000-2009 and 2010-2016) by UK country and English Upper Tier Local Authorities (UTLA).

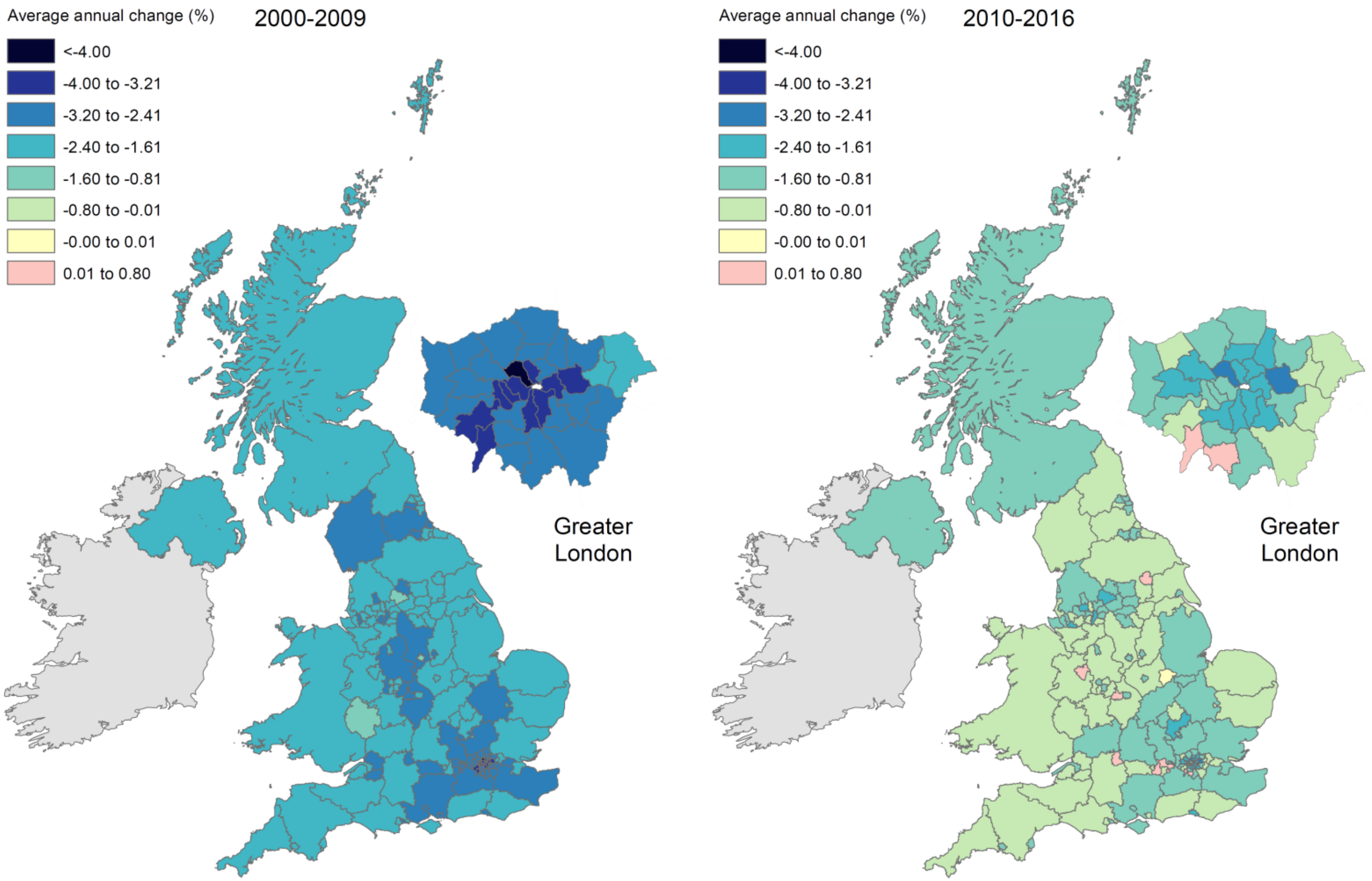

Data for the City of London and Isles of Scilly not available. Source: Institute for Health Metrics and Evaluation 2017. Contains National Statistics data © Crown copyright and database right 2017. Ireland outline © 2018 GADM. NISRA: Website: www.nisra.gov.uk. Contains NRS data © Crown copyright and database right 2017. Contains OS data © Crown copyright and database right 2017.

Appendix Figure 8: Absolute change in death rate per 100,000 population over time since 1990 by age group, UK

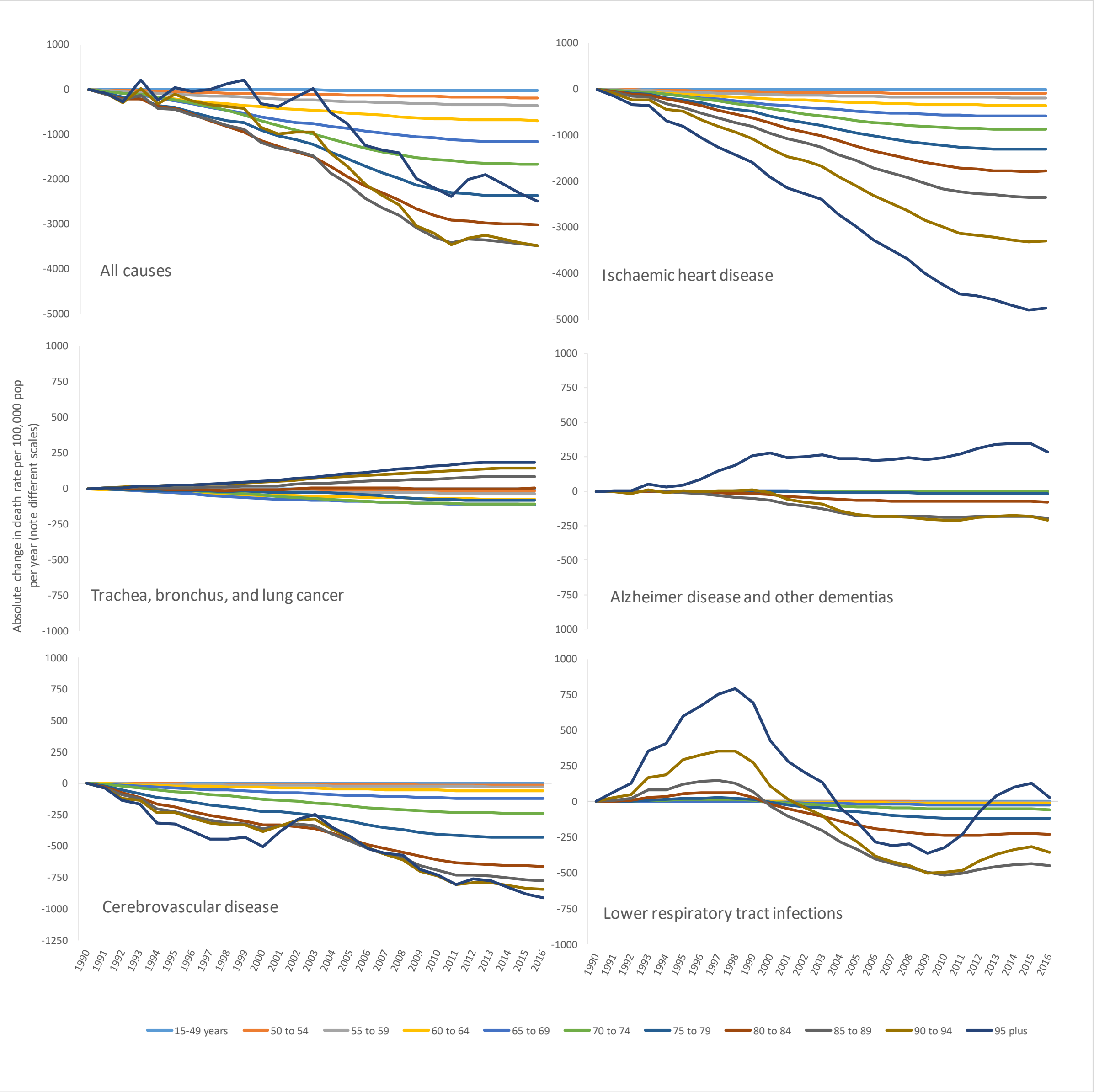

Appendix Figure 9: Absolute change in years of life lost (YLL) rate per 100,000 population over time since 1990 by age group, UK

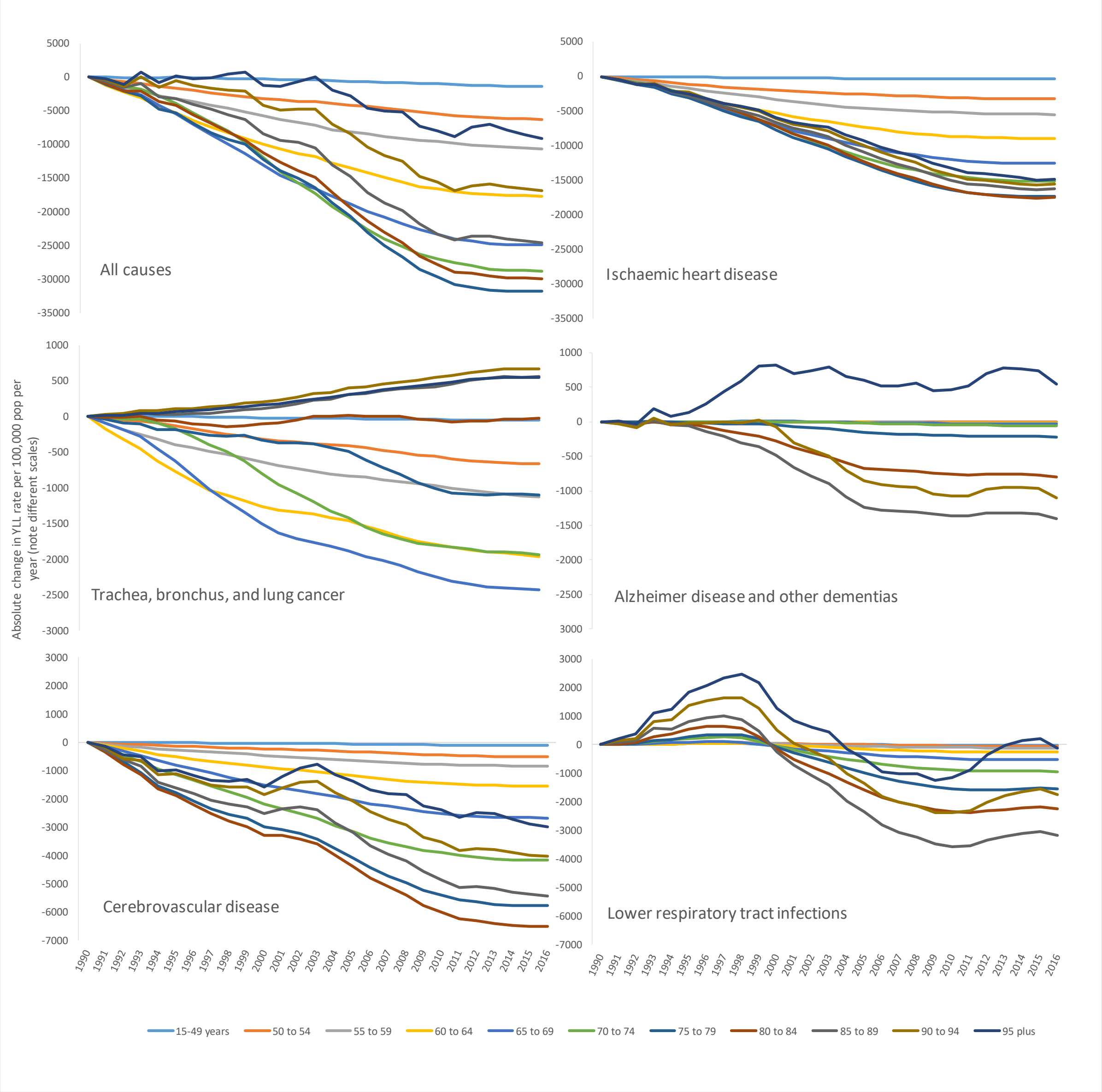

Appendix Figure 10: Mean annual change in attributable risk for all-cause age-standardised years of life lost (YLLs) rate per 100,000 population over three time periods, 1990-2000, 2000-2010, and 2010-2016 ranked by the highest rate, England

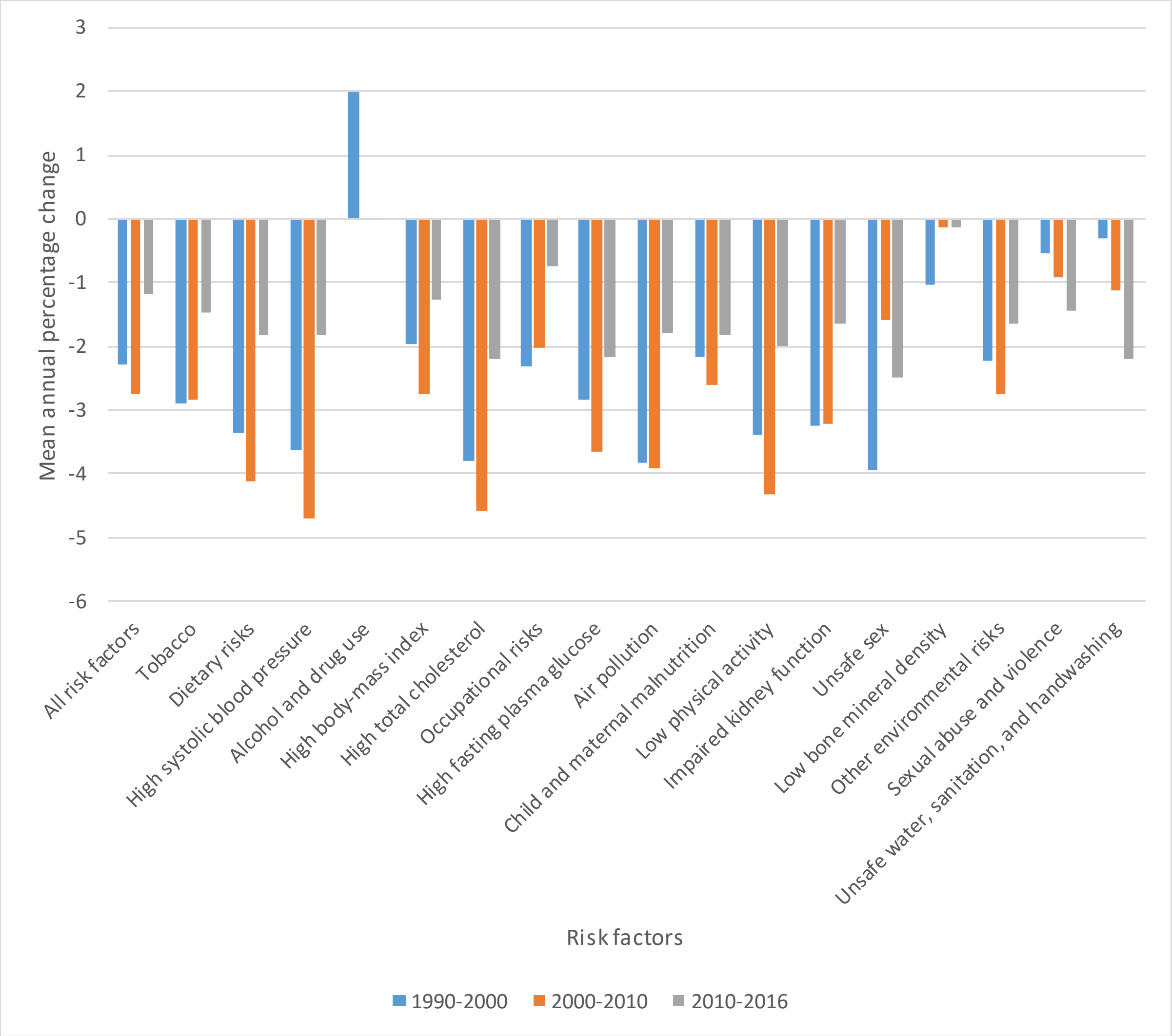

Appendix Figure 11: Mean annualised percentage change in all-cause age-standardised years of life lost (YLLs) over three time periods compared to Index of Multiple Deprivation (IMD) score, English Upper Tier Local Authorities (UTLAs) for both sexes combined

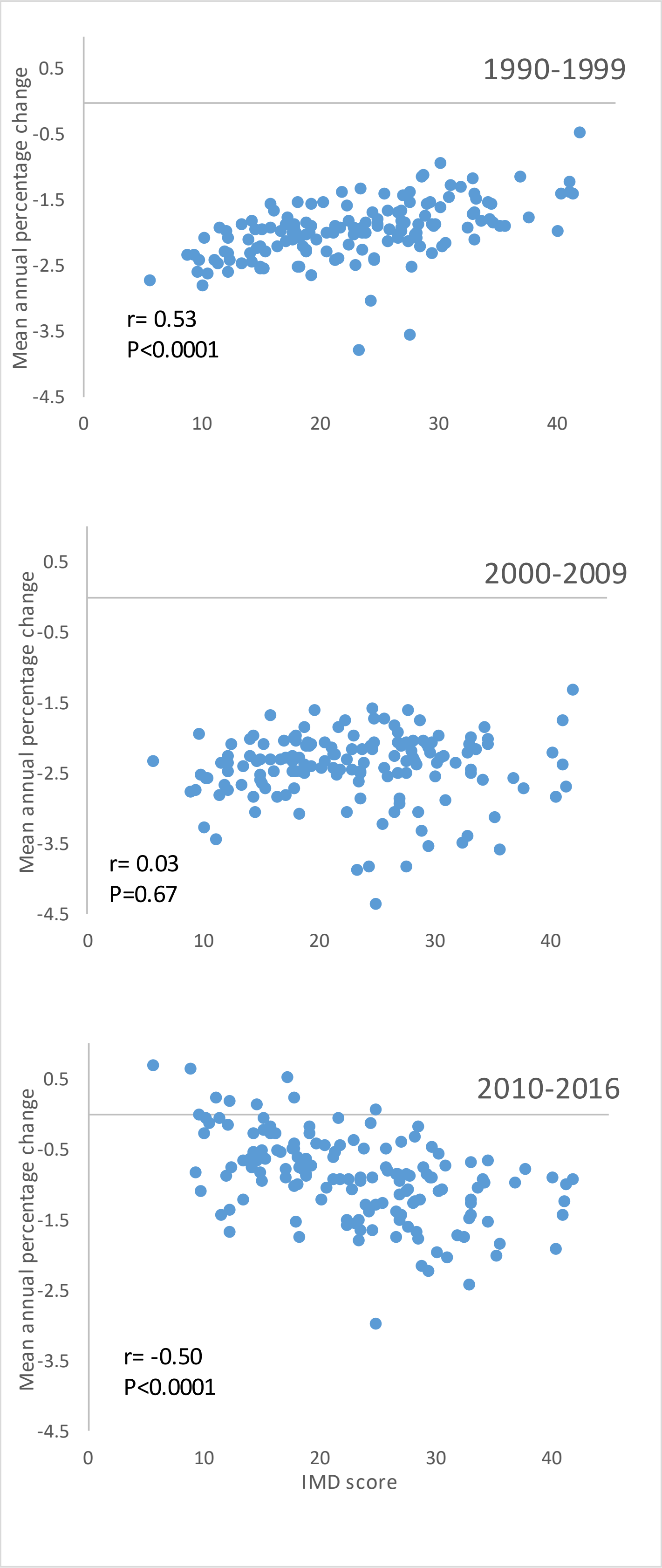

Appendix Figure 12: Mean annualised percentage change in all-cause age-standardised years of life lost (YLLs) rate per 100,000 population for the most affluent 10% and the least affluent 10% Upper Tier Local Authorities (UTLAs), England, both sexes, 1990 to 2016

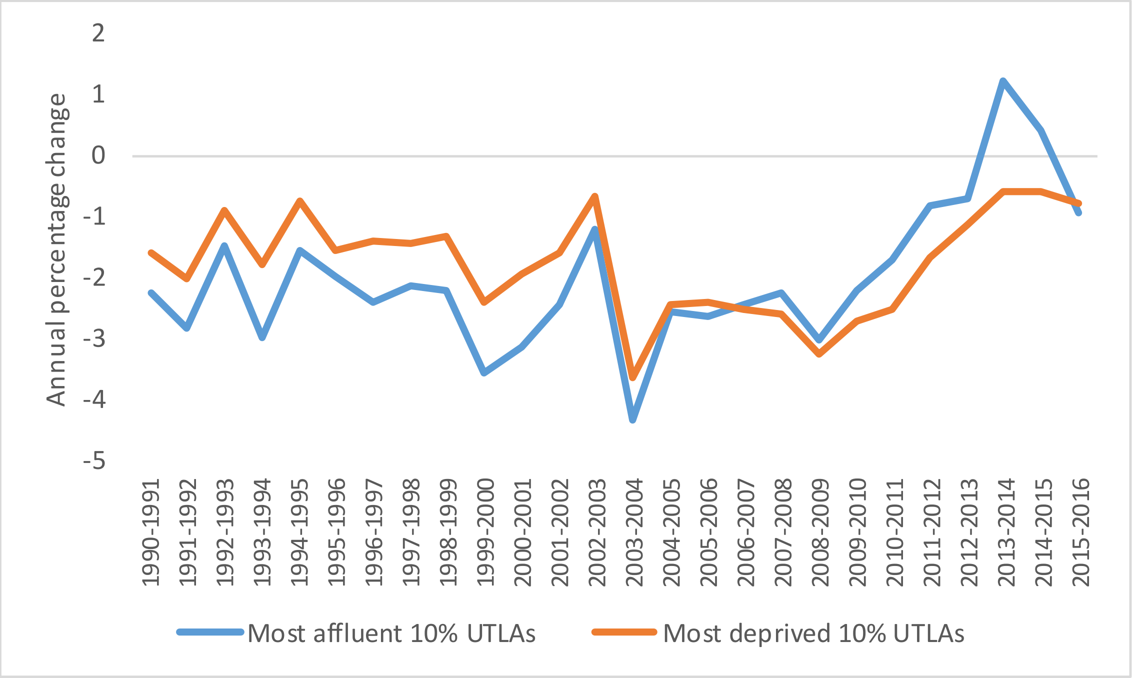

Appendix Figure 13: Age standardised years lived with disability (YLDs) rate per 100,000 population for the 10 causes with the highest YLD burden by Global Burden of Disease level, United Kingdom both sexes combined, 2016

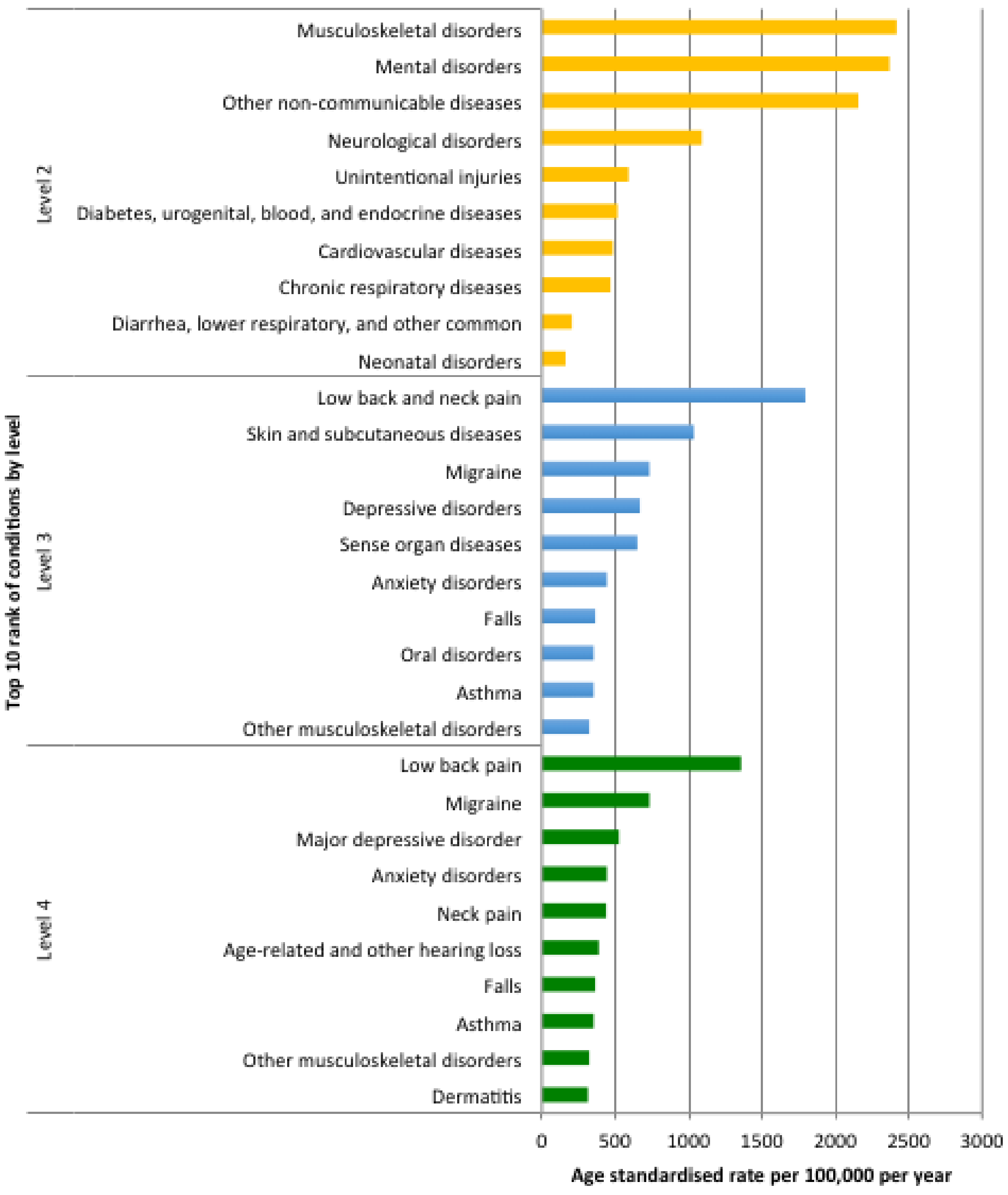

Data source identification and adjustment

Estimation of YLDs has eight major components. These are described more completely in the appendix of the GBD 2016 paper on non-fatal estimates[i], but the first two components include identifying and adjusting data in order to estimate prevalence and incidence for specific causes and sequelae for each geographic area.

Each cause has its own case definition and diagnostic criteria for identifying and accepting data that can be used in the modelling process. Although specific to each cause the general process includes:

a. Systematic selection of studies. These are undertaken for each cause and search terms are available in the appendix of the GBD paper on non-fatal estimates[ii]. Inclusion and exclusion criteria were applied to the studies returned.
b. In addition to study searches, additional data inputs for each cause were included depending on relevance and acceptability. Surveys, registry data, hospital data and surveillance system outputs are examples of this data. Generally, where case definition relevant individual-level data was available, this superseded data from study sources. Data sources included in models include country specific data, as well as data from non-country specific sources.

Prior to estimation of prevalence and incidence, many of the data sources undergo some form of adjustment and these are specific to the cause in question. For example, where different physiological measures are reported that differ from the case definition, then some adjustment to the reference definition will be undertaken. Age and sex splitting of the data is also carried on data sources where the required sex or age-groups are either not reported or too broad.

For example, Diabetes mellitus in the GBD 2016 was defined using a case definition of fasting plasma glucose (FPG) >126 mg/dL (7 mmol/L), or being on treatment for diabetes. However, the criteria accepted other measures of blood sugar (haemoglobin A1c [HbA1c], oral glucose tolerance test, or post prandial glucose test) to define diabetes. Data source input searching included a) systematic study searches; b) systematic search of the Global Health Data Exchange (GHDx) for survey programs and national surveys, as well as longitudinal studies tagged with either FPG or Diabetes mellitus; and c) datasets from other leaders in the field. The four data inputs include estimates of diabetes within a population, estimates of mean FPG, individual level data measured from surveys and Insurance claims data from the US. England specific data inputs accepted for the diabetes model include country-representative survey data (from the Health Survey for England) of blood measures and a number of published papers.

The data adjustment processes for diabetes included time, age and sex splitting of data sources, as well as adjustments to data points with alternative case-definitions in order to standardise data to the reference definition. For England survey data, blood glucose measures are reported as HbA1c. A reference of HbA1c of >6.5 was assumed to be the threshold equivalent to the GBD FPG case definition of diabetes.

The case definition for COPD is defined in GBD 2016 as in the Global Initiative for Chronic Obstructive Lung Disease (GOLD) classification: a measurement of <0.7 FEV_1/FVC (one second of forceful exhalation/total forced expiration) on spirometry after bronchodilation[iii]. This matches to a partial definition in the National Institute for Health and Care Excellence (NICE) guidelines used in England and Wales 101[iv], but NICE guidelines state where FEV_1 is $\geq$ 80% predicted normal a diagnosis of COPD should only be made in the presence of respiratory symptoms.

A systematic selection of studies were included using reported measures of COPD, excluding studies with smokers. An additional criteria for acceptance also included surveys, but only where COPD was reported using spirometry data and outcomes were within a plausible range. Where an alternative case definition was reported in accepted data sources, these were adjusted to GOLD classifications based on reference material linking definitions. The only England-specific data accepted with the search selection included one study based on measures taken from the Health Survey for England.

[i] GBD 2016. Disease and Injury Incidence and Prevalence Collaborators. Global, regional, and national incidence, prevalence, and years lived with disability for 328 diseases and injuries for 195 countries, 1990–2016: a systematic analysis for the Global Burden of Disease Study 2016. The Lancet. Volume 390, No. 10100, p1211–1259, 16 September 2017

[ii] GBD 2016. Disease and Injury Incidence and Prevalence Collaborators. Global, regional, and national incidence, prevalence, and years lived with disability for 328 diseases and injuries for 195 countries, 1990–2016: a systematic analysis for the Global Burden of Disease Study 2016. The Lancet. Volume 390, No. 10100, p1211–1259, 16 September 2017

[iii] GBD 2016. Disease and Injury Incidence and Prevalence Collaborators. Global, regional, and national incidence, prevalence, and years lived with disability for 328 diseases and injuries for 195 countries, 1990–2016: a systematic analysis for the Global Burden of Disease Study 2016. The Lancet. Volume 390, No. 10100, p1211–1259, 16 September 2017

[iv] NICE 2010. NICE Guideline 101: Chronic obstructive pulmonary disease in over 16s: diagnosis and management. https://www.nice.org.uk/guidance/cg101/chapter/Working-definition-of-COPD
